# Prevalence of Children Aged 6 to 23 Months Who Did Not Consume Animal Milk, Formula, or Solid or Semisolid Food During the Last 24 Hours Across Low- and Middle-Income Countries

**DOI:** 10.1001/jamanetworkopen.2023.55465

**Published:** 2024-02-12

**Authors:** Omar Karlsson, Rockli Kim, S. V. Subramanian

**Affiliations:** 1 Duke University Population Research Institute, Duke University, Durham, North Carolina; 2 Centre for Economic Demography, School of Economics and Management, Lund University, Lund, Sweden; 3 Division of Health Policy and Management, College of Health Science, Korea University, Seoul, Korea; 4 Harvard Center for Population and Development Studies, Cambridge, Massachusetts; 5 Interdisciplinary Program in Precision Public Health, Department of Public Health Sciences, Graduate School of Korea University, Seoul, Korea; 6 Department of Social and Behavioral Sciences, Harvard T.H. Chan School of Public Health, Harvard University, Boston, Massachusetts

## Abstract

**Importance:**

The introduction of solid or semisolid foods alongside breast milk plays a vital role in meeting nutritional requirements during early childhood, which is crucial for child growth and development. Understanding the prevalence of zero-food children (defined for research purposes as children aged 6 to 23 months who did not consume animal milk, formula, or solid or semisolid food during the last 24 hours) is essential for targeted interventions to improve feeding practices.

**Objective:**

To estimate the percentage of zero-food children in 92 low- and middle-income countries.

**Design, Setting, and Participants:**

This cross-sectional study analyzed nationally representative cross-sectional household data of children aged 6 to 23 months from the Demographic and Health Surveys and the Multiple Indicator Cluster Surveys conducted between May 20, 2010, and January 27, 2022. Data were obtained from 92 low- and middle-income countries. Standardized procedures were followed to ensure data comparability and reliability. Both percentage and number of zero-food children were estimated.

**Main Outcomes and Measures:**

The outcome studied was defined as a binary variable indicating children aged 6 to 23 months who had not been fed any animal milk, formula, or solid or semisolid foods during the 24 hours before each survey, as reported by the mother or caretaker.

**Results:**

A sample of 276 379 children aged 6 to 23 months (mean age, 14.2 months [95% CI, 14.15-14.26 months]) in 92 low- and middle-income countries was obtained, of whom 51.4% (95% CI, 51.1%-51.8%) were boys. The estimated percentage of zero-food children was 10.4% (95% CI, 10.1%-10.7%) in the pooled sample, ranging from 0.1% (95% CI, 0%-0.6%) in Costa Rica to 21.8% (95% CI, 19.3%-24.4%) in Guinea. The prevalence of zero-food children was particularly high in West and Central Africa, where the overall prevalence was 10.5% (95% CI, 10.1%-11.0%), and in India, where the prevalence was 19.3% (95% CI, 18.9%-19.8%). India accounted for almost half of zero-food children in this study.

**Conclusions and Relevance:**

In this cross-sectional study of 276 379 children aged 6 to 23 months, substantial disparities in the estimates of food consumption across 92 low- and middle-income countries were found. The prevalence of zero-food children underscores the need for targeted interventions to improve infant and young child feeding practices and ensure optimal nutrition during this critical period of development. The issue is particularly urgent in West and Central Africa and India.

## Key Points

**Question:**

What is the prevalence of zero-food children (ie, children who did not consume any animal milk, formula, or solid or semisolid food during the last 24 hours) across 92 low- and middle-income countries?

**Findings:**

In this cross-sectional study of 276 379 children aged 6 to 23 months in 92 low- and middle-income countries, 10.4% were zero-food children, ranging from 0.1% in Costa Rica to 21.8% in Guinea.

**Meaning:**

The high prevalence of zero-food children in some countries suggests a need for targeted interventions to improve infant and young child feeding practices to ensure optimal nutrition during a critical period of development.

## Teaser

This cross-sectional study examines the prevalence of zero-food children (children who do not consume any food other than breast milk) aged 6 to 23 months across 92 low- and middle-income countries.

## Introduction

Many children in low- and middle-income countries face challenges in accessing sufficient nutritious food due to various socioeconomic and environmental factors.^1^ As a result, undernutrition and micronutrient deficiencies continue to be significant public health concerns.^2^ Newborns and infants should rely on exclusive breastfeeding until aged 6 months. After age 6 months, breast milk alone is insufficient to meet the growing nutritional requirements of infants and young children.^3^ The introduction of adequate foods, in addition to continued breastfeeding, during this critical window is of paramount importance for optimal nutrition, overall health, and human development.^4,5^ Research has demonstrated the short- and long-term benefits of adequate feeding, such as reduced risk of mortality, malnutrition, stunting, underweight, and micronutrient deficiencies, as well as improved brain development, cognition, and school readiness, laying the foundation for future learning and achievement.^4,5,6^

Sustainable Development Goal (SDG) 2, one of 17 adopted by the United Nations in 2015, aims to end hunger and ensure access to adequate, safe, and nutritious food for everyone at all times by 2030.^7^ Policy goals typically rely on indicators such as minimum dietary diversity, which indicates the percentage of children who consume foods from at least 5 out of 8 food groups. For example, a recent report estimated that only 31% of children aged 6 to 23 months received minimum dietary diversity, and only 18% received minimum acceptable diet (ie, sufficient frequency and variety).^8^

Existing literature on malnutrition in low- and middle-income countries is extensive. However, few studies focus specifically on the subset of children aged 6 to 23 months who experienced complete absence of complimentary feeding.^9^ A recent study from India presented the term zero-food children, which refers to the alarming situation in which mothers report that their 6- to 23-month-old children did not receive any animal milk, formula, or solid or semisolid food within 24 hours prior to being surveyed.^9^ In the current article, we estimated the prevalence of zero-food children globally. By examining this extreme form of food insecurity, we strive to generate evidence-based recommendations that can inform policies, programs, and interventions aimed at improving the nutritional status and well-being of these vulnerable children. This study contributes to the monitoring of the progress of the SDGs on targets related to poverty and undernutrition, which are prominent in the SDGs.^10,11^ This study included a diverse group of children from 92 low- and middle-income countries that were at different stages of economic development and that faced different challenges regarding reliable food access. Thus, we hypothesized that there would be large disparities in the prevalence of zero-food children across countries.

## Methods

In this cross-sectional study, we used data from nationally representative cross-sectional household surveys conducted in low- and middle-income countries, specifically the Multiple Indicator Cluster Surveys (MICS) and the Demographic and Health Surveys (DHS).^12,13,14,15^ These surveys are highly standardized, and collaboration between agencies ensures comparability of the survey tools used in the MICS and the DHS.^16^ It should be noted that while the DHS collects information on feeding solely from biological mothers, the MICS collects information from both caretakers and biological mothers, thus including foster children. Furthermore, the MICS collects information on all children younger than age 3 years, while the DHS only collects information on 1 child younger than age 2 years in each household, generally the youngest. This study followed the Strengthening the Reporting of Observational Studies in Epidemiology (STROBE) reporting guidelines for cross-sectional studies. This study used publicly accessible secondary data obtained from the DHS and the MICS; the activities did not meet the regulatory definition of human participant research according to the Harvard Longwood Campus institutional review board decision tool, and informed consent was not required.

For our analysis, we considered surveys conducted after January 1, 2010. Studies were conducted over 1 to 3 calendar years, with the oldest survey starting on May 20, 2010, and the most recent survey completed on and January 27, 2022. Only a single survey was used for each country—the most recent survey when more than 1 survey was available. We included only nationally representative surveys and excluded those focused on specific subpopulations or subnational regions. In total, we pooled data from 92 countries: 49 from the MICS and 43 countries from the DHS (eTable 1 in Supplement 1). All countries included in the analysis were low- or middle-income countries as classified by the World Bank at the time of survey.^17^

Both the DHS and the MICS generally use a stratified, multistage sampling approach. This involves selecting primary sampling units (commonly villages or neighborhoods) from strata of geographic or administrative subnational regions, further divided into urban and rural areas. Then a systematic random sampling method is used to select 20 to 30 households from each unit. Women aged 15 to 49 years are interviewed in these households, providing information on their health, the health of their children, and child feeding. The response rates for the surveys typically exceed 90%.^12,14^ Sampling weights are calculated to account for nonresponse and oversampling, as well as to improve precision.^14,18^ In most surveys, information on feeding is obtained from mothers or caretakers for children younger than age 2 or 3 years. Therefore, we restricted our analysis to children younger than age 24 months. Additionally, we excluded children younger than age 6 months, who should be exclusively breastfed.

### Outcome

The outcome studied was defined as a binary variable indicating children aged 6 to 23 months who had not been fed any animal milk, formula, or solid or semisolid food during the 24 hours before each survey, as reported by the mother (or caretaker). Both the DHS and the MICS included standardized questions on the feeding of common foods, country-specific foods, or any other solid or semisolid foods. The questions varied between the MICS and the DHS, and sometimes changed across waves (surveys conducted within the same countries at different times), but mostly included the same broad food groups and were always comprehensive with regard to the consumption of solid or semisolid foods (see eTable 2 in Supplement 1). Milk and infant formula were considered food items, while breastfeeding and feeding of other liquids, such as clear broth, juice, tea, or coffee, were not (although broth and juice were considered in a sensitivity analysis). We also present results for the percentage of children who were both zero food and not being breastfed (ie, were not fed any animal milk, formula, solid or semisolid food, or breast milk and may have consumed only other liquids or even nothing at all).

### Supplemental Analyses

We conducted 5 supplemental analyses. First, we considered the percentage of children who were zero food and also not fed juice or broth. Second, we also considered the percentage of children who were zero food, not fed juice or broth, and not being breastfed. Third, we estimated the prevalence of zero-food children across age groups 6 to 11 months, 12 to 17 months, and 18 to 23 months. Fourth, we present data on the prevalence of zero-food children separately for boys and girls. Fifth, we present data on the prevalence of zero-food children across quintiles of household wealth, constructed by the DHS and the MICS using principal component analysis on a household’s ownership of assets and access to amenities.

### Statistical Analysis

We first defined a binary variable for zero food, indicating whether a child aged 6 to 23 months did not receive any animal milk, formula, or solid or semisolid food 24 hours before a survey, as reported by a mother or a caretaker. The percentage of zero food was estimated for each country. We first created a box plot of the distribution of the prevalence of zero food across countries, both pooled and grouped by World Bank income groups (at the time of the survey).^17^

We tabulated the zero-food prevalence for each country. We also tabulated the prevalence by region and for the pooled sample. We estimated the number of children not receiving any animal milk, formula, or solid or semisolid food in each country using our estimated prevalence of zero-food children and an estimate of the population of children aged 6 to 23 months in the country and year of the survey. The United Nations world population prospects provides population estimates for single-year age groups, which were linked to the year of each survey.^19^ We estimated the number of children aged 6 to 11 months by multiplying the proportion of children aged 0 to 11 months in our sample who were aged 6 to 11 months with the United Nations’ population estimates for infants aged 0 to 11 months. Then that number was added to the United Nations’ population estimates for children aged 1 year to obtain the number of children aged 6 to 23 months.

We calculated the Spearman rank correlation coefficient between the zero-food prevalence and the number of zero-food children and categorized countries according to the number of children (many or few) and prevalence (high or low) based on whether countries fell below or above the median on each measure. All estimates were weighted using sampling weights that were rescaled to sum the population of children aged 6 to 23 months in each country and year of the survey, using population estimates from the United Nations world population prospects.^19^ All 95% CIs (logit transformed) were based on SEs adjusted for clustering at the level of primary sampling units. Analyses were performed using Stata, version 16 (StataCorp LLC).

## Results

**Figure 1.**
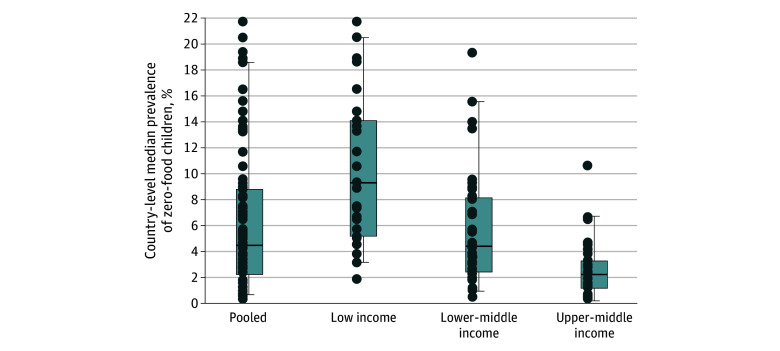
Distribution of Zero-Food Prevalence Across Countries According to Income Group All countries included in the analysis were low- or middle-income groups as classified by the World Bank at the time of the surveys.^17^ Each country’s estimate was weighted using sampling weights. Surveys were equally weighted for the median and percentiles. Dots indicate country estimates; horizontal lines inside boxes, medians; and outer horizontal box lines, the 25th and 75th percentiles. Ends of the vertical lines indicate the 5th and 95th percentiles. See Table 1 for tabulated estimates.

In total, data were obtained for 288 407 children aged 6 to 23 months, of whom 276 379 (95.8%; mean age, 14.2 months [95% CI, 14.15-14.26 months]) had valid information provided on any of the feeding variables (ie, children may have had missing information on some of the feeding variables and still have been included in the study). Of the total participants analyzed, 51.4% (95% CI, 51.1%-51.8%) were boys, and 48.6% (95% CI, 48.2%-48.9%) were girls. Across the 92 low- and middle-income countries in our sample, the country-level median percentage of zero-food children was 4.4% (Figure 1). The country-level median prevalence of zero-food was the greatest in low-income countries (9.3%), followed by lower-middle income countries (4.3%), while it was the lowest in upper-middle income countries (2.2%).

In the pooled sample, the estimated prevalence of zero-food children was 10.4% (95% CI, 10.1%-10.7%), which translated to 13.9 million children aged 6 to 23 months, across all 92 countries (Table 1). The highest prevalence, as well as the largest number of children (among countries included in our study), was 15.7% (95% CI, 15.2%-16.2%), observed in South Asia, which translated to 8 million children. The second-highest prevalence, as well as number of children, was 10.5% (95% CI, 10.1%-11.0%), observed in West and Central Africa, which corresponded to 2.9 million children. The third-greatest prevalence was 7.8% (95% CI, 6.9%-8.8%), observed in Eastern and Southern Africa, which suggests that there were 1.9 million zero-food children from the countries included in our sample.

**Table 1.** Zero-Food Prevalence and Estimated Number of Zero-Food Children Across Regions and Countries^a^

Location	Year	Zero-food children prevalence, % (95% CI)^b^	Zero-food children, No., thousands^c^
Pooled	NA	10.4 (10.1-10.7)	13 903
South Asia	NA	15.7 (15.2-16.2)	8032
India	2019-2021	19.3 (18.9-19.8)	6732
Pakistan	2017-2018	9.2 (7.2-11.8)	849
Afghanistan	2015	8.8 (7.5-10.3)	162
Bangladesh	2019	5.6 (5.0-6.3)	251
Nepal	2019	4.2 (3.3-5.4)	36
Maldives	2016-2017	2.4 (1.2-4.7)	0.3
West and Central Africa	NA	10.5 (10.1-11.0)	2939
Guinea	2018	21.8 (19.3-24.4)	144
Mali	2018	20.5 (18.4-22.7)	243
Benin	2017-2018	18.8 (17.2-20.6)	113
Liberia	2019-2020	18.6 (16.1-21.4)	42
Burkina Faso	2010	16.5 (15.0-18.1)	156
Guinea-Bissau	2018-2019	14.1 (12.3-16.1)	12
Mauritania	2019-2021	14.0 (12.4-15.7)	29
Central African Republic	2018-2019	14.0 (12.2-15.9)	41
Niger	2012	13.6 (12.0-15.3)	161
Senegal	2019	13.5 (10.9-16.6)	102
Chad	2019	13.3 (11.9-14.8)	132
Sierra Leone	2019	10.5 (9.1-12.1)	38
Cote d’Ivoire	2016	9.5 (8.1-11.1)	115
Togo	2017	9.3 (7.4-11.6)	34
Ghana	2017-2018	8.9 (7.4-10.7)	116
Nigeria	2021	8.8 (7.9-9.8)	962
Cameroon	2018-2019	8.2 (6.9-9.7)	106
Democratic Republic of the Congo	2017-2018	7.4 (6.4-8.7)	362
Gambia	2019-2020	7.3 (6.1-8.8)	9
Sao Tome and Principe	2019	7.0 (5.0-9.7)	1
Congo	2014-2015	5.4 (4.4-6.7)	13
Gabon	2012	2.2 (1.6-3.0)	2
Eastern and Southern Africa	NA	7.8 (6.9-8.8)	1861
Ethiopia	2019	14.8 (11.3-19.1)	772
Sudan	2014	11.6 (10.4-13.1)	219
Angola	2015-2016	10.5 (9.3-12.0)	172
Comoros	2012	6.6 (4.7-9.3)	2
Mozambique	2011	6.5 (5.5-7.8)	87
Namibia	2013	6.4 (5.0-8.3)	6
Uganda	2016	5.7 (4.9-6.5)	118
Burundi	2016-2017	5.1 (4.4-6.0)	31
Rwanda	2019-2020	5.0 (4.1-6.1)	28
Kenya	2014	4.6 (3.7-5.8)	93
Malawi	2019-2020	4.5 (3.7-5.5)	41
Madagascar	2021	4.5 (3.6-5.5)	57
South Africa	2016	4.5 (2.9-6.8)	78
Zambia	2018-2019	4.5 (3.7-5.4)	41
Zimbabwe	2019	3.4 (2.6-4.5)	24
Lesotho	2018	2.4 (1.4-4.0)	2
Tanzania	2015-2016	1.8 (1.3-2.6)	51
Eswatini	2014	0.9 (0.4-1.8)	0.4
Middle East and North Africa	NA	4.6 (4.2-5.1)	424
Egypt	2014	6.8 (6.0-7.8)	255
Yemen	2013	4.3 (3.5-5.2)	57
Jordan	2017-2018	3.7 (2.8-5.0)	13
Algeria	2018-2019	3.3 (2.6-4.1)	49
Iraq	2018	2.8 (2.2-3.5)	46
State of Palestine	2019-2020	1.4 (0.9-2.1)	3
Tunisia	2018	0.5 (0.2-1.2)	2
Europe and Central Asia	NA	4.0 (3.4-4.8)	125
Tajikistan	2017	9.3 (7.7-11.1)	35
Albania	2017-2018	6.7 (4.3-10.3)	3
Kazakhstan	2015	4.6 (3.0-7.2)	27
Uzbekistan	2021-2022	3.7 (2.2-6.1)	43
Armenia	2015-2016	3.1 (1.9-5.2)	2
Kyrgyzstan	2018	2.6 (1.7-4.0)	6
Montenegro	2018	1.8 (0.9-3.9)	0.2
Turkmenistan	2019	1.4 (0.8-2.4)	3
Georgia	2018	1.1 (0.5-2.8)	1
North Macedonia	2018-2019	0.7 (0.1-4.6)	0.2
Serbia	2019	0.7 (0.2-2.7)	1
Belarus	2019	0.4 (0.2-1.1)	1
Kosovo	2019-2020	0.2 (0-1.7)	0.1
East Asia and the Pacific	NA	2.9 (2.5-3.3)	339
Timor-Leste	2016	15.6 (13.4-18.0)	7
Papua New Guinea	2016-2018	8.1 (6.7-9.8)	28
Myanmar	2015-2016	5.5 (4.0-7.6)	75
Samoa	2019-2020	4.1 (2.8-5.9)	0.4
Cambodia	2014	3.7 (2.7-5.1)	19
Lao	2017	2.9 (2.4-3.6)	7
Kiribati	2018-2019	2.4 (1.5-3.9)	0.1
Vietnam	2020-2021	2.3 (1.4-3.7)	51
Indonesia	2017	2.2 (1.7-2.7)	151
Mongolia	2018	1.7 (1.0-2.8)	2
Fiji	2021	1.4 (0.7-2.6)	0.3
Tonga	2019	1.1 (0.4-3.2)	0.04
Tuvalu	2019-2020	0.7 (0.1-4.6)	0
Latin America and the Caribbean	NA	1.9 (1.3-2.8)	120
Guyana	2014	4.4 (3.0-6.4)	1
Guatemala	2014-2015	3.8 (3.1-4.7)	22
Haiti	2016-2017	3.1 (2.3-4.2)	12
Belize	2015-2016	2.9 (1.6-5.1)	0.3
Suriname	2018	2.8 (1.7-4.5)	0.4
Peru	2012	2.2 (1.7-2.9)	19
Paraguay	2016	2.2 (1.3-3.7)	4
Honduras	2019	1.9 (1.3-2.6)	6
Cuba	2019	1.6 (1.0-2.4)	3
Mexico	2015	1.5 (0.5-3.9)	46
El Salvador	2014	1.2 (0.7-1.9)	2
Dominican Republic	2019	1.1 (0.6-2.0)	3
Costa Rica	2018	0.1 (0-0.6)	0.1

Abbreviation: NA, not applicable.

^a^ Zero-food children were those aged 6 to 23 months who did not consume any animal milk, formula, or solid or semisolid food. The number of zero-food children was estimated using the estimated zero-food prevalence and the population of children aged 6 to 23 months obtained from the United Nations world population prospects^19^ and linked to the country and year of the survey.

^b^ 95% CIs were adjusted for clustering at the level of primary sampling units.

^c^ Estimates were weighted using sampling weights rescaled to sum the population of children aged 6 to 23 months in the country and year of the survey.

The lowest prevalence, as well as number of zero-food children, was 1.9% (95% CI, 1.3%-2.8%) observed in Latin America and the Caribbean, which translated to 120 000 children. The second-lowest prevalence was 2.9% (95% CI, 2.5%-3.3%), observed in East Asia and the Pacific (339 000 zero-food children). Guinea was the country with the greatest prevalence of zero-food children (21.8% [95% CI, 19.3%-24.4%]), followed by Mali (20.5% [95% CI, 18.4%-22.7%]) and India (19.3% [95% CI, 18.9%-19.8%]). Costa Rica had the lowest prevalence of zero-food children (0.1% [95% CI, 0%-0.6%]), followed by Kosovo (0.2% [95% CI, 0%-1.7%]).

**Figure 2.**
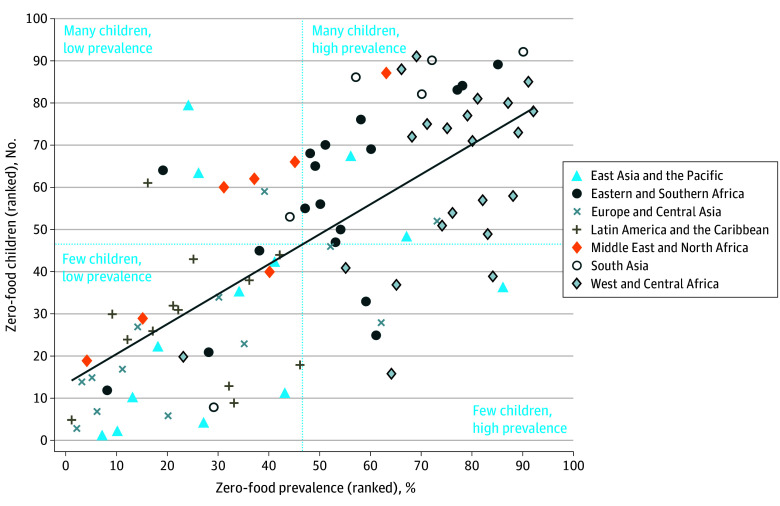
Correlation Between Country-Level Zero-Food Prevalence and the Number of Zero-Food Children Zero-food children were those aged 6 to 23 months who did not consume any animal milk, formula, or solid or semisolid food. The Spearman rank correlation coefficient was *r* = 0.3. Dashed blue vertical and horizontal lines indicate the median country for each variable. The solid diagonal line is a line of best fit. The number of zero-food children was estimated using the estimated zero-food prevalence and the population of children aged 6 to 23 months obtained from the United Nations world population prospects^19^ and linked to the country and year of the survey. See Table 2 for tabulated estimates.

There was a small positive Spearman rank correlation (*r* = 0.3) between the number and prevalence of zero-food children across countries (Figure 2). India had by far the largest number of zero-food children (6.7 million), almost half of all zero-food children in the 92 countries included in this study (Table 2). Nigeria had the second-highest number of zero-food children (962 000), followed by Pakistan (849 000), Ethiopia (772 000), and the Democratic Republic of the Congo (362 000). Several populous countries had relatively many zero-food children despite having a low prevalence, especially Indonesia, which had a 2.2% prevalence, or 150 900 children. When considering the percentage of children who were zero food and also not being breastfed (ie, may have consumed only other liquids or even nothing at all), the prevalence in the pooled sample was reduced to less than 1%, with the highest prevalence observed in Guinea (3.6% [95% CI, 2.7%-4.9%]), followed by Timor-Leste (2.7% [95% CI, 2.0%-3.8%]) and Angola (2.5% [95% CI, 1.9%-3.3%]) (Table 3).

**Table 2.** Categorization of Countries by the Estimated Number of Zero-Food Children and Zero-Food Prevalence^a^

Country	No., thousands (%)
**Many children and high prevalence**	
India	6732.2 (19.3)
Nigeria	961.9 (8.8)
Pakistan	848.5 (9.2)
Ethiopia	771.9 (14.8)
Democratic Republic of the Congo	362.4 (7.4)
Egypt	254.5 (6.8)
Bangladesh	250.8 (5.6)
Mali	243.3 (20.5)
Sudan	218.8 (11.6)
Angola	172.2 (10.5)
Afghanistan	162.2 (8.8)
Niger	161.1 (13.6)
Burkina Faso	156.4 (16.5)
Guinea	144.5 (21.8)
Chad	131.8 (13.3)
Uganda	118.1 (5.7)
Ghana	115.5 (8.9)
Cote d’Ivoire	115.5 (9.5)
Benin	113.1 (18.8)
Cameroon	105.9 (8.2)
Senegal	101.8 (13.5)
Kenya	92.7 (4.6)
Mozambique	86.7 (6.5)
South Africa	77.6 (4.5)
Myanmar	74.8 (5.5)
Madagascar	56.6 (4.5)
Liberia	41.7 (18.6)
Central African Republic	40.8 (14.0)
Malawi	40.8 (4.5)
Zambia	40.6 (4.5)
Sierra Leone	37.7 (10.5)
Tajikistan	35.0 (9.3)
Togo	34.4 (9.3)
Burundi	31.2 (5.1)
Mauritania	29.1 (14.0)
Papua New Guinea	27.8 (8.1)
Rwanda	27.5 (5.0)
**Many children and low prevalence**	
Indonesia	150.9 (2.2)
Yemen	57.0 (4.3)
Tanzania	51.5 (1.8)
Vietnam	50.6 (2.3)
Algeria	49.2 (3.3)
Mexico	46.5 (1.5)
Iraq	46.2 (2.8)
Uzbekistan	43.0 (3.7)
Nepal	35.8 (4.2)
**Few children and high prevalence**	
Kazakhstan	27.2 (4.6)
Congo	13.4 (5.4)
Guinea-Bissau	12.4 (14.1)
Gambia	9.3 (7.3)
Timor-Leste	7.3 (15.6)
Namibia	6.2 (6.4)
Albania	3.1 (6.7)
Comoros	2.2 (6.6)
Sao Tome and Principe	0.7 (7.0)
**Few children and low prevalence**	
Zimbabwe	23.9 (3.4)
Guatemala	22.5 (3.8)
Peru	19.4 (2.2)
Cambodia	18.7 (3.7)
Jordan	13.2 (3.7)
Haiti	12.3 (3.1)
Lao	7.0 (2.9)
Kyrgyzstan	6.5 (2.6)
Honduras	5.8 (1.9)
Paraguay	4.4 (2.2)
Dominican Republic	3.3 (1.1)
State of Palestine	3.1 (1.4)
Turkmenistan	2.7 (1.4)
Cuba	2.5 (1.6)
El Salvador	2.0 (1.2)
Armenia	2.0 (3.1)
Mongolia	2.0 (1.7)
Lesotho	1.8 (2.4)
Gabon	1.8 (2.2)
Tunisia	1.5 (0.5)
Guyana	1.0 (4.4)
Georgia	1.0 (1.1)
Serbia	0.7 (0.7)
Belarus	0.6 (0.4)
Suriname	0.4 (2.8)
Eswatini	0.4 (0.9)
Samoa	0.4 (4.1)
Fiji	0.3 (1.4)
Belize	0.3 (2.9)
Maldives	0.3 (2.4)
North Macedonia	0.2 (0.7)
Montenegro	0.2 (1.8)
Costa Rica	0.1 (0.1)
Kiribati	0.1 (2.4)
Kosovo	0.1 (0.2)
Tonga	0.04 (1.1)
Tuvalu	0.002 (0.7)

^a^ High and low were defined relative to the median country. Zero-food children were those aged 6 to 23 months who did not consume any animal milk, formula, or solid or semisolid food. The number of zero-food children was estimated using the estimated zero-food prevalence and the population of children aged 6 to 23 months obtained from the United Nations World Population Prospects and linked to the country and year of survey.

**Table 3.** Prevalence and Estimated Number of Zero-Food and Nonbreastfed Children Across Regions and Countries^a^

Location	Year	Zero-food and nonbreastfed children prevalence, % (95% CI)^b^	Zero-food and nonbreastfed children, No., thousands^c^
Pooled	NA	0.7 (0.7-0.8)	969
South Asia	NA	1.1 (1.0-1.2)	570
India	2019-2021	1.5 (1.4-1.7)	527
Afghanistan	2015	0.5 (0.4-0.8)	9
Pakistan	2017-2018	0.3 (0.1-0.9)	28
Maldives	2016-2017	0.2 (0.1-0.7)	0.03
Bangladesh	2019	0.1 (0-0.2)	3
Nepal	2019	0.1 (0-0.3)	1
Eastern and Southern Africa	NA	0.8 (0.6-1.1)	200
Angola	2015-2016	2.5 (1.9-3.3)	41
Ethiopia	2019	1.6 (0.9-2.8)	84
South Africa	2016	1.6 (0.8-3.1)	27
Comoros	2012	1.0 (0.4-2.6)	0.3
Lesotho	2018	0.7 (0.3-2.0)	1
Mozambique	2011	0.6 (0.4-1.0)	8
Namibia	2013	0.6 (0.3-1.2)	1
Madagascar	2021	0.5 (0.3-0.9)	6
Sudan	2014	0.3 (0.2-0.7)	6
Kenya	2014	0.3 (0.1-0.6)	5
Tanzania	2015-2016	0.3 (0.1-0.7)	7
Uganda	2016	0.2 (0.1-0.5)	4
Malawi	2019-2020	0.2 (0.1-0.4)	2
Zambia	2018-2019	0.2 (0.1-0.4)	2
Burundi	2016-2017	0.1 (0-0.3)	1
Rwanda	2019-2020	0.1 (0-0.3)	0.4
Zimbabwe	2019	0 (0-0.2)	0.2
Eswatini	2014	NA	0
West and Central Africa	NA	0.5 (0.4-0.6)	141
Guinea	2018	3.6 (2.7-4.9)	24
Central African Republic	2018-2019	1.5 (1.1-2.2)	4
Mauritania	2019-2021	1.5 (1.0-2.1)	3
Benin	2017-2018	1.4 (1.0-1.9)	8
Mali	2018	1.2 (0.9-1.8)	15
Cameroon	2018-2019	0.9 (0.6-1.4)	12
Chad	2019	0.9 (0.6-1.3)	9
Sierra Leone	2019	0.8 (0.5-1.3)	3
Liberia	2019-2020	0.8 (0.3-2.2)	2
Congo	2014-2015	0.7 (0.4-1.3)	2
Niger	2012	0.6 (0.3-1.1)	7
Burkina Faso	2010	0.5 (0.3-0.8)	5
Democratic Republic of the Congo	2017-2018	0.4 (0.2-0.7)	19
Gabon	2012	0.3 (0.1-0.7)	0.3
Nigeria	2021	0.2 (0.1-0.3)	21
Guinea-Bissau	2018-2019	0.2 (0.1-0.4)	0.2
Cote d’Ivoire	2016	0.2 (0-0.7)	2
Senegal	2019	0.1 (0-0.7)	1
Ghana	2017-2018	0.1 (0-0.3)	1
Gambia	2019-2020	0.1 (0-0.6)	0.1
Togo	2017	NA	0
Sao Tome and Principe	2019	NA	0
Middle East and North Africa	NA	0.3 (0.2-0.4)	27
Jordan	2017-2018	0.7 (0.4-1.4)	3
Algeria	2018-2019	0.4 (0.2-0.9)	7
Egypt	2014	0.3 (0.2-0.6)	12
Yemen	2013	0.2 (0.1-0.5)	3
Iraq	2018	0.2 (0.1-0.3)	3
State of Palestine	2019-2020	0 (0-0.2)	0.1
Tunisia	2018	NA	0
Europe and Central Asia	NA	0.2 (0.1-0.3)	5
Tajikistan	2017	0.7 (0.4-1.4)	3
Albania	2017-2018	0.6 (0.2-1.4)	0.3
Armenia	2015-2016	0.2 (0-1.2)	0.1
Uzbekistan	2021-2022	0.1 (0-0.9)	1
Kyrgyzstan	2018	0.1 (0-0.4)	0.2
Kazakhstan	2015	NA	0
Turkmenistan	2019	NA	0
Belarus	2019	NA	0
Serbia	2019	NA	0
Georgia	2018	NA	0
North Macedonia	2018-2019	NA	0
Kosovo	2019-2020	NA	0
Montenegro	2018	NA	0
East Asia and the Pacific	NA	0.1 (0.1-0.2)	15
Timor-Leste	2016	2.7 (2.0-3.8)	1
Samoa	2019-2020	0.7 (0.3-1.6)	0.1
Papua New Guinea	2016-2018	0.6 (0.3-1.0)	2
Myanmar	2015-2016	0.3 (0.1-1.2)	5
Cambodia	2014	0.2 (0.1-0.4)	1
Mongolia	2018	0.1 (0-0.7)	0.1
Indonesia	2017	0.1 (0-0.2)	5
Vietnam	2020-2021	0.1 (0-0.3)	1
Lao	2017	0 (0-0.1)	0.05
Fiji	2021	NA	0
Kiribati	2018-2019	NA	0
Tonga	2019	NA	0
Tuvalu	2019-2020	NA	0
Latin America and the Caribbean	NA	0 (0-0.1)	3
Dominican Republic	2019	0.4 (0.1-1.1)	1
Cuba	2019	0.3 (0.1-0.6)	0.5
Honduras	2019	0.2 (0.1-0.7)	1
Suriname	2018	0.1 (0-0.9)	0.02
Paraguay	2016	0.1 (0-0.5)	0.2
Haiti	2016-2017	0 (0-0.2)	0.1
Guatemala	2014-2015	0 (0-0.1)	0.1
Peru	2012	0 (0-0.1)	0.1
Mexico	2015	NA	0
El Salvador	2014	NA	0
Costa Rica	2018	NA	0
Guyana	2014	NA	0
Belize	2015-2016	NA	0

Abbreviation: NA, not applicable.

^a^ Zero food and nonbreastfed children were not fed any animal milk, formula, solid or semisolid food or breast milk. The number of zero-food and nonbreastfed children was estimated using the estimated zero-food and nonbreastfed prevalence and the population of children aged 6 to 23 months obtained from the United Nations world population prospects^19^ and linked to the country and year of the survey.

^b^ 95% CIs were adjusted for clustering at the level of primary sampling units.

^c^ Estimates were weighted using sampling weights rescaled to sum the population of children aged 6 to 23 months in the country and year of the survey.

### Results From Supplemental Analyses

The percentage of zero-food children who also did not consume juice or broth was lower than the prevalence of zero-food children, for example, 9.3% (95% CI, 9.1%-9.6%) compared with 10.4% in the pooled sample (eTable 3 in Supplement 1). The percentage of zero-food children who also did not consume juice or broth and were not being breastfed was very low, for example, 0.6% (95% CI, 0.6%-0.7%) in the pooled sample (eTable 4 in Supplement 1).

There was a clear age gradient in the prevalence of zero food in most regions. For example, in the pooled sample, 20.0% (95% CI, 19.4%-20.5%) were zero food among children aged 6 to 11 months, which declined to 6.6% (95% CI, 6.2%-6.9%) among those aged 12 to 17 months and to 4.1% (95% CI, 3.8%-4.6%) among those aged 18 to 23 months (eTable 5 in Supplement 1). There was no difference in the prevalence of zero-food children for boys and girls in the pooled sample (eTable 6 in Supplement 1). Although there were regions in which the prevalence differed between boys and girls, the 95% CIs were generally highly overlapping. We observed a strong gradient according to household wealth. For example, in the pooled sample, among the lowest quintile of household wealth, 13.4% (95% CI, 12.8%-14.0%) had zero-food prevalence, while the prevalence was 6.9% (95% CI, 6.3%-7.6%) in the highest quintile of household wealth (eTable 7 in Supplement 1).

## Discussion

This cross-sectional study sheds light on the prevalence of zero-food children aged 6 to 23 months in 92 low- and middle-income countries who did not receive any animal milk, formula, or solid or semisolid food. Our findings underscore the urgent need for comprehensive interventions to address the nutritional challenges faced by these children with food insecurity. Our study found that 10.4% of children in this age group—a critical period of growth and development when children are particularly vulnerable to undernutrition—were zero-food children who did not receive any animal milk, formula, or complementary food in the 24 hours prior to the survey. The pooled sample of 92 countries translated to 13.9 million zero-fed children in the studied countries. The prevalence of zero-food children varied across regions, with the highest prevalences observed in South Asia (15.7%) and West and Central Africa (10.5%).

Of particular concern was the significant number of zero-food children in South Asia, accounting for 8 million children—mostly in India—followed by West and Central Africa with 2.9 million children. These numbers highlight the magnitude of the challenge and the urgent need for tailored interventions to address this issue in these regions. Furthermore, Eastern and Southern Africa exhibited a prevalence of 7.8%, translating to 1.9 million children. The prevalence in Latin America and the Caribbean, East Asia and the Pacific, and Europe and Central Asia was lower, but efforts should still be directed toward supporting the nutritional needs of all affected children.

India emerged as the country with the highest number of zero-food children, representing almost half of the total number from the 92 countries in the study. Nigeria, Pakistan, Ethiopia, and the Democratic Republic of the Congo also showed substantial numbers of zero-food children, emphasizing the need for targeted interventions in these countries. Populous countries such as Indonesia and the Philippines had relatively many zero-food children, despite relatively lower prevalence rates. This highlights the importance of considering both prevalence and absolute numbers when designing interventions, even in countries with apparently lower risk.

We did find that the percentage of children who were both zero food and not being breastfed was less than 1%, which suggests that few children did not receive any calories. Furthermore, a supplemental analysis indicated that the prevalence of zero-food children declined sharply among older children, suggesting that prolonged, exclusive breastfeeding may have been an important factor. However, the energy requirements of children rise as they age. For example, at ages 6 to 8 months, children need 600 kcal per day, which increases to 700 kcal at ages 9 to 11 and 900 kcal at ages 12 to 23 months.^20^ At ages 12 to 23 months, food should constitute about 61% of the energy consumed. At approximately age 6 months, breastfeeding is no longer sufficient to provide the child with necessary nutrition.^21,22^ At that point, children need safe food with adequate protein, energy, vitamins, and minerals in combination with breastfeeding, which can continue until the age of 23 months or older. Estimates suggest that 31% of children in low- and middle-income countries do not receive what is considered minimum dietary diversity, and only 18% of children are fed a minimum acceptable diet.^8^ We identified the prevalence of children who not only did not receive a minimum acceptable diet but also did not receive any animal milk, formula, or complementary food 24 hours before being surveyed.

Our study identifies critical areas for policy and programmatic interventions aimed at improving the nutritional status and well-being of zero-food children. Addressing the socioeconomic and environmental factors that contribute to food insecurity is essential.^23^ Strategies should include enhancing access to nutritious foods,^23^ improving maternal and caregiver knowledge about appropriate feeding practices, and strengthening health systems to ensure the availability of necessary resources and support.^24^ The design and implementation of interventions should be context specific, accounting for cultural practices and local challenges.^24^

Further research is needed to understand the underlying causes of zero-food prevalence and its association with various socioeconomic factors. We found differences according to household wealth. Longitudinal studies could provide valuable insights into the short-term and long-term consequences of inadequate feeding on child health and development. Additionally, qualitative research may help uncover barriers and facilitators to optimal adequate feeding practices, thereby informing the development of effective interventions.

### Limitations

While this study provides valuable insights into the prevalence of zero-food children in low- and middle-income countries, it is important to acknowledge several limitations that may affect the interpretation and generalizability of the findings.

First, the study relied on self-reported data obtained from household surveys. The accuracy of parental or caregiver recall regarding the child’s food consumption in the 24 hours preceding the survey may have been subject to recall bias, leading to potential underestimation or overestimation of zero-food prevalence. Additionally, cultural and social desirability biases could have impacted the reporting of food-consumption practices, potentially affecting the validity of the results.

Second, the study used cross-sectional data; the observed prevalence of zero-food children did not provide information on the duration or persistence of inadequate feeding practices. Longitudinal studies would be valuable in examining the dynamics and patterns of feeding over time. Furthermore, using the data in our study, we could not assess the underlying reason for a child not being fed; for example, some children may have been sick or convalescent and therefore did not received any solid or semisolid foods.

Third, the study did not include all low- and middle-income countries. While countries in the study were geographically diverse and representative of different regions, the findings may not be fully representative of the entire low- and middle-income country population. Large low- and middle-income countries such as China, Russia, Brazil, and Iran were not included. The selection of countries was also dependent on the availability of data, which could have introduced selection bias.

Fourth, the study relied on aggregated, national-level data, which may have masked within-country variations. For example, a recent study found large differences in zero-food prevalence across states in India.^9^ Subnational disparities in zero-food prevalence and associated factors may exist, which could have important implications for targeted interventions. Future studies should explore subnational data to provide a more granular understanding of the problem.

Fifth, the study did not address the underlying causes of zero-food prevalence. While socioeconomic and environmental factors are likely variables, this study does not provide a comprehensive analysis of the determinants of inadequate feeding practices. Further research is needed to investigate the complex interplay of individual, household, and contextual factors influencing these practices.

Sixth, some surveys, most notably in India from 2019 to 2021, may have been impacted by the COVID-19 pandemic, which may have caused an increased prevalence of zero-food children. Seventh, the season of the survey may have impacted the prevalence of zero-food children in some countries. Eighth, the study does not assess the quality and adequacy of the foods consumed by children who were not categorized as zero-food children. Furthermore, while children consuming infant formula were not considered zero food in this study, infant formula alone is considered insufficient for children older than age 6 months. Understanding the nutritional adequacy of the diet is crucial for designing targeted interventions.

Ninth, we used data from 2 different sources. Although the DHS and the MICS collaborate and are highly similar in their implementation, an important difference is that the MICS interviews both biological mothers and caretakers while the DHS only interviews biological mothers on the feeding of children.

## Conclusions

The findings of this cross-sectional study highlight the prevalence of zero-food children in low- and middle-income countries and emphasized the urgent need for targeted interventions to improve feeding practices. Addressing the nutritional needs of these vulnerable children is critical for their overall health, growth, and future well-being. By implementing evidence-based policies and programs, policymakers can strive to reduce the prevalence of zero-food children and ensure a healthier and more prosperous future for the next generation.

## Notes

Supplement 1. **eTable 1.** Full Samples and Final Samples **eTable 2.** Variable Labels for Questions on Complementary Feeding **eTable 3.** Zero-Food-Juice-Broth Prevalence and Estimated Number of Zero-Food-Juice-Broth Children **eTable 4.** Zero-Food-Juice-Broth and Nonbreastfed Prevalence and Estimated Number of Zero-Food-Juice-Broth and Nonbreastfed Children **eTable 5.** Zero-Food Prevalence: By Child’s Age **eTable 6.** Zero-Food Prevalence: By Child’s Sex **eTable 7.** Zero-Food Prevalence: By Household Wealth Quintile

Supplement 2.
Data Sharing Statement
