# Supplementary material for: Prevalence of Children Aged 6 to 23 Months Who Did Not Consume Animal Milk, Formula, or Solid or Semisolid Food During the Last 24 Hours Across Low- and Middle-Income Countries
Source: JAMA Netw Open. 2024 Feb 12;7(2):e2355465. doi: 10.1001/jamanetworkopen.2023.55465 (PMC10862155; doi:10.1001/jamanetworkopen.2023.55465)
Supplement: Supplement 1. — eTable 1. Full Samples and Final Samples eTable 2. Variable Labels for Questions on Complementary Feeding eTable 3. Zero-Food-Juice-Broth Prevalence and Estimated Number of Zero-Food-Juice-Broth Children eTable 4. Zero-Food-Juice-Broth and Nonbreastfed Prevalence and Estimated Number of Zero-Food-Juice-Broth and Nonbreastfed Children eTable 5. Zero-Food Prevalence: By Child’s Age eTable 6. Zero-Food Prevalence: By Child’s Sex eTable 7. Zero-Food Prevalence: By Household Wealth Quintile [file jamanetwopen-e2355465-s001.pdf]

Supplemental Online Content

Karlsson O, Kim R, Subramanian SV. Prevalence of children aged 6 to 23 months who did not consume animal milk, formula, or solid or semisolid food during the last 24 hours across low- and middle-income countries. *JAMA Netw Open*. 2024;7(2):e2355465. doi:10.1001/jamanetworkopen.2023.55465

eTable 1. Full Samples and Final Samples
eTable 2. Variable Labels for Questions on Complementary Feeding
eTable 3. Zero-Food-Juice-Broth Prevalence and Estimated Number of Zero-Food-Juice-Broth Children
eTable 4. Zero-Food-Juice-Broth and Nonbreastfed Prevalence and Estimated Number of Zero-Food-Juice-Broth and Nonbreastfed Children
eTable 5. Zero-Food Prevalence: by Child's Age
eTable 6. Zero-Food Prevalence: by Child's Sex
eTable 7. Zero-Food Prevalence: by Household Wealth Quintile

This supplemental material has been provided by the authors to give readers additional information about their work.

© 2024 Karlsson O et al. *JAMA Network Open*.

eTable 1. Full Samples and Final Samples

	Survey year	Data source	Full sample	Final sample	% remaining	Population '000
Pooled			288,407	276,379	95.8	133,257.2
East Asia & Pacific			22,536	21,953	97.4	11,740.6
Cambodia	2014	DHS	2,190	2,126	97.1	499.7
Fiji	2021	MICS	671	665	99.1	24.9
Indonesia	2017	DHS	5,181	4,996	96.4	6,879.7
Kiribati	2018–19	MICS	669	662	99.0	5.1
Lao	2017	MICS	3,428	3,428	100.0	236.9
Mongolia	2018	MICS	1,674	1,655	98.9	116.9
Myanmar	2015–16	DHS	1,372	1,336	97.4	1,356.6
Papua New Guinea	2016–18	DHS	2,660	2,503	94.1	343.6
Samoa	2019–20	MICS	865	860	99.4	8.6
Timor-Leste	2016	DHS	2,073	1,981	95.6	47.1
Tonga	2019	MICS	367	359	97.8	3.7
Tuvalu	2019–20	MICS	169	166	98.2	0.4
Viet Nam	2020–21	MICS	1,217	1,216	99.9	2,217.6
Eastern & Southern Africa			50,579	46,467	91.9	23,898.2
Angola	2015–16	DHS	4,144	4,029	97.2	1,633.3
Burundi	2016–17	DHS	3,995	3,886	97.3	610.0
Comoros	2012	DHS	907	863	95.1	32.5
Eswatini	2014	MICS	789	789	100.0	45.9
Ethiopia	2019	DHS	1,543	1,478	95.8	5,229.5
Kenya	2014	DHS	5,373	2,795	52.0	2,012.8
Lesotho	2018	MICS	1,009	987	97.8	77.1
Madagascar	2021	DHS	3,598	3,479	96.7	1,259.8
Malawi	2019–20	MICS	4,744	4,731	99.7	906.5
Mozambique	2011	DHS	3,475	3,282	94.4	1,324.1
Namibia	2013	DHS	1,475	1,298	88.0	96.0
Rwanda	2019–20	DHS	2,352	2,289	97.3	552.4
South Africa	2016	DHS	994	871	87.6	1,735.0
Sudan	2014	MICS	4,064	4,038	99.4	1,878.2
Tanzania	2015–16	DHS	3,110	2,991	96.2	2,815.3
Uganda	2016	DHS	4,391	4,164	94.8	2,082.8
Zambia	2018–19	DHS	2,879	2,781	96.6	908.9
Zimbabwe	2019	MICS	1,737	1,716	98.8	698.1
Europe & Central Asia			12,912	10,874	84.2	3,090.7
Albania	2017–18	DHS	777	772	99.4	46.5
Armenia	2015–16	DHS	523	518	99.0	63.1
Belarus	2019	MICS	988	988	100.0	140.8
Georgia	2018	MICS	701	700	99.9	83.8
Kazakhstan	2015	MICS	1,632	1,632	100.0	585.5
Kosovo	2019–20	MICS	471	471	100.0	29.2
Kyrgyzstan	2018	MICS	992	989	99.7	249.3
Montenegro	2018	MICS	332	330	99.4	10.9
North Macedonia	2018–19	MICS	456	455	99.8	32.5
Serbia	2019	MICS	570	568	99.6	99.7
Tajikistan	2017	DHS	1,730	1,700	98.3	377.4
Turkmenistan	2019	MICS	1,058	1,055	99.7	199.9
Uzbekistan	2021–22	MICS	2,682	696	26.0	1,171.9
Latin America & Caribbean			24,902	24,647	99.0	6,321.4
Belize	2015–16	MICS	730	730	100.0	10.9
Costa Rica	2018	MICS	1,035	1,032	99.7	103.6
Cuba	2019	MICS	1,628	1,622	99.6	161.2
Dominican Republic	2019	MICS	2,593	2,576	99.3	301.4
El Salvador	2014	MICS	2,266	2,266	100.0	170.0
Guatemala	2014–15	DHS	3,583	3,542	98.9	589.0
Guyana	2014	MICS	1,034	1,032	99.8	23.0
Haiti	2016–17	DHS	1,779	1,656	93.1	395.2
Honduras	2019	MICS	2,542	2,530	99.5	311.1
Mexico	2015	MICS	2,311	2,311	100.0	3,162.3
Paraguay	2016	MICS	1,443	1,443	100.0	202.2
Peru	2012	DHS	2,776	2,729	98.3	875.8

© 2024 Karlsson O et al. *JAMA Network Open*.

	Survey year	Data source	Full sample	Final sample	% remaining	Population '000
Suriname	2018	MICS	1,182	1,178	99.7	15.8
Middle East & North Africa			24,063	23,820	99.0	9,123.9
Algeria	2018–19	MICS	4,362	4,360	100.0	1,498.8
Egypt	2014	DHS	4,888	4,834	98.9	3,736.1
Iraq	2018	MICS	4,786	4,763	99.5	1,663.3
Jordan	2017–18	DHS	2,750	2,712	98.6	354.1
State of Palestine	2019–20	MICS	1,923	1,914	99.5	219.4
Tunisia	2018	MICS	946	934	98.7	322.3
Yemen	2013	DHS	4,408	4,303	97.6	1,329.9
South Asia			85,223	83,244	97.7	51,154.7
Afghanistan	2015	DHS	8,296	8,026	96.7	1,841.1
Bangladesh	2019	MICS	6,691	6,672	99.7	4,469.8
India	2019–21	DHS	64,060	62,535	97.6	34,796.7
Maldives	2016–17	DHS	868	846	97.5	11.3
Nepal	2019	MICS	1,986	1,964	98.9	849.1
Pakistan	2017–18	DHS	3,322	3,201	96.4	9,186.7
West & Central Africa			68,192	65,374	95.9	27,927.7
Benin	2017–18	DHS	4,015	3,886	96.8	599.9
Burkina Faso	2010	DHS	4,303	4,144	96.3	948.5
Cameroon	2018–19	DHS	2,739	2,579	94.2	1,289.8
Central African Republic	2018–19	MICS	2,527	2,488	98.5	292.2
Chad	2019	MICS	5,446	5,413	99.4	994.0
Congo	2014–15	MICS	2,765	2,764	100.0	246.9
Congo DR	2017–18	MICS	6,499	6,499	100.0	4,867.9
Cote d'Ivoire	2016	MICS	2,668	2,668	100.0	1,212.5
Gabon	2012	DHS	1,832	1,695	92.5	82.4
Gambia	2019–20	DHS	2,389	2,311	96.7	127.0
Ghana	2017–18	MICS	2,585	2,575	99.6	1,293.4
Guinea	2018	DHS	2,077	1,942	93.5	664.2
Guinea-Bissau	2018–19	MICS	2,193	2,193	100.0	87.9
Liberia	2019–20	DHS	1,676	1,538	91.8	224.2
Mali	2018	DHS	2,855	2,732	95.7	1,187.5
Mauritania	2019–21	DHS	3,234	3,143	97.2	207.7
Niger	2012	DHS	3,373	3,252	96.4	1,188.6
Nigeria	2021	MICS	8,362	7,172	85.8	10,918.8
Sao Tome and Principe	2019	MICS	505	501	99.2	9.4
Senegal	2019	DHS	1,820	1,771	97.3	755.5
Sierra Leone	2019	DHS	2,868	2,648	92.3	358.3
Togo	2017	MICS	1,461	1,460	99.9	371.2

Notes: Full sample refers to the full sample of children 6–23 months old. Final sample refers to children remaining after excluding those with missing information on feeding from the full sample. Population refers to population of children 6–23 months old. Data sources are Demographic and Health Surveys (DHS) and Multiple Indicator Cluster Surveys (MICS).

© 2024 Karlsson O et al. *JAMA Network Open*.

eTable 2. Variable Labels for Questions on Complementary Feeding

Country	Year	Data	Wave*	Food variable label
Afghanistan	2015	DHS	7	Gave Child Fortified Baby Food (Cerelac, Etc)
-				Gave Child Bread, Noodles, Other Made From Grains
-				Gave Child Potatoes, Cassava, Or Other Tubers
-				Gave Child Eggs
-				Gave Child Meat (Beef, Pork, Lamb, Chicken, Etc)
-				Gave Child Pumpkin, Carrots, Squash (Yellow Or Orange Inside)
-				Gave Child Any Dark Green Leafy Vegetables
-				Gave Child Mangoes, Papayas, Other Vitamin A Fruits
-				Gave Child Any Other Fruits
-				Gave Child Liver, Heart, Other Organs
-				Gave Child Fish Or Shellfish
-				Gave Child Food Made From Beans, Peas, Lentils, Nuts
-				Gave Child Cheese, Yogurt, Other Milk Products
-				Gave Child Other Solid-Semisolid Food
-				Gave Child Yogurt
-				Gave Child Tinned, Powdered Or Fresh Milk
-				Gave Child Baby Formula
Albania	2017–18	DHS	7	Gave Child Fortified Baby Food (Cerelac, Etc)
-				Gave Child Bread, Noodles, Other Made From Grains
-				Gave Child Potatoes, Cassava, Or Other Tubers
-				Gave Child Eggs
-				Gave Child Meat (Beef, Pork, Lamb, Chicken, Etc)
-				Gave Child Pumpkin, Carrots, Squash (Yellow Or Orange Inside)
-				Gave Child Any Dark Green Leafy Vegetables
-				Gave Child Apricots, Peaches Or Cantaloupe, Other Vitamin A Fruits
-				Gave Child Any Other Fruits
-				Gave Child Liver, Heart, Other Organs
-				Gave Child Fish Or Shellfish
-				Gave Child Food Made From Beans, Peas, Lentils, Nuts
-				Gave Child Cheese, Yogurt, Other Milk Products
-				Gave Child Chocolates, Sweets, Candies, Pastries, Etc
-				Gave Child Other Solid-Semisolid Food
-				Gave Child Yogurt
-				Gave Child Tinned, Powdered Or Fresh Milk
-				Gave Child Baby Formula
Algeria	2018–19	MICS	6	Enfant A Bu Ou Mangé Du Yaourt Hier
-				Lenfant A Mangé Des Aliments Pour BéBéS Enrichis (Gerber, Hero, Cerelac, Nest
-				Lenfant A Mangé Des Aliments FabriquéS à Partir De CéRéAles Hier
-				Lenfant A Mangé Hier De La Citrouille, Des Carottes, Des Courges, Etc.
-				Lenfant A Mangé Hier Des Pommes De Terre Blanches, Des Ignames Blanches, Du Ma
-				Enfant A Mangé Des LéGumes à Feuilles Vertes Hier
-				Hier, Lenfant A Mangé Figues, Pommes, Poires,? (Fruits Locaux Riches En Vitami
-				Tout Autre Fruit Et LéGume, Comme Les Oranges, Les Bananes, Les Tomates, Les Ha
-				Lenfant A Mangé Du Foie, Des Reins, Du Coeur Ou Dautres Viandes Dorganes Hie
-				Lenfant A Mangé De La Viande, Comme Du Boeuf, De Lagneau, De La ChéVre, Du
-				Enfant A Mangé Des Oeufs Hier
-				Lenfant A Mangé Du Poisson Ou Des Fruits De Mer Frais Ou SéChéS Hier
-				Lenfant A Mangé Des Haricots, Des Pois, Des Lentilles Ou Des Noix Ou Toute Nou
-				Lenfant A Mangé Du Fromage Ou Dautres Aliments FabriquéS à Partir De Lait H
-				Lenfant A Mangé Dautres Aliments Solides, Semi-Solides Ou Mous Hier
-				Enfant A Bu Une PréParation Pour Nourrissons Hier
-				Enfant A Bu Du Lait Hier
Angola	2015–16	DHS	7	Gave Child Fortified Baby Food (Cerelac, Etc)
-				Gave Child Bread, Noodles, Other Made From Grains
-				Gave Child Potatoes, Cassava, Or Other Tubers
-				Gave Child Eggs
-				Gave Child Meat (Beef, Pork, Lamb, Chicken, Etc)
-				Gave Child Pumpkin, Carrots, Squash (Yellow Or Orange Inside)
-				Gave Child Any Dark Green Leafy Vegetables
-				Gave Child Mangoes, Papayas, Other Vitamin A Fruits
-				Gave Child Any Other Fruits
-				Gave Child Liver, Heart, Other Organs
-				Gave Child Fish Or Shellfish
-				Gave Child Food Made From Beans, Peas, Lentils, Nuts
-				Gave Child Cheese, Yogurt, Other Milk Products
-				Gave Child Oil, Fats, Butter, Products Made Of Them
-				Gave Child Other Solid-Semisolid Food
-				Gave Child Yogurt
-				Gave Child Tinned, Powdered Or Fresh Milk

© 2024 Karlsson O et al. *JAMA Network Open*.

Country	Year	Data	Wave*	Food variable label
-				Gave Child Baby Formula
Armenia	2015–16	DHS	7	Gave Child Fortified Baby Food (Cerelac, Etc)
-				Gave Child Bread, Noodles, Other Made From Grains
-				Gave Child Potatoes, Cassava, Or Other Tubers
-				Gave Child Eggs
-				Gave Child Meat (Beef, Pork, Lamb, Chicken, Etc)
-				Gave Child Pumpkin, Carrots, Squash (Yellow Or Orange Inside)
-				Gave Child Any Dark Green Leafy Vegetables
-				Gave Child Mangoes, Papayas, Other Vitamin A Fruits
-				Gave Child Any Other Fruits
-				Gave Child Liver, Heart, Other Organs
-				Gave Child Fish Or Shellfish
-				Gave Child Food Made From Beans, Peas, Lentils, Nuts
-				Gave Child Cheese, Yogurt, Other Milk Products
-				Gave Child Other Solid-Semisolid Food
-				Gave Child Yogurt
-				Gave Child Tinned, Powdered Or Fresh Milk
-				Gave Child Baby Formula
Bangladesh	2019	MICS	6	Child Ate Yogurt Yesterday
-				Child Ate Fortified Baby Food (Gerber, Hero, Cerelac, Nestum, Etc.) Yesterday
-				Child Ate Foods Made From Grains Yesterday
-				Child Ate Pumpkin, Carrots, Squash Etc. That Are Yellow Or Orange Inside Yesterd
-				Child Ate White Potatoes, White Yams, Manioc, Cassava Etc. Any Other Foods Made
-				Child Ate Green Leafy Vegetables Yesterday
-				Child Ate Ripe Mangoes, Papayas Etc. Any Other Vitamin A-Rich Fruits Yesterday
-				Child Ate Other Fruits Or Vegetables Yesterday
-				Child Ate Liver, Kidney, Heart Or Other Organ Meat Yesterday
-				Child Ate Meat, Such As Beef, Pork, Lamb, Goat, Chicken, Duck Yesterday
-				Child Ate Eggs Yesterday
-				Child Ate Fresh Or Dried Fish Or Shellfish Yesterday
-				Child Ate Beans, Peas, Lentils Or Nuts Or Any Food Made From These Yesterday
-				Child Ate Cheese Or Other Food Made From Milk Yesterday
-				Child Ate Other Solid, Semi-Solid Or Soft Food Yesterday
-				Child Drank Infant Formula Yesterday
-				Child Drank Milk From Animals Yesterday
Belarus	2019	MICS	6	Child Ate Yogurt Yesterday
-				Child Ate Fortified Baby Food (Gerber, Hero, Cerelac, Nestum, Etc.) Yesterday
-				Child Ate Foods Made From Grains Yesterday
-				Child Ate Pumpkin, Carrots, Squash Etc. That Are Yellow Or Orange Inside Yesterd
-				Child Ate White Potatoes, White Yams, Manioc, Cassava Etc. Any Other Foods Made
-				Child Ate Green Leafy Vegetables Yesterday
-				Child Ate Ripe Mangoes, Papayas Etc. Any Other Vitamin A-Rich Fruits Yesterday
-				Child Ate Other Fruits Or Vegetables Yesterday
-				Child Ate Liver, Kidney, Heart Or Other Organ Meat Yesterday
-				Child Ate Meat, Such As Beef, Pork, Lamb, Goat, Chicken, Duck Yesterday
-				Child Ate Eggs Yesterday
-				Child Ate Fresh Or Dried Fish Or Shellfish Yesterday
-				Child Ate Beans, Peas, Lentils Or Nuts Or Any Food Made From These Yesterday
-				Child Ate Cheese Or Other Food Made From Milk Yesterday
-				Child Ate Other Solid, Semi-Solid Or Soft Food Yesterday
-				Child Drank Infant Formula Yesterday
-				Child Drank Milk From Animals Yesterday
Belize	2015–16	MICS	5	Child Drank Or Ate Yogurt Yesterday
-				Child Ate Fortified Baby Food (Cerelac Etc.)
-				Child Ate Foods Made From Grains
-				Child Ate Pumpkin, Carrots, Squash Etc.
-				Child Ate White Potatoes, White Yams, Manioc Etc.
-				Child Ate Green Leafy Vegetables
-				Child Ate Ripe Mangoes, Papayas
-				Child Ate Other Fruits Or Vegetables
-				Child Ateliver, Kidney, Heart Or Other Organ Meat
-				Child Ate Meat, Such As Beef, Pork, Lamb, Goat, Chicken, Duck
-				Child Ate Eggs
-				Child Ate Fresh Or Dried Fish Or Shellfish
-				Child Ate Beans, Lentils Or Nuts
-				Child Ate Cheese Or Other Food Made From Milk
-				Child Ate Other Solid, Semi-Solid Or Soft Food Yesterday
-				Child Drank Milk Yesterday
-				Child Drank Infant Formula Yesterday
Benin	2017–18	DHS	7	Gave Child Fortified Baby Food (Cerelac, Etc)
-				Gave Child Bread, Noodles, Other Made From Grains

© 2024 Karlsson O et al. *JAMA Network Open*.

Country	Year	Data	Wave*	Food variable label
-				Gave Child Potatoes, Cassava, Or Other Tubers
-				Gave Child Eggs
-				Gave Child Meat (Beef, Pork, Lamb, Chicken, Etc)
-				Gave Child Pumpkin, Carrots, Squash (Yellow Or Orange Inside)
-				Gave Child Any Dark Green Leafy Vegetables
-				Gave Child Mangoes, Papayas, Other Vitamin A Fruits
-				Gave Child Any Other Fruits
-				Gave Child Liver, Heart, Other Organs
-				Gave Child Fish Or Shellfish
-				Gave Child Food Made From Beans, Peas, Lentils, Nuts
-				Gave Child Cheese, Yogurt, Other Milk Products
-				Gave Child Oil, Fats, Butter, Products Made Of Them
-				Gave Child Other Solid-Semisolid Food
-				Gave Child Cs Foods
-				Gave Child Yogurt
-				Gave Child Tinned, Powdered Or Fresh Milk
-				Gave Child Baby Formula
Burkina Faso	2010	DHS	6	Gave Child Bread, Noodles, Other Made From Grains
-				Gave Child Potatoes, Cassava, Or Other Tubers
-				Gave Child Eggs
-				Gave Child Meat (Beef, Pork, Lamb, Chicken, Etc)
-				Gave Child Pumpkin, Carrots, Squash (Yellow Or Orange Inside)
-				Gave Child Any Dark Green Leafy Vegetables
-				Gave Child Mangoes, Papayas, Other Vitamin A Fruits
-				Gave Child Any Other Fruits
-				Gave Child Liver, Heart, Other Organs
-				Gave Child Fish Or Shellfish
-				Gave Child Food Made From Beans, Peas, Lentils, Nuts
-				Gave Child Cheese, Yogurt, Other Milk Products
-				Gave Child Other Solid-Semisolid Food
-				Gave Child Yogurt
-				Gave Child Fortified Baby Food (Cerelac, Etc)
-				Gave Child Tinned, Powdered Or Fresh Milk
-				Gave Child Baby Formula
Burundi	2016–17	DHS	7	Gave Child Fortified Baby Food (Cerelac, Etc)
-				Gave Child Foods Prepared With Red Palm Oil
-				Gave Child Food Prepared With Refined Oil
-				Gave Child Sugar, Honey, Sugar Cane
-				Gave Child Commercial Biscuits Or Cakes
-				Gave Child Bread, Noodles, Other Made From Grains
-				Gave Child Potatoes, Cassava, Or Other Tubers
-				Gave Child Eggs
-				Gave Child Meat (Beef, Pork, Lamb, Chicken, Etc)
-				Gave Child Pumpkin, Carrots, Squash (Yellow Or Orange Inside)
-				Gave Child Any Dark Green Leafy Vegetables
-				Gave Child Mangoes, Papayas, Other Vitamin A Fruits
-				Gave Child Any Other Fruits
-				Gave Child Liver, Heart, Other Organs
-				Gave Child Fish Or Shellfish
-				Gave Child Food Made From Beans, Peas, Lentils, Nuts
-				Gave Child Cheese, Yogurt, Other Milk Products
-				Gave Child Other Solid-Semisolid Food
-				Gave Child Yogurt
-				Gave Child Tinned, Powdered Or Fresh Milk
-				Gave Child Baby Formula
Cambodia	2014	DHS	7	Gave Child Fortified Baby Food (Cerelac, Etc)
-				Na - Gave Child Yogurt
-				Gave Child Snake, Snail, Frog, Rat, Insects
-				Gave Child Bread, Noodles, Other Made From Grains
-				Gave Child Potatoes, Cassava, Or Other Tubers
-				Gave Child Eggs
-				Gave Child Meat (Beef, Pork, Lamb, Chicken, Etc)
-				Gave Child Pumpkin, Carrots, Squash (Yellow Or Orange Inside)
-				Gave Child Any Dark Green Leafy Vegetables
-				Gave Child Mangoes, Papayas, Other Vitamin A Fruits
-				Gave Child Any Other Fruits
-				Gave Child Liver, Heart, Other Organs
-				Gave Child Fish Or Shellfish
-				Gave Child Food Made From Beans, Peas, Lentils, Nuts
-				Gave Child Cheese, Yogurt, Other Milk Products
-				Gave Child Oil, Fats, Butter, Products Made Of Them

© 2024 Karlsson O et al. *JAMA Network Open*.

Country	Year	Data	Wave*	Food variable label
-				Gave Child Chocolates, Sweets, Candies, Pastries, Etc
-				Gave Child Other Solid-Semisolid Food
-				Gave Child Tinned, Powdered Or Fresh Milk
-				Gave Child Baby Formula
Cameroon	2018–19	DHS	7	Gave Child Fortified Baby Food (Cerelac, Etc)
-				Gave Child Bread, Noodles, Other Made From Grains
-				Gave Child Potatoes, Cassava, Or Other Tubers
-				Gave Child Eggs
-				Gave Child Meat (Beef, Pork, Lamb, Chicken, Etc)
-				Gave Child Pumpkin, Carrots, Squash (Yellow Or Orange Inside)
-				Gave Child Any Dark Green Leafy Vegetables
-				Gave Child Mangoes, Papayas, Other Vitamin A Fruits
-				Gave Child Any Other Fruits
-				Gave Child Liver, Heart, Other Organs
-				Gave Child Fish Or Shellfish
-				Gave Child Food Made From Beans, Peas, Lentils, Nuts
-				Gave Child Cheese, Yogurt, Other Milk Products
-				Gave Child Other Solid-Semisolid Food
-				Gave Child Foods Made With Red Palm Oil, Palm Nut, Or Palm Nut Pulp Sauce
-				Gave Grubs, Snails, Insects Or Other Small Protein Food
-				Gave Child Yogurt
-				Gave Child Tinned, Powdered Or Fresh Milk
-				Gave Child Baby Formula
Central African Republic	2018–19	MICS	6	Hier Lenfant A Bu Du Yaourt Fait Avec Du Lait D'Animal
-				A Mangé De La Nourriture Pour BéBé Telle Que Nursie, BléDine, Gallia, BléDi
-				Hier Lenfant A Mangé De La Nourriture à Base De CéRéAles
-				Hier Lenfant A Mangé Courge, Carotte, Citrouille, Etc
-				Hier Lenfant A Mangé La Nourriture à Base De Tubercules
-				Hier Lenfant A Mangé Les Legumes à Feuilles Vert Fonce
-				Hier Lenfant A Mangé Mangue Mure, Papaye Mure, Goyave, Avocat
-				Hier Lenfant A Mangé Autres Fruits Ou LéGumes
-				Hier, Lenfant A Mangé De La Viande De Foie, De Rein, De CœUr Ou Dautres Orga
-				Hier, Lenfant A Mangé Toute Autre Viande Comme Du BœUf, Du Porc, De L'Agnea
-				Hier, Lenfant A Mangé Des œUfs
-				Hier, Lenfant A Mangé Du Poisson Frais Ou Seche
-				Hier, Lenfant A Mangé Nourritures A Base De Haricots, Pois, Noix, Etc
-				Hier, Lenfant A Mangé Du Fromage Ou Autre Aliment Fait Avec Du Lait Animal
-				Hier Lenfant A Mangé Des Chenilles, Grenouille, Termites AilléEs (BôBô), Gr
-				Hier, Lenfant A Mangé Des Aliments ThéRapeutiques PrêT à L'Emploi Qu'On
-				Hier, Lenfant A Mangé Autre Aliment Solide, Semi-Solide Ou Mou
-				A Bu Du Lait Maternisé Tel Que Nursie, BléDine, Gallia, BléDilait, Nan,Guigoz
-				A Bu Du Lait D'Animal, Qu'Il Soit Frais, En BoîTe Ou En Poudre
Chad	2019	MICS	6	Lenfant A Bu Ou Mangé Des Yaourts Hier
-				Lenfant A Mangé De La Nourriture FortifiéE Pour BéBé (Cerelac, Gerber, Nest
-				Lenfant A Mangé Du Pain, Riz, PâTe, Bouillie Ou Autres Aliments Faits à Base
-				Lenfant A Mangé De La Courge, Carottes, Citrouille, Patate Douce, Etc. Qui Son
-				Lenfant A Mangé Des Pommes De Terre, De Ligname, Du Manioc Ou Autre Nourritur
-				Lenfant A Mangé Des LéGumes à Feuilles Vert Foncé
-				Lenfant A Mangé Des Mangues/Papayes Mures
-				Lenfant A Mangé Dautres Fruits Et LéGumes
-				Lenfant A Mangé Du Foie, Rognons, Coeur Ou Autres Abats
-				Lenfant A Mangé De La Viande De Boeuf, Porc, Agneau, ChèVre, Etc.
-				Lenfant A Mangé Des Oeufs
-				Lenfant A Mangé Du Poisson Frais Ou SéChé
-				Lenfant A Mangé Des Aliments à Base De Haricots, Pois, Etc.
-				Lenfant A Mangé Du Fromage Ou Autre Produit Laitier
-				Lenfant A Mangé Des Autres Produits Solides, Semi-Solides Ou Mous
-				Lenfant A Bu Une PréParation Pour BéBé Hier
-				Lenfant A Bu Du Lait Hier
Comoros	2012	DHS	6	Gave Child Fortified Baby Food (Cerelac, Etc)
-				Gave Child Bread, Noodles, Other Made From Grains
-				Gave Child Potatoes, Cassava, Or Other Tubers
-				Gave Child Eggs
-				Gave Child Meat (Beef, Pork, Lamb, Chicken, Etc)
-				Gave Child Pumpkin, Carrots, Squash (Yellow Or Orange Inside)
-				Gave Child Any Dark Green Leafy Vegetables
-				Gave Child Mangoes, Papayas, Other Vitamin A Fruits
-				Gave Child Any Other Fruits
-				Gave Child Liver, Heart, Other Organs
-				Gave Child Fish Or Shellfish
-				Gave Child Food Made From Beans, Peas, Lentils, Nuts

© 2024 Karlsson O et al. *JAMA Network Open*.

Country	Year	Data	Wave*	Food variable label
-				Gave Child Cheese, Yogurt, Other Milk Products
-				Gave Child Other Solid-Semisolid Food
-				Gave Child Yogurt
-				Gave Child Tinned, Powdered Or Fresh Milk
-				Gave Child Baby Formula
Congo	2014–15	MICS	5	Est-Ce Que (Nom) A Mange Yaourt Hier Durant Le Jour Ou La Nuit ?
-				Est-Ce-Que (Nom) A Mange Nâ€™Importe Quelle Marque De Nourriture Fortifiee
-				Est-Ce Que (Nom) A Mange Du Pain, Riz, Biscuits, Beignets, Pates, Porridge, Boui
-				Est-Ce Que (Nom) A Mange Courge, Carotte, Citrouille, Igname Jaune, Patate Douc
-				Est-Ce Que (Nom) A Mange Des Pommes De Terres, Des Ignames Blanches, Du Manioc,
-				Est-Ce Que (Nom) A Mange Nâ€™Importe Quels Legumes A Feuilles Vert Fonce C
-				Est-Ce Que (Nom) A Mange Des Mangues Mures/Papayes Mures ? (Fruits Locaux Riche
-				Est-Ce Que (Nom) A Mange Nâ€™Importe Quels Autres Fruits Ou Legumes Hier D
-				Est-Ce Que (Nom) A Mange Du Foie, Des Rognons, Du Cå'Ur Ou Tout Autre Abat H
-				Est-Ce Que (Nom) A Mange De La Viande De Bå'Uf, De Porc, Dâ€™Agneau, De
-				Est-Ce Que (Nom) A Mange Des Oeufs Hier Durant Le Jour Ou La Nuit ?
-				Est-Ce Que (Nom) A Mange Du Poisson Frais Ou Seche, Des Crevettes, Langoustes Ou
-				Est-Ce Que (Nom) A Mange Nâ€™Importe Quels Aliments A Base De Haricots, Poi
-				Est-Ce Que (Nom) A Mange Du Fromage Ou Tout Autre Produit A Base De Lait Autre Q
-				Est-Ce Que (Nom) A Mange Nâ€™Importe Quel Autre Aliment Solide, Semi-Solid
-				Est-Ce Que (Nom) A Mange Des Sauterelles, Grillons, Escargots, Termites, Chenill
-				Est-Ce Que (Nom) A Mange Preparation A Base Dâ€™Huile De Palme, De Sauce De
-				Est-Ce Que (Nom) A Bu Du Lait En Boite En Poudre Ou Liquide Hier, Durant Le Jour
-				Est-Ce Que (Nom) A Bu Une Preparation Pour Bebe Vendue En Commerce Hier, Durant
Congo DR	2017–18	MICS	6	Juste Pour êTre SûRe, Est-Ce Que (Nom) A Mangé Du : Yaourt
-				Juste Pour êTre SûRe, Est-Ce Que (Nom) A Mangé Du : De La Nourriture Pour Bé
-				Du Pain, Riz, PâTes, Porridge, Ou Autres Aliments à Base De Grains ?
-				Courge, Carotte, Citrouille, Patate Douce Qui Sont Jaune Ou Orange à L'IntéR
-				Des Pommes De Terre, De L'Igname, Du Manioc, De La Patate Douce Ou Autre Nourr
-				N'Importe Quel LéGume à Feuilles Vert Foncé Riche En Vit-A Comme Epinard, A
-				Mangues Mures Ou Des Papayes Mures Ou D'Autres Fruits Locaux Riches En Vit-A
-				Tout Autre Fruit Et LéGume, Parmi Les Plus CommunéMent ConsomméS Comme Avocat
-				Du Foie, Des Rognons, Du CœUr Ou Autres Abats ?
-				Toute Autre Viande Comme Du BœUf, Du Porc, De L'Agneau, De La ChèVre, Du Pou
-				Des Oeufs ?
-				Du Poisson Ou Des CrustacéS, Frais Ou SéChéS ?
-				Des Haricots, Petits Pois, Lentilles Ou Noix (Arachides, Amandes, Etc.), Y Compr
-				Du Fromage Ou Autre Aliment Fait Avec Du Lait D'Animal
-				Des Chenilles, Escargots, Termites
-				Autre Aliment Solide, Semi-Solide Ou Mou ?
-				Hier, Durant Le Jour Ou La Nuit, Est-Ce Que (Nom) A Bu : Lait Maternise
-				Hier, Durant Le Jour Ou La Nuit, Est-Ce Que (Nom) A Bu : Du Lait D'Animal, Qu
Costa Rica	2018	MICS	6	NiñO Comió Ayer Yogur
-				NiñO Comió Ayer Alimento Fortificado Para Bebé
-				NiñO Comió Ayer Alimentos Elaborados Con Granos
-				NiñO Comió Ayer Zanahoria, Ayote, Camote O Calabaza
-				NiñO Comió Ayer Alimentos Hechos A Base De RaíCes
-				NiñO Comió Ayer Verduras De Hojas Verde Oscuro Y Frondosas
-				NiñO Comió Ayer Mangos/Papaya Maduros
-				NiñO Comió Ayer Otras Frutas O Verduras
-				NiñO Comió Ayer VíSceras
-				NiñO Comió Ayer Otro Tipo De Carne
-				NiñO Comió Ayer Huevos
-				NiñO Comió Ayer Pescado O Mariscos Frescos O Congelados
-				NiñO Comió Ayer Alimentos A Base De Frijoles, Guisantes, Nueces, Etc.
-				NiñO Comió Ayer Queso U Otros Alimentos Hechos A Base De Leche De Origen Anima
-				NiñO Comió Ayer Gallo Pinto
-				NiñO Comió Ayer Otro Alimento SóLido, SemisóLido O Blando
-				NiñO Tomo Ayer Leche De FóRmula
-				NiñO Tomo Ayer Leche
Cote d'Ivoire	2016	MICS	5	Lenfant A Bu Ou Mangé Des Yaourts Hier
-				Lenfant A Mangé De La Nourriture FortifiéE Pour BéBé (Cerelac,Farinor,Bledi
-				Lenfant A Mangé Des Aliments Faits à Base De Grains
-				Lenfant A Mangé De La Courge, Carottes, Citrouille, Etc.
-				Lenfant A Mangé Des Pommes De Terre, Ignames, Manioc, Etc.
-				Lenfant A Mangé Des LéGumes à Feuilles Vert Foncé
-				Lenfant A Mangé Des Mangues/Papayes Mures
-				Lenfant A Mangé Dautres Fruits Et LéGumes
-				Lenfant A Mangé Du Foie, Rognons, Coeur Ou Autres Abats
-				Lenfant A Mangé De La Viande De Boeuf, Porc, Agneau, ChèVre, Etc.
-				Lenfant A Mangé Des Oeufs

© 2024 Karlsson O et al. *JAMA Network Open*.

Country	Year	Data	Wave*	Food variable label
-				Lenfant A Mangé Du Poisson Frais Ou SéChé
-				Lenfant A Mangé Des Aliments à Base De Haricots, Pois, Etc.
-				Lenfant A Mangé Du Fromage Ou Autre Produit Laitier
-				Lenfant A Mangé Des Autres Produits Solides, Semi-Solides Ou Mous
-				Lenfant A Mangé Des Escargots Ou Des Chenilles
-				Lenfant A Mangé De La Nourriture à Base Dhuile De Palme
-				Lenfant A Bu Du Lait Tel Que Du Lait En Boite, En Poudre Ou Du Lait Frais Hier
-				Lenfant A Bu Une PréParation Pour BéBé Hier
Cuba	2019	MICS	6	NiñO Comió Ayer Yogurt
-				NiñO Comió Ayer Alimento Fortificado Para Bebé
-				NiñO Comió Ayer Alimentos Elaborados Con Granos
-				NiñO Comió Ayer Calabaza, Zanahoria, Boniato, Etc.
-				NiñO Comió Ayer Alimentos Como Papa, Yuca, Malanga
-				NiñO Comió Ayer Verduras De Hojas Verde Oscuro Y Frondosas
-				NiñO Comió Ayer Mangos/Fruta Bomba Maduros
-				NiñO Comió Ayer Otras Frutas O Verduras
-				NiñO Comió Ayer VíSceras
-				NiñO Comió Ayer Otro Tipo De Carne
-				NiñO Comió Ayer Huevos
-				Pescado O Mariscos Ya Sean Frescos O Secos
-				NiñO Comió Ayer Alimentos A Base De Frijoles, Chicharos, Lentejas, Etc.
-				NiñO Comió Ayer Queso U Otros Alimentos Hechos A Base De Leche De Origen Anima
-				NiñO Comió Ayer Otro Alimento SóLido, SemisóLido O Blando
-				NiñO Tomo Ayer Leche De FóRmula
-				NiñO Tomo Ayer Leche
Dominican Republic	2019	MICS	6	¿Yogurt Hecho De Leche De Origen Animal?
-				¿AlgúN Alimento Para Bebé, Como Cereales,Papillas,Harinas Del Negrito,Maicena
-				¿Pan, Arroz, Pastas U Otros Alimentos Hechos Con Granos?
-				¿Auyama, CalabacíN, Zanahorias,Batata Dulces Que Son De Color Amarillo O Naran
-				¿Papa, Yuca, ñAme Blanco, YautíA O Cualquier Alimento Elaborado A Partir De L
-				¿Alguna Verdura De Hojas Verde Oscuro Y Frondosas Como Espinacas,BróColi,Perej
-				¿Mangos Maduros,Lechosas,Melones,SandíAs,Etc.?
-				¿Alguna Otra Fruta O Verdura,Como Manzanas,Naranjas,Uvas,Aguacate,Mandarinas,Et
-				¿HíGado,RiñóN,CorazóN U Otros óRganos De Animales?
-				¿Cualquier Otro Tipo De Carne, Como Carne De Res, Cerdo, Chivo, Ovejo, Pollo, P
-				¿Huevos?
-				¿Pescado O Mariscos, Ya Sean Frescos O Secos?
-				¿Habichuelas, Guandules, Lentejas, Garbanzos, Soya O Nueces, Incluyendo Cualqui
-				¿Queso U Otros Alimentos Hechos A Base De Leche?
-				¿Cualquier Otro Alimento SóLido, SemisóLido O Blando Que No Haya Mencionado?
-				¿Leche De FóRmula, Como Enfamil, Milex, Alacta, Nido, Etc.?
-				¿Bebió Leche De Origen Animal, Como Leche Fresca, Envasada O En Polvo?
Egypt	2014	DHS	6	Gave Child Fortified Baby Food (Cerelac, Etc)
-				Gave Child Bread, Noodles, Other Made From Grains
-				Gave Child Potatoes, Cassava, Or Other Tubers
-				Gave Child Eggs
-				Gave Child Meat (Beef, Pork, Lamb, Chicken, Etc)
-				Gave Child Pumpkin, Carrots, Squash (Yellow Or Orange Inside)
-				Gave Child Any Dark Green Leafy Vegetables
-				Gave Child Mangoes, Papayas, Other Vitamin A Fruits
-				Gave Child Any Other Fruits
-				Gave Child Liver, Heart, Other Organs
-				Gave Child Fish Or Shellfish
-				Gave Child Food Made From Beans, Peas, Lentils, Nuts
-				Gave Child Cheese, Yogurt, Other Milk Products
-				Gave Child Other Solid-Semisolid Food
-				Gave Child Yogurt
-				Gave Child Tinned, Powdered Or Fresh Milk
-				Gave Child Baby Formula
El Salvador	2014	MICS	5	NiñO Tomó O Comió Yogurt Ayer
-				NiñO Comió Alimento Fortificado Para Bebé
-				NiñO Comió Alimento Hecho De Granos
-				NiñO Comió Calabaza, Zanahoria, Patatas, Etc.
-				NiñO Comió Papas Blancas, ñAme Blanco, Yuca, Etc.
-				NiñO Comió Verdura De Hoja Verde
-				NiñO Comió Mango Maduro, Papayas.
-				NiñO Comió Otras Frutas O Verduras
-				NiñO Comió HíGado, RiñóN, CorazóN U Otros óRganos De Carne
-				NiñO Comió Carne, Res, Cerdo, Cordero U Otra Carne
-				NiñO Comió Huevos
-				NiñO Comió Pescado O Marisco Fresco

© 2024 Karlsson O et al. *JAMA Network Open*.

Country	Year	Data	Wave*	Food variable label
-				NiñO Comió Frijoles, Lentejas O Nueces
-				NiñO Comió Queso U Otra Clase De Comida Hecha De Leche
-				NiñO Comió Alimento Solido, Semi-Solido O Blando No Mencionado
-				NiñO Tomó Leche Ayer
-				NiñO Tomó Leche De FóRmula Para Bebe
Eswatini	2014	MICS	5	Child Drank Or Ate Yogurt Yesterday
-				Child Ate Fortified Baby Food (Cerelac, Nestam Etc.)
-				Child Ate Foods Made From Grains
-				Child Ate Pumpkin, Carrots, Squash Etc.
-				Child Ate White Potatoes, White Yams, Manioc Etc.
-				Child Ate Green Leafy Vegetables
-				Child Ate Ripe Mangoes, Papayas
-				Child Ate Other Fruits Or Vegetables
-				Child Ate Liver, Kidney, Heart Or Other Organ Meat
-				Child Ate Meat, Such As Beef, Pork, Lamb, Goat, Chicken, Duck
-				Child Ate Eggs
-				Child Ate Fresh Or Dried Fish Or Shellfish
-				Child Ate Beans, Lentils Or Nuts
-				Child Ate Cheese Or Other Food Made From Milk
-				Child Ate Other Solid, Semi-Solid Or Soft Food Yesterday
-				Child Drank Milk Yesterday
-				Child Drank Infant Formula Yesterday
Ethiopia	2019	DHS	8	Gave Child Fortified Baby Food (Cerelac, Etc)
-				Gave Child Bread, Noodles, Other Made From Grains
-				Gave Child Potatoes, Cassava, Or Other Tubers
-				Gave Child Eggs
-				Gave Child Meat (Beef, Pork, Lamb, Chicken, Etc)
-				Gave Child Pumpkin, Carrots, Squash (Yellow Or Orange Inside)
-				Gave Child Any Dark Green Leafy Vegetables
-				Gave Child Mangoes, Papayas, Other Vitamin A Fruits
-				Gave Child Any Other Fruits
-				Gave Child Liver, Heart, Other Organs
-				Gave Child Fish Or Shellfish
-				Gave Child Food Made From Beans, Peas, Lentils, Nuts
-				Gave Child Cheese, Yogurt, Other Milk Products
-				Gave Child Other Solid-Semisolid Food
-				Gave Child Yogurt
-				Gave Child Tinned, Powdered Or Fresh Milk
-				Gave Child Baby Formula
Fiji	2021	MICS	6	Child Ate Yogurt Yesterday
-				Child Ate Fortified Baby Food (Gerber, Hero, Cerelac, Nestum, Etc.) Yesterday
-				Child Ate Foods Made From Grains Yesterday
-				Child Ate Pumpkin, Carrots, Squash Etc. That Are Yellow Or Orange Inside Yesterd
-				Child Ate White Potatoes, White Yams, Manioc, Cassava Etc. Any Other Foods Made
-				Child Ate Green Leafy Vegetables Yesterday
-				Child Ate Ripe Mangoes, Papayas Etc. Any Other Vitamin A-Rich Fruits Yesterday
-				Child Ate Other Fruits Or Vegetables Yesterday
-				Child Ate Liver, Kidney, Heart Or Other Organ Meat Yesterday
-				Child Ate Meat, Such As Beef, Pork, Lamb, Goat, Chicken, Duck Yesterday
-				Child Ate Eggs Yesterday
-				Child Ate Fresh Or Dried Fish Or Shellfish Yesterday
-				Child Ate Beans, Peas, Lentils Or Nuts Or Any Food Made From These Yesterday
-				Child Ate Cheese Or Other Food Made From Milk Yesterday
-				Child Ate Vegetable Pakora Yesterday
-				Child Ate Other Solid, Semi-Solid Or Soft Food Yesterday
-				Child Drank Infant Formula Yesterday
-				Child Drank Milk From Animals Yesterday
Gabon	2012	DHS	6	Gave Child Fortified Baby Food (Cerelac, Etc)
-				Gave Child Bread, Noodles, Other Made From Grains
-				Gave Child Potatoes, Cassava, Or Other Tubers
-				Gave Child Eggs
-				Gave Child Meat (Beef, Pork, Lamb, Chicken, Etc)
-				Gave Child Pumpkin, Carrots, Squash (Yellow Or Orange Inside)
-				Gave Child Any Dark Green Leafy Vegetables
-				Gave Child Mangoes, Papayas, Other Vitamin A Fruits
-				Gave Child Any Other Fruits
-				Gave Child Liver, Heart, Other Organs
-				Gave Child Fish Or Shellfish
-				Gave Child Food Made From Beans, Peas, Lentils, Nuts
-				Gave Child Cheese, Yogurt, Other Milk Products
-				Gave Child Other Solid-Semisolid Food

© 2024 Karlsson O et al. *JAMA Network Open*.

Country	Year	Data	Wave*	Food variable label
-				Gave Child Yogurt
-				Gave Child Tinned, Powdered Or Fresh Milk
-				Gave Child Baby Formula
Gambia	2019–20	DHS	8	Gave Child Fortified Baby Food (Cerelac, Etc)
-				Gave Child Bread, Noodles, Other Made From Grains
-				Gave Child Potatoes, Cassava, Or Other Tubers
-				Gave Child Eggs
-				Gave Child Meat (Beef, Pork, Lamb, Chicken, Etc)
-				Gave Child Pumpkin, Carrots, Squash (Yellow Or Orange Inside)
-				Gave Child Any Dark Green Leafy Vegetables
-				Gave Child Mangoes, Papayas, Other Vitamin A Fruits
-				Gave Child Any Other Fruits
-				Gave Child Liver, Heart, Other Organs
-				Gave Child Fish Or Shellfish
-				Gave Child Food Made From Beans, Peas, Lentils, Nuts
-				Gave Child Cheese, Yogurt, Other Milk Products
-				Gave Child Other Solid-Semisolid Food
-				Gave Child Foods Made With Red Palm Oil, Palm Nut, Or Palm Nut Pulp Sauce
-				Gave Child Yogurt
-				Gave Child Tinned, Powdered Or Fresh Milk
-				Gave Child Baby Formula
Georgia	2018	MICS	6	Child Ate Yogurt Yesterday
-				Child Ate Fortified Baby Food (Gerber, Hero, Cerelac, Nestum, Etc.) Yesterday
-				Child Ate Foods Made From Grains Yesterday
-				Child Ate Pumpkin, Carrots, Squash Etc. That Are Yellow Or Orange Inside Yesterd
-				Child Ate White Potatoes, White Yams, Manioc, Cassava Etc. Any Other Foods Made
-				Child Ate Green Leafy Vegetables Yesterday
-				Child Ate Ripe Mangoes, Papayas Etc. Any Other Vitamin A-Rich Fruits Yesterday
-				Child Ate Other Fruits Or Vegetables Yesterday
-				Child Ate Liver, Kidney, Heart Or Other Organ Meat Yesterday
-				Child Ate Meat, Such As Beef, Pork, Lamb, Goat, Chicken, Duck Yesterday
-				Child Ate Eggs Yesterday
-				Child Ate Fresh Or Dried Fish Or Shellfish Yesterday
-				Child Ate Beans, Peas, Lentils Or Nuts Or Any Food Made From These Yesterday
-				Child Ate Cheese Or Other Food Made From Milk Yesterday
-				Child Ate Nuts Yesterday
-				Child Ate Other Solid, Semi-Solid Or Soft Food Yesterday
-				Child Drank Infant Formula, Such As Humana, Hipp, Nestle, Similac, Etc Yesterday
-				Child Drank Milk From Animals Yesterday
-				Child Drank Clear Tea/Tea Made Without Milk /Dairy Products Yesterday
-				Child Drank Cocoa Made Without Milk /Dairy Products Yesterday
-				Child Drank Cocoa Made With Milk Yesterday
Ghana	2017–18	MICS	6	Child Drank Or Ate Yogurt Yesterday
-				Child Ate Fortified Baby Food (Gerber, Hero, Cerelac, Nestum, Etc.) Yesterday
-				Child Ate Any Fortified Baby Food Such As Weanimix Yesterday
-				Child Ate Foods Made From Grains Yesterday
-				Child Ate Pumpkin, Carrots, Squash Etc. Yesterday
-				Child Ate White Potatoes, White Yams, Manioc, Cassava Etc. Yesterday
-				Child Ate Green Leafy Vegetables Yesterday
-				Child Ate Ripe Mangoes, Pawpaw Yesterday
-				Child Ate Other Fruits Or Vegetables Yesterday
-				Child Ate Liver, Kidney, Heart Or Other Organ Meat Yesterday
-				Child Ate Meat, Such As Beef, Pork, Lamb, Goat, Chicken, Duck Yesterday
-				Child Ate Insects Such As Termites, Ceickwts, Caterpillars Etc Yesterday
-				Child Ate Eggs Yesterday
-				Child Ate Fresh Or Dried Fish Or Shellfish Yesterday
-				Child Ate Beans, Peas, Lentils Or Nuts Or Any Food Made From These Yesterday
-				Child Ate Cheese Or Other Food Made From Milk Yesterday
-				Child Ate Sugary Food Such As Chocolate, Sweet , ...Yesterday
-				Child Ate Food Made From Or With Red Palm Oil , ...Yesterday
-				Child Ate Other Solid, Semi-Solid Or Soft Food Yesterday
-				Child Drank Infant Formula Yesterday
-				Child Drank Milk Yesterday
Guatemala	2014–15	DHS	7	Gave Child Fortified Baby Food (Cerelac, Etc)
-				Gave Child Other Porridge/Gruel
-				Gave Child Foods Made With Corn, Tortillas, Tamales
-				Gave Child Bread, Noodles, Other Made From Grains
-				Gave Child Potatoes, Cassava, Or Other Tubers
-				Gave Child Eggs
-				Gave Child Meat (Beef, Pork, Lamb, Chicken, Etc)
-				Gave Child Pumpkin, Carrots, Squash (Yellow Or Orange Inside)

© 2024 Karlsson O et al. *JAMA Network Open*.

Country	Year	Data	Wave*	Food variable label
-				Gave Child Any Dark Green Leafy Vegetables
-				Gave Child Mangoes, Papayas, Other Vitamin A Fruits
-				Gave Child Any Other Fruits
-				Gave Child Liver, Heart, Other Organs
-				Gave Child Fish Or Shellfish
-				Gave Child Food Made From Beans, Peas, Lentils, Nuts
-				Gave Child Cheese, Yogurt, Other Milk Products
-				Gave Child Oil, Fats, Butter, Products Made Of Them
-				Gave Child Chocolates, Sweets, Candies, Pastries, Etc
-				Gave Child Other Solid-Semisolid Food
-				Gave Child Sugar, Honey
-				Gave Child Yogurt
-				Gave Child Tinned, Powdered Or Fresh Milk
-				Gave Child Baby Formula
Guinea	2018	DHS	7	Gave Child Fortified Baby Food (Cerelac, Etc)
-				Gave Child Bread, Noodles, Other Made From Grains
-				Gave Child Potatoes, Cassava, Or Other Tubers
-				Gave Child Eggs
-				Gave Child Meat (Beef, Pork, Lamb, Chicken, Etc)
-				Gave Child Pumpkin, Carrots, Squash (Yellow Or Orange Inside)
-				Gave Child Any Dark Green Leafy Vegetables
-				Gave Child Mangoes, Papayas, Other Vitamin A Fruits
-				Gave Child Any Other Fruits
-				Gave Child Liver, Heart, Other Organs
-				Gave Child Fish Or Shellfish
-				Gave Child Food Made From Beans, Peas, Lentils, Nuts
-				Gave Child Cheese, Yogurt, Other Milk Products
-				Gave Child Other Solid-Semisolid Food
-				Gave Child Yogurt
-				Gave Child Tinned, Powdered Or Fresh Milk
-				Gave Child Baby Formula
Guinea-Bissau	2018–19	MICS	6	CriançA Bebeu Ou Comeu Iogurte Ontem
-				A CriançA Comeu Comida De Bebê Fortificada (Gerber, HerólI, Cerelac, Nestum, E
-				CriançA Comeu Alimentos Feitos A Partir De GrãOs Ontem
-				CriançA Comeu AbóBora, Cenoura, AbóBora Etc. Ontem
-				CriançA Comeu Batata, Inhame, Matabala, Fruta-PãO, Mandioca Ou Outro Alimento
-				A CriançA Comeu Verduras De Folhas Verdes Ontem
-				CriançA Comeu Mangas Maduras, Mamao Ontem
-				CriançA Comeu Outras Frutas Ou Legumes Ontem
-				CriançA Comeu FíGado, Rim, CoraçãO Ou Outra Carne De óRgãO Ontem
-				A CriançA Comeu Carne, Como Carne De Vaca, Carne De Porco, Cordeiro, Cabra, Gal
-				CriançA Comeu Ovos Ontem
-				CriançA Comeu Peixe Fresco Ou Secado Ou Molusco Ontem
-				CriançA Comeu FeijõEs, Ervilhas, Lentilhas Ou Nozes Ou Qualquer Comida Feita A
-				Uma CriançA Comeu Queijo Ou Outra Comida Feita De Leite Ontem
-				CriançA Comeu Outra Comida SóLida, Semi-SóLida Ou Macia Ontem
-				CriançA Bebeu Leite De Pacoteou Leite Em PóInfantil, P.Ex. Nan, Aptamil, Nutri
-				CriançA Bebeu Leite Animal Ontem
Guyana	2014	MICS	5	Child Ate Any Nestum
-				Child Ate Bread, Puri, Float Bake, Barah, Samosas, Rice, Noodles, Chow Mein Or Ot
-				Child Ate Pumpkin, Carrots, Squash, Sweet Potatoes Etc.
-				Child Ate White Potatoes, White Yams, Manioc Etc.
-				Child Ate Green Leafy Vegetables
-				Child Ate Ripe Mangoes, Papayas
-				Child Ate Other Fruits Or Vegetables
-				Child Ateliver, Kidney, Heart Or Other Organ Meat
-				Child Ate Meat, Such As Beef, Pork, Lamb, Goat, Chicken, Duck
-				Child Ate Eggs
-				Child Ate Fresh Or Dried Fish, Etc
-				Child Ate Beans, Lentils Or Nuts Coconuts/Coconut Milk
-				Child Ate Cheese Or Other Food Made From Milk
-				Child Ate Other Solid, Semi-Solid Or Soft Food Yesterday
-				Child Drank Milk Yesterday
-				Child Drank Infant Formula Yesterday
Haiti	2016–17	DHS	7	Gave Child Fortified Baby Food (Cerelac, Etc)
-				Gave Child Bread, Noodles, Other Made From Grains
-				Gave Child Potatoes, Cassava, Or Other Tubers
-				Gave Child Eggs
-				Gave Child Meat (Beef, Pork, Lamb, Chicken, Etc)
-				Gave Child Pumpkin, Carrots, Squash (Yellow Or Orange Inside)
-				Gave Child Any Dark Green Leafy Vegetables

© 2024 Karlsson O et al. *JAMA Network Open*.

Country	Year	Data	Wave*	Food variable label
-				Gave Child Mangoes, Papayas, Other Vitamin A Fruits
-				Gave Child Any Other Fruits
-				Gave Child Liver, Heart, Other Organs
-				Gave Child Fish Or Shellfish
-				Gave Child Food Made From Beans, Peas, Lentils, Nuts
-				Gave Child Cheese, Yogurt, Other Milk Products
-				Gave Child Other Solid-Semisolid Food
-				Gave Child Yogurt
-				Gave Child Tinned, Powdered Or Fresh Milk
-				Gave Child Baby Formula
Honduras	2019	MICS	6	NiñO Comió Ayer Yogurt
-				NiñO Comió Ayer Alimento Fortificado Para Bebé
-				NiñO Comió Ayer Alimentos Elaborados Con Granos
-				NiñO Comió Ayer Calabaza, Zanahoria, Ayote, Camote, Etc.
-				NiñO Comió Ayer Alimentos Hechos A Base De RaíCes
-				NiñO Comió Ayer Verduras De Hojas Verde Oscuro Y Frondosas
-				NiñO Comió Ayer Mangos/Papaya Maduros
-				NiñO Comió Ayer Otras Frutas O Verduras
-				NiñO Comió Ayer VíSceras
-				NiñO Comió Ayer Otro Tipo De Carne
-				NiñO Comió Ayer Huevos
-				NiñO Comió Ayer Pescado Fresco O Seco
-				NiñO Comió Ayer Alimentos A Base De Frijoles, Guisantes, Nueces, Lentejas. Etc
-				NiñO Comió Ayer Queso U Otros Alimentos Hechos A Base De Leche De Origen Anima
-				NiñO Comió Ayer Otro Alimento SóLido, SemisóLido O Blando
-				NiñO Tomo Ayer Leche De FóRmula
-				NiñO Tomo Ayer Leche De Origen Animal, Como Leche Fresca, Envasada ..?
India	2019–21	DHS	7	Gave Child Fortified Baby Food (Cerelac, Etc)
-				Gave Child Any Chicken, Duck Or Other Birds
-				Gave Child Bread, Noodles, Other Made From Grains
-				Gave Child Potatoes, Cassava, Or Other Tubers
-				Gave Child Eggs
-				Gave Child Pumpkin, Carrots, Squash (Yellow Or Orange Inside)
-				Gave Child Any Dark Green Leafy Vegetables
-				Gave Child Mangoes, Papayas, Other Vitamin A Fruits
-				Gave Child Any Other Fruits
-				Gave Child Liver, Heart, Other Organs
-				Gave Child Fish Or Shellfish
-				Gave Child Food Made From Beans, Peas, Lentils, Nuts
-				Gave Child Cheese, Yogurt, Other Milk Products
-				Gave Child Other Solid-Semisolid Food
-				Gave Child Other Meat
-				Gave Child Yogurt
-				Gave Child Tinned, Powdered Or Fresh Milk
-				Gave Child Baby Formula
Indonesia	2017	DHS	7	Gave Child Fortified Baby Food (Cerelac, Etc)
-				Gave Child Bread, Noodles, Other Made From Grains
-				Gave Child Potatoes, Cassava, Or Other Tubers
-				Gave Child Eggs
-				Gave Child Meat (Beef, Pork, Lamb, Chicken, Etc)
-				Gave Child Pumpkin, Carrots, Squash (Yellow Or Orange Inside)
-				Gave Child Any Dark Green Leafy Vegetables
-				Gave Child Mangoes, Papayas, Other Vitamin A Fruits
-				Gave Child Any Other Fruits
-				Gave Child Liver, Heart, Other Organs
-				Gave Child Fish Or Shellfish
-				Gave Child Food Made From Beans, Peas, Lentils, Nuts
-				Gave Child Cheese, Yogurt, Other Milk Products
-				Gave Child Other Solid-Semisolid Food
-				Gave Child Yogurt
-				Gave Child Tinned, Powdered Or Fresh Milk
-				Gave Child Baby Formula
Iraq	2018	MICS	6	Child Drank Or Ate Yogurt Yesterday
-				Child Ate Fortified Baby Food (Gerber, Hero, Cerelac, Nestum, Etc.) Yesterday
-				Child Ate Foods Made From Grains Yesterday
-				Child Ate Pumpkin, Carrots, Squash Etc. Yesterday
-				Child Ate White Potatoes, White Yams, Manioc, Cassava Etc. Yesterday
-				Child Ate Green Leafy Vegetables Yesterday
-				Child Ate Ripe Mangoes, Papayas Yesterday
-				Child Ate Other Fruits Or Vegetables Yesterday
-				Child Ate Liver, Kidney, Heart Or Other Organ Meat Yesterday

© 2024 Karlsson O et al. *JAMA Network Open*.

Country	Year	Data	Wave*	Food variable label
-				Child Ate Meat, Such As Beef, Pork, Lamb, Goat, Chicken, Duck Yesterday
-				Child Ate Eggs Yesterday
-				Child Ate Fresh Or Dried Fish Or Shellfish Yesterday
-				Child Ate Beans, Peas, Lentils Or Nuts Or Any Food Made From These Yesterday
-				Child Ate Cheese Or Other Food Made From Milk Yesterday
-				Child Ate Other Solid, Semi-Solid Or Soft Food Yesterday
-				Child Drank Infant Formula Yesterday
-				Child Drank Milk Yesterday
Jordan	2017–18	DHS	7	Gave Child Fortified Baby Food (Cerelac, Etc)
-				Gave Child Bread, Noodles, Other Made From Grains
-				Gave Child Potatoes, Cassava, Or Other Tubers
-				Gave Child Eggs
-				Gave Child Meat (Beef, Pork, Lamb, Chicken, Etc)
-				Gave Child Pumpkin, Carrots, Squash (Yellow Or Orange Inside)
-				Gave Child Any Dark Green Leafy Vegetables
-				Gave Child Mangoes, Papayas, Other Vitamin A Fruits
-				Gave Child Any Other Fruits
-				Gave Child Liver, Heart, Other Organs
-				Gave Child Fish Or Shellfish
-				Gave Child Food Made From Beans, Peas, Lentils, Nuts
-				Gave Child Cheese, Yogurt, Other Milk Products
-				Gave Child Other Solid/Semi-Solid Food
-				Gave Child Yogurt
-				Gave Child Tinned, Powdered Or Fresh Milk
-				Gave Child Baby Formula
Kazakhstan	2015	MICS	5	Child Drank Or Ate Yogurt, Kefir, Ayran Or Qatiq
-				Child Ate Fortified Baby Food (Gerber, Etc.)
-				Child Ate Foods Made From Grains
-				Child Ate Pumpkin Or Carrots
-				Child Ate Any Food Made From Potatoes, Or Made From Roots
-				Child Ate Green Leafy Vegetables
-				Child Ate Dried Apricots Or Ripe Persimmon
-				Child Ate Other Fruits Or Vegetables
-				Child Ate Liver, Kidney, Heart Or Other Organ Meat
-				Child Ate Meat, Such As Beef, Pork, Lamb, Goat, Chicken, Duck
-				Child Ate Eggs
-				Child Ate Fresh Or Dried Fish
-				Child Ate Beans, Lentils Or Nuts
-				Child Ate Cheese Or Other Food Made From Milk
-				Child Ate Other Solid, Semi-Solid Or Soft Food Yesterday
-				Child Ate Sugary Foods Such As Chocolate, Sweets, Candies, Cookies, Cakes Or Bis
-				Child Ate Any Fried, Salty Snacks Such As Potato Chips
-				Child Drank Milk Yesterday
-				Child Drank Infant Formula Yesterday
Kenya	2014	DHS	7	Gave Child Fortified Baby Food (Cerelac, Etc)
-				Gave Child Bread, Noodles, Other Made From Grains
-				Gave Child Potatoes, Cassava, Or Other Tubers
-				Gave Child Eggs
-				Gave Child Meat (Beef, Pork, Lamb, Chicken, Etc)
-				Gave Child Pumpkin, Carrots, Squash (Yellow Or Orange Inside)
-				Gave Child Any Dark Green Leafy Vegetables
-				Gave Child Mangoes, Papayas, Other Vitamin A Fruits
-				Gave Child Any Other Fruits
-				Gave Child Liver, Heart, Other Organs
-				Gave Child Fish Or Shellfish
-				Gave Child Food Made From Beans, Peas, Lentils, Nuts
-				Gave Child Cheese, Yogurt, Other Milk Products
-				Gave Child Other Solid-Semisolid Food
-				Gave Child Yogurt
-				Gave Child Tinned, Powdered Or Fresh Milk
-				Gave Child Baby Formula
Kiribati	2018–19	MICS	6	Child Ate Yogurt Yesterday
-				Child Ate Fortified Baby Food Such Heinz Yesterday
-				Child Ate Foods Made From Grains Yesterday
-				Child Ate Pumpkin, Carrots, Squash Etc. That Are Yellow Or Orange Inside Yesterday
-				Child Ate White Potatoes, White Yams, Manioc, Cassava Etc. Any Other Foods Made
-				Child Ate Green Leafy Vegetables Yesterday
-				Child Ate Ripe Mangoes, Papayas Etc. Any Other Vitamin A-Rich Fruits Yesterday
-				Child Ate Other Fruits Or Vegetables Yesterday
-				Child Ate Liver, Kidney, Heart Or Other Organ Meat Yesterday
-				Child Ate Meat, Such As Beef, Pork, Lamb, Goat, Chicken, Duck Yesterday

© 2024 Karlsson O et al. *JAMA Network Open*.

Country	Year	Data	Wave*	Food variable label
-				Child Ate Eggs Yesterday
-				Child Ate Fresh Or Dried Fish Or Shellfish Yesterday
-				Child Ate Beans, Peas, Lentils Or Nuts Or Any Food Made From These Yesterday
-				Child Ate Cheese Or Other Food Made From Milk Yesterday
-				Child Ate Other Solid, Semi-Solid Or Soft Food Yesterday
-				Child Drank Infant Formula Yesterday
-				Child Drank Milk From Animals Yesterday
Kosovo	2019–20	MICS	6	Child Ate Yogurt Yesterday
-				Child Ate Fortified Baby Food Made Of Grains Yesterday
-				Child Ate Foods Made From Grains Yesterday
-				Child Ate Pumpkin, Carrots, Squash Etc. That Are Yellow Or Orange Inside Yesterd
-				Child Ate White Potatoes Or Any Other Foods Made From Roots Yesterday
-				Child Ate Green Leafy Vegetables Yesterday
-				Child Ate Sour Cherries, Apricots Etc Or Any Other Vitamin A-Rich Fruits Yesterd
-				Child Ate Other Fruits Or Vegetables Yesterday
-				Child Ate Liver, Kidney, Heart Or Other Organ Meat Yesterday
-				Child Ate Meat, Such As Beef, Pork, Lamb, Goat, Chicken, Duck Yesterday
-				Child Ate Eggs Yesterday
-				Child Ate Fresh Or Dried Fish Or Shellfish Yesterday
-				Child Ate Beans, Peas, Lentils Or Nuts Or Any Food Made From These Yesterday
-				Child Ate Cheese Or Other Food Made From Milk Yesterday
-				Child Ate Other Solid, Semi-Solid Or Soft Food Yesterday
-				Child Drank Infant Formula Yesterday
-				Child Drank Milk From Animals Yesterday
Kyrgyzstan	2018	MICS	6	Child Ate Yogurt Yesterday
-				Child Ate Fortified Baby Food (Gerber, Hero, Cerelac, Nestum, Etc.) Yesterday
-				Child Ate Foods Made From Grains Yesterday
-				Child Ate Pumpkin, Carrots, Squash Etc. That Are Yellow Or Orange Inside Yesterd
-				Child Ate White Potatoes, White Yams, Manioc, Cassava Etc. Any Other Foods Made
-				Child Ate Green Leafy Vegetables Yesterday
-				Child Ate Ripe Mangoes, Papayas Etc. Any Other Vitamin A-Rich Fruits Yesterday
-				Child Ate Other Fruits Or Vegetables Yesterday
-				Child Ate Liver, Kidney, Heart Or Other Organ Meat Yesterday
-				Child Ate Meat, Such As Beef, Pork, Lamb, Goat, Chicken, Duck Yesterday
-				Child Ate Eggs Yesterday
-				Child Ate Fresh Or Dried Fish Or Shellfish Yesterday
-				Child Ate Beans, Peas, Lentils Or Nuts Or Any Food Made From These Yesterday
-				Child Ate Cheese Or Other Food Made From Milk Yesterday
-				Child Ate Other Solid, Semi-Solid Or Soft Food Yesterday
-				Child Drank Infant Formula Yesterday
-				Child Drank Milk From Animals Yesterday
Lao	2017	MICS	6	Child Ate Yogurt Made From Animal Milk Yesterday
-				Child Ate Fortified Baby Food (Cerelac, Nestum, Pediasure) Yesterday
-				Child Ate Bread, Rice, Noodles, Porridge, Or Other Foods Made From Grains Yester
-				Child Ate Pumpkin, Carrots, Squash Or Sweet Potatoes That Are Yellow Or Orange I
-				Child Ate White Potatoes, White Yams, Cassava Or Other Foods Made From Roots Yes
-				Child Ate Any Dark Green, Leafy Vegetables, Such As Spinach, Morning Glory, Sala
-				Child Ate Ripe Mangoes, Ripe Papayas, Or Other Locally Available Vitamin A-Rich
-				Child Ate Any Other Fruits Or Vegetables Such As Watermelon, Banana Yesterday
-				Child Ate Liver, Kidney, Heart Or Other Organ Meats Yesterday
-				Child Ate Any Other Meat, Such As Beef, Pork, Lamb, Goat, Chicken, Duck Or Sausa
-				Child Ate Eggs Yesterday
-				Child Ate Fresh Or Dried Fish Or Shellfish Yesterday
-				Child Ate Beans, Peas, Lentils Or Nuts Or Any Food Made From These Yesterday
-				Child Ate Cheese Or Other Food Made From Animal Milk Yesterday
-				Child Ate Other Solid, Semi-Solid Or Soft Food Yesterday
-				Child Drank Infant Formula (Cerelac, Pediasure) Yesterday
-				Child Drank Milk From Animals (Fresh, Tinned, Or Powdered) Yesterday
Lesotho	2018	MICS	6	Child Ate Yogurt Made From Animal Milk Yesterday
-				Child Ate Any Baby Food Yesterday
-				Child Ate Bread, Rice, Noodles, Porridge Or Other Foods Made From Grains Yesterday
-				Child Ate Pumpkin, Butternut, Carrots Or Squash That Are Yellow Or Orange Yesterday
-				Child Ate White Potatoes, Radish Or Other Foods Made From Roots Yesterday
-				Child Ate Any Dark Green, Leafy Vegetables Yesterday
-				Child Ate Ripe Mangoes, Apricots Or Papayas Yesterday
-				Child Ate Any Other Fruits Or Vegetables Yesterday
-				Child Ate Liver, Kidney, Heart Or Other Organ Meats Yesterday
-				Child Ate Any Other Meat Yesterday
-				Child Ate Eggs Yesterday
-				Child Ate Fresh Or Dried Fish Or Shellfish Yesterday
-				Child Ate Beans, Peas, Lentils Or Nuts, Including Any Foods Made From These Yest

© 2024 Karlsson O et al. *JAMA Network Open*.

Country	Year	Data	Wave*	Food variable label
-				Child Ate Cheese Or Other Food Made From Animal Milk Yesterday
-				Child Ate Other Solid, Semi-Solid Or Soft Food Yesterday
-				Child Drank Infant Formula Yesterday
-				Child Drank Milk From Animals Yesterday
Liberia	2019–20	DHS	7	Gave Child Fortified Baby Food (Cerelac, Etc)
-				Gave Child Bread, Noodles, Other Made From Grains
-				Gave Child Potatoes, Cassava, Or Other Tubers
-				Gave Child Eggs
-				Gave Child Meat (Beef, Pork, Lamb, Chicken, Etc)
-				Gave Child Pumpkin, Carrots, Squash (Yellow Or Orange Inside)
-				Gave Child Any Dark Green Leafy Vegetables
-				Gave Child Mangoes, Papayas, Other Vitamin A Fruits
-				Gave Child Any Other Fruits
-				Gave Child Liver, Heart, Other Organs
-				Gave Child Fish Or Shellfish
-				Gave Child Food Made From Beans, Peas, Lentils, Nuts
-				Gave Child Cheese, Yogurt, Other Milk Products
-				Gave Child Oil, Fats, Butter, Products Made Of Them
-				Gave Child Other Solid-Semisolid Food
-				Gave Child Yogurt
-				Gave Child Tinned, Powdered Or Fresh Milk
-				Gave Child Baby Formula - Guigoz
Madagascar	2021	DHS	8	Gave Child Fortified Baby Food (Cerelac, Etc)
-				Gave Child Bread, Noodles, Other Made From Grains
-				Gave Child Potatoes, Cassava, Or Other Tubers
-				Gave Child Eggs
-				Gave Child Meat (Beef, Pork, Lamb, Chicken, Etc)
-				Gave Child Pumpkin, Carrots, Squash (Yellow Or Orange Inside)
-				Gave Child Any Dark Green Leafy Vegetables
-				Gave Child Mangoes, Papayas, Other Vitamin A Fruits
-				Gave Child Any Other Fruits
-				Gave Child Liver, Heart, Other Organs
-				Gave Child Fish Or Shellfish
-				Gave Child Food Made From Beans, Peas, Lentils, Nuts
-				Gave Child Cheese, Yogurt, Other Milk Products
-				Gave Child Other Solid-Semisolid Food
-				Gave Child Yogurt
-				Gave Child Tinned, Powdered Or Fresh Milk
-				Gave Child Baby Formula
Malawi	2019–20	MICS	6	Child Ate Yogurt Yesterday
-				Child Ate Fortified Baby Food (Gerber, Hero, Cerelac, Nestum, Etc.) Yesterday
-				Child Ate Foods Made From Grains Yesterday
-				Child Ate Pumpkin, Carrots, Squash Etc. That Are Yellow Or Orange Inside Yesterd
-				Child Ate White Potatoes, White Yams, Manioc, Cassava Etc. Any Other Foods Made
-				Child Ate Green Leafy Vegetables Yesterday
-				Child Ate Ripe Mangoes, Papayas Etc. Any Other Vitamin A-Rich Fruits Yesterday
-				Child Ate Other Fruits Or Vegetables Yesterday
-				Child Ate Liver, Kidney, Heart Or Other Organ Meat Yesterday
-				Child Ate Meat, Such As Beef, Pork, Lamb, Goat, Chicken, Duck Yesterday
-				Child Ate Eggs Yesterday
-				Child Ate Fresh Or Dried Fish Or Shellfish Yesterday
-				Child Ate Beans, Peas, Lentils Or Nuts Or Any Food Made From These Yesterday
-				Child Ate Cheese Or Other Food Made From Milk Yesterday
-				Child Ate Other Solid, Semi-Solid Or Soft Food Yesterday
-				Child Drank Infant Formula Yesterday
-				Child Drank Milk From Animals Yesterday
Maldives	2016–17	DHS	7	Gave Child Fortified Baby Food (Cerelac, Etc)
-				Gave Child Bread, Noodles, Other Made From Grains
-				Gave Child Potatoes, Cassava, Or Other Tubers
-				Gave Child Eggs
-				Gave Child Meat (Beef, Pork, Lamb, Chicken, Etc)
-				Gave Child Pumpkin, Carrots, Squash (Yellow Or Orange Inside)
-				Gave Child Any Dark Green Leafy Vegetables
-				Gave Child Mangoes, Papayas, Other Vitamin A Fruits
-				Gave Child Any Other Fruits
-				Gave Child Liver, Heart, Other Organs
-				Gave Child Fish Or Shellfish
-				Gave Child Food Made From Beans, Peas, Lentils, Nuts
-				Gave Child Cheese, Yogurt, Other Milk Products
-				Gave Child Other Solid-Semisolid Food
-				Gave Child Yogurt

© 2024 Karlsson O et al. *JAMA Network Open*.

Country	Year	Data	Wave*	Food variable label
-				Gave Child Tinned, Powdered Or Fresh Milk
-				Gave Child Baby Formula
Mali	2018	DHS	7	Gave Child Fortified Baby Food (Cerelac, Etc)
-				Gave Child Bread, Noodles, Other Made From Grains
-				Gave Child Potatoes, Cassava, Or Other Tubers
-				Gave Child Eggs
-				Gave Child Meat (Beef, Pork, Lamb, Chicken, Etc)
-				Gave Child Pumpkin, Carrots, Squash (Yellow Or Orange Inside)
-				Gave Child Any Dark Green Leafy Vegetables
-				Gave Child Mangoes, Papayas, Other Vitamin A Fruits
-				Gave Child Any Other Fruits
-				Gave Child Liver, Heart, Other Organs
-				Gave Child Fish Or Shellfish
-				Gave Child Food Made From Beans, Peas, Lentils, Nuts
-				Gave Child Cheese, Yogurt, Other Milk Products
-				Gave Child Other Solid-Semisolid Food
-				Gave Child Caterpillars, Firifirini Or Other Types Of Food Containing Protein
-				Gave Child A Preparation Based On Red Palm Oil, Palm Nuts Or Walnut Pulp Sauce
-				Gave Child Yogurt
-				Gave Child Tinned, Powdered Or Fresh Milk
-				Gave Child Baby Formula
Mauritania	2019–21	DHS	7	Gave Child Fortified Baby Food (Cerelac, Etc)
-				Gave Child Bread, Noodles, Other Made From Grains
-				Gave Child Potatoes, Cassava, Or Other Tubers
-				Gave Child Eggs
-				Gave Child Meat (Beef, Pork, Lamb, Chicken, Etc)
-				Gave Child Pumpkin, Carrots, Squash (Yellow Or Orange Inside)
-				Gave Child Any Dark Green Leafy Vegetables
-				Gave Child Mangoes, Papayas, Other Vitamin A Fruits
-				Gave Child Any Other Fruits
-				Gave Child Liver, Heart, Other Organs
-				Gave Child Fish Or Shellfish
-				Gave Child Food Made From Beans, Peas, Lentils, Nuts
-				Gave Child Cheese, Yogurt, Other Milk Products
-				Gave Child Other Solid-Semisolid Food
-				Gave Child Escargots
-				Gave Child Yogurt
-				Gave Child Tinned, Powdered Or Fresh Milk
-				Gave Child Baby Formula
Mexico	2015	MICS	5	Yogurt
-				Alimento Fortificado Para Bebe
-				Alimentos Elaborados Con Granos/Pan/Arroz
-				Calabaza, Zanahoria, Camote, Etc
-				Papas Blancas, Camote Blanco, Tuberculos
-				Verduras De Hojas Verde Obscuro
-				Mangos Maduros, Papaya, Melon
-				Otra Fruta O Verdura
-				Higado, RiñOn, CorazóN, Etc
-				Carne De Res, Cerdo, Borrego, Etc.
-				Huevos
-				Pescado O Mariscos Frescos O Secos
-				Frijoles,Chicharos, Lentejas O Nueces
-				Otros Alimentos Hechos Con Leche, Excepto Yogurt
-				Otro Alimento SóLido, Semi-Solido O Blando
-				El NiñO Comio Azucar Chocolates, Pan Dulce
-				El NiñO Comio Comida Salada O Botanas Como Papitas
-				Bebio Leche Envasada
-				Leche De Formula Para Bebes
Mongolia	2018	MICS	6	Child Ate Yogurt Yesterday
-				Child Ate Fortified Baby Food (Gerber, Hero, Cerelac, Nestum, Etc.) Yesterday
-				Child Ate Foods Made From Grains Yesterday
-				Child Ate Pumpkin, Carrots, Squash Etc. That Are Yellow Or Orange Inside Yesterd
-				Child Ate White Potatoes, White Yams, Manioc, Cassava Etc. Any Other Foods Made
-				Child Ate Green Leafy Vegetables Yesterday
-				Child Ate Ripe Mangoes, Papayas Etc. Any Other Vitamin A-Rich Fruits Yesterday
-				Child Ate Other Fruits Or Vegetables Yesterday
-				Child Ate Liver, Kidney, Heart Or Other Organ Meat Yesterday
-				Child Ate Meat, Such As Beef, Pork, Lamb, Goat, Chicken, Duck Yesterday
-				Child Ate Eggs Yesterday
-				Child Ate Fresh Or Dried Fish Or Shellfish Yesterday
-				Child Ate Beans, Peas, Lentils Or Nuts Or Any Food Made From These Yesterday

© 2024 Karlsson O et al. *JAMA Network Open*.

Country	Year	Data	Wave*	Food variable label
-				Child Ate Cheese Or Other Food Made From Milk Yesterday
-				Child Ate Other Solid, Semi-Solid Or Soft Food Yesterday
-				Child Drank Infant Formula Yesterday
-				Child Drank Milk From Animals Yesterday
Montenegro	2018	MICS	6	Child Ate Yoghurt Yesterday
-				Child Ate Fortified Baby Food (Hipp, Nestle, Frutek, Juvitana, Etc.) Yesterday
-				Child Ate Bread, Rice, Pasta, Semolina/Polenta Or Other Foods Made From Grains Y
-				Child Ate Pumpkin, Carrots, Squash Or Sweet Potatoes That Are Yellow Or Orange I
-				Child Ate Potatoes Or Any Other Food Made From Roots Which Are White Inside Yest
-				Child Ate Any Dark Green, Leafy Vegetables Such As Spinach Or Swiss Chard Yester
-				Child Ate Apricots, Sour Cherries, Ripe Melons Or Any Other Vitamin A-Rich Fruit
-				Child Ate Other Fruits Or Vegetables Yesterday
-				Child Ate Liver, Heart, Kidney Or Other Organ Meat Yesterday
-				Child Ate Meat, Such As Beef, Pork, Lamb, Goat, Chicken Or Duck Yesterday
-				Child Ate Eggs Yesterday
-				Child Ate Fresh Or Dried Fish Or Shellfish Yesterday
-				Child Ate Beans, Peas, Lentils Or Nuts Or Any Food Made From These Yesterday
-				Child Ate Cheese Or Other Food Made From Milk Yesterday
-				Child Ate Other Solid, Semi-Solid Or Soft Food Yesterday
-				Child Drank Infant Formula Yesterday
-				Child Drank Milk From Animals Yesterday
Mozambique	2011	DHS	6	Gave Child Bread, Noodles, Other Made From Grains
-				Gave Child Potatoes, Cassava, Or Other Tubers
-				Gave Child Eggs
-				Gave Child Meat (Beef, Pork, Lamb, Chicken, Etc)
-				Gave Child Pumpkin, Carrots, Squash (Yellow Or Orange Inside)
-				Gave Child Any Dark Green Leafy Vegetables
-				Gave Child Mangoes, Papayas, Other Vitamin A Fruits
-				Gave Child Any Other Fruits
-				Gave Child Liver, Heart, Other Organs
-				Gave Child Fish Or Shellfish
-				Gave Child Food Made From Beans, Peas, Lentils, Nuts
-				Gave Child Cheese, Yogurt, Other Milk Products
-				Gave Child Oil, Fats, Butter, Products Made Of Them
-				Gave Child Other Solid-Semisolid Food
-				Gave Child Yogurt
-				Gave Child Fortified Baby Food (Cerelac, Etc)
-				Gave Child Tinned, Powdered Or Fresh Milk
-				Gave Child Baby Formula
Myanmar	2015–16	DHS	7	Gave Child Fortified Baby Food (Cerelac, Etc)
-				Gave Child Bread, Noodles, Other Made From Grains
-				Gave Child Potatoes, Cassava, Or Other Tubers
-				Gave Child Eggs
-				Gave Child Meat (Beef, Pork, Lamb, Chicken, Etc)
-				Gave Child Pumpkin, Carrots, Squash (Yellow Or Orange Inside)
-				Gave Child Any Dark Green Leafy Vegetables
-				Gave Child Mangoes, Papayas, Other Vitamin A Fruits
-				Gave Child Any Other Fruits
-				Gave Child Liver, Heart, Other Organs
-				Gave Child Fish Or Shellfish
-				Gave Child Food Made From Beans, Peas, Lentils, Nuts
-				Gave Child Cheese, Yogurt, Other Milk Products
-				Gave Child Other Solid-Semisolid Food
-				Gave Child Yogurt
-				Gave Child Tinned, Powdered Or Fresh Milk
-				Gave Child Baby Formula
Namibia	2013	DHS	6	Gave Child Fortified Baby Food (Cerelac, Etc)
-				Gave Child Bread, Noodles, Other Made From Grains
-				Gave Child Potatoes, Cassava, Or Other Tubers
-				Gave Child Eggs
-				Gave Child Meat (Beef, Pork, Lamb, Chicken, Etc)
-				Gave Child Pumpkin, Carrots, Squash (Yellow Or Orange Inside)
-				Gave Child Any Dark Green Leafy Vegetables
-				Gave Child Mangoes, Papayas, Other Vitamin A Fruits
-				Gave Child Any Other Fruits
-				Gave Child Liver, Heart, Other Organs
-				Gave Child Fish Or Shellfish
-				Gave Child Food Made From Beans, Peas, Lentils, Nuts
-				Gave Child Cheese, Yogurt, Other Milk Products
-				Gave Child Other Solid-Semisolid Food
-				Gave Child Yogurt

© 2024 Karlsson O et al. *JAMA Network Open*.

Country	Year	Data	Wave*	Food variable label
-				Gave Child Tinned, Powdered Or Fresh Milk
-				Gave Child Baby Formula
Nepal	2019	MICS	6	Child Ate Yogurt Yesterday
-				Child Ate Fortified Baby Food Cerelac, Nestum, Champion Etc. Yesterday
-				Child Ate Foods Made From Grains Yesterday
-				Child Ate Pumpkin, Carrots, Squash Etc. That Are Yellow Or Orange Inside Yesterday
-				Child Ate White Potatoes, White Yams, Manioc, Cassava Etc. Any Other Foods Made
-				Child Ate Green Leafy Vegetables Yesterday
-				Child Ate Ripe Mangoes, Papayas, Apricots Etc. Any Other Vitamin A-Rich Fruits Y
-				Child Ate Other Fruits Or Vegetables Such As Banana, Orange, Grapes Etc. Yesterday
-				Child Ate Liver, Kidney, Heart Or Other Organ Meat Yesterday
-				Child Ate Meat, As Beef, Lamb, Goat, Chicken, Duck Etc. Or Sausages Made From T
-				Child Ate Eggs Yesterday
-				Child Ate Fresh Or Dried Fish Or Shellfish Yesterday
-				Child Ate Beans, Peas, Lentils Or Nuts Or Any Food Made From These Yesterday
-				Child Ate Cheese Or Other Food Made From Milk Yesterday
-				Child Ate Other Solid, Semi-Solid Or Soft Food Yesterday
-				Child Drank Infant Formula Yesterday
-				Child Drank Milk From Animals Yesterday
Niger	2012	DHS	6	Gave Child Fortified Baby Food (Cerelac, Etc)
-				Gave Child Bread, Noodles, Other Made From Grains
-				Gave Child Potatoes, Cassava, Or Other Tubers
-				Gave Child Eggs
-				Gave Child Meat (Beef, Pork, Lamb, Chicken, Etc)
-				Gave Child Pumpkin, Carrots, Squash (Yellow Or Orange Inside)
-				Gave Child Any Dark Green Leafy Vegetables
-				Gave Child Mangoes, Papayas, Other Vitamin A Fruits
-				Gave Child Any Other Fruits
-				Gave Child Liver, Heart, Other Organs
-				Gave Child Fish Or Shellfish
-				Gave Child Food Made From Beans, Peas, Lentils, Nuts
-				Gave Child Cheese, Yogurt, Other Milk Products
-				Gave Child Other Solid-Semisolid Food
-				Gave Child Yogurt
-				Gave Child Tinned, Powdered Or Fresh Milk
-				Gave Child Baby Formula
Nigeria	2021	MICS	6	Child Ate Yogurt Yesterday
-				Child Ate Fortified Baby Food (Gerber, Hero, Cerelac, Nestum, Etc.) Yesterday
-				Child Ate Foods Made From Grains Yesterday
-				Child Ate Pumpkin, Carrots, Squash Etc. That Are Yellow Or Orange Inside Yesterday
-				Child Ate White Potatoes, White Yams, Manioc, Cassava Etc. Any Other Foods Made
-				Child Ate Green Leafy Vegetables Yesterday
-				Child Ate Ripe Mangoes, Papayas Etc. Any Other Vitamin A-Rich Fruits Yesterday
-				Child Ate Other Fruits Or Vegetables Yesterday
-				Child Ate Liver, Kidney, Heart Or Other Organ Meat Yesterday
-				Child Ate Meat, Such As Beef, Pork, Lamb, Goat, Chicken, Duck Yesterday
-				Child Ate Eggs Yesterday
-				Child Ate Fresh Or Dried Fish Or Shellfish Yesterday
-				Child Ate Beans, Peas, Lentils Or Nuts Or Any Food Made From These Yesterday
-				Child Ate Cheese Or Other Food Made From Milk Yesterday
-				Child Ate Other Solid, Semi-Solid Or Soft Food Yesterday
-				Child Drank Infant Formula Yesterday
-				Child Drank Milk From Animals Yesterday
North Macedonia	2018–19	MICS	6	Child Ate Yogurt Yesterday
-				Child Ate Fortified Baby Food (Gerber, Hero, Cerelac, Nestum, Etc.) Yesterday
-				Child Ate Foods Made From Grains Yesterday
-				Child Ate Pumpkin, Carrots, Squash Etc. That Are Yellow Or Orange Inside Yesterday
-				Child Ate White Potatoes, White Yams, Manioc, Cassava Etc. Any Other Foods Made
-				Child Ate Green Leafy Vegetables Yesterday
-				Child Ate Ripe Mangoes, Papayas Etc. Any Other Vitamin A-Rich Fruits Yesterday
-				Child Ate Other Fruits Or Vegetables Yesterday
-				Child Ate Liver, Kidney, Heart Or Other Organ Meat Yesterday
-				Child Ate Meat, Such As Beef, Pork, Lamb, Goat, Chicken, Duck Yesterday
-				Child Ate Eggs Yesterday
-				Child Ate Fresh Or Dried Fish Or Shellfish Yesterday
-				Child Ate Beans, Peas, Lentils Or Nuts Or Any Food Made From These Yesterday
-				Child Ate Cheese Or Other Food Made From Milk Yesterday
-				Child Ate Other Solid, Semi-Solid Or Soft Food Yesterday
-				Child Drank Infant Formula Yesterday
-				Child Drank Milk From Animals Yesterday
Pakistan	2017–18	DHS	7	Gave Child Fortified Baby Food (Cerelac, Etc)

© 2024 Karlsson O et al. *JAMA Network Open*.

Country	Year	Data	Wave*	Food variable label
-				Gave Child Bread, Noodles, Other Made From Grains
-				Gave Child Potatoes, Cassava, Or Other Tubers
-				Gave Child Eggs
-				Gave Child Meat (Beef, Pork, Lamb, Chicken, Etc)
-				Gave Child Pumpkin, Carrots, Squash (Yellow Or Orange Inside)
-				Gave Child Any Dark Green Leafy Vegetables
-				Gave Child Mangoes, Papayas, Other Vitamin A Fruits
-				Gave Child Any Other Fruits
-				Gave Child Liver, Heart, Other Organs
-				Gave Child Fish Or Shellfish
-				Gave Child Food Made From Beans, Peas, Lentils, Nuts
-				Gave Child Cheese, Yogurt, Other Milk Products
-				Gave Child Other Solid-Semisolid Food
-				Gave Child Yogurt
-				Gave Child Tinned, Powdered Or Fresh Milk
-				Gave Child Baby Formula
Papua New Guinea	2016–18	DHS	7	Gave Child Bread, Noodles, Other Made From Grains
-				Gave Child Potatoes, Cassava, Or Other Tubers
-				Gave Child Eggs
-				Gave Child Meat (Beef, Pork, Lamb, Chicken, Etc)
-				Gave Child Pumpkin, Carrots, Squash (Yellow Or Orange Inside)
-				Gave Child Any Dark Green Leafy Vegetables
-				Gave Child Mangoes, Papayas, Other Vitamin A Fruits
-				Gave Child Any Other Fruits
-				Gave Child Liver, Heart, Other Organs
-				Gave Child Fish Or Shellfish
-				Gave Child Food Made From Beans, Peas, Lentils, Nuts
-				Gave Child Cheese, Yogurt, Other Milk Products
-				Gave Child Other Solid-Semisolid Food
-				Gave Child Tinned, Powdered Or Fresh Milk
-				Gave Child Baby Formula
Paraguay	2016	MICS	5	Comió/Bebió Yogurt
-				Alimento Fortificado Para Bebé
-				Alimentos Elaborados Con Granos/Pan/Arroz
-				Zapallos, Zanahoria, Zapallitos, Etc
-				Papas Blancas, Mandioca, Etc
-				Verduras De Hojas Verdes, Oscuro Y Frondosas
-				Mango Maduro, MamóN,MelóN,Durazno, Etc
-				Otra Fruta O Verdura,Aguacate,Naranja,Mandarina,Manzana,Etc
-				Higado, RiñóN, CorazóN, Etc
-				Carne Vacuna, Cerdo, Oveja, Cabra, Etc
-				Huevos
-				Pescado Frescos O Congelados
-				Porotos, Guisantes, Lentejas, Nueces Etc.
-				Queso U Otros Alimentos Hechos Con Leche
-				Otro Alimento SóLido, Semi-SóLido O Blando
-				Bebió Leche En Sachet, En Carton, En Polvo O Leche Fresca
-				Bebió Leche De FóRmula Para BebéS
Peru	2012	DHS	6	Gave Child Oranges, Tangerine, Grapefruit, Lime
-				Gave Child Porridge From Social Programs
-				Gave Child Dried Fruits
-				Gave Child Bread, Noodles, Other Made From Grains
-				Gave Child Potatoes, Cassave, Or Other Tubers
-				Gave Child Eggs
-				Gave Child Meat (Beef, Pork, Lamb, Chicken, Etc)
-				Gave Child Pumpkin, Carrots, Squash (Yellow Or Orange Inside)
-				Gave Child Any Dark Green Leafy Vegetables
-				Gave Child Mangoes, Papayas, Other Vitamin A Fruits
-				Gave Child Any Other Fruits
-				Gave Child Food Made From Beans, Peas, Lentils, Nuts
-				Gave Child Cheese, Yogurt , Other Milk Products
-				Gave Child Oil, Fats, Butter, Products Made Of Them
-				Gave Child Chocolates, Sweets, Candies, Pastries, Etc
-				Gave Child Other Solid-Semisolid Food
-				Gave Child Tinned/Powder Or Fresh Milk
-				Gave Child Baby Formula
-				Gave Child Fresh Milk
Rwanda	2019–20	DHS	8	Gave Child Fortified Baby Food (Cerelac, Etc)
-				Gave Child Bread, Noodles, Other Made From Grains
-				Gave Child Potatoes, Cassava, Or Other Tubers
-				Gave Child Eggs

© 2024 Karlsson O et al. *JAMA Network Open*.

Country	Year	Data	Wave*	Food variable label
-				Gave Child Meat (Beef, Pork, Lamb, Chicken, Etc)
-				Gave Child Pumpkin, Carrots, Squash (Yellow Or Orange Inside)
-				Gave Child Any Dark Green Leafy Vegetables
-				Gave Child Mangoes, Papayas, Other Vitamin A Fruits
-				Gave Child Any Other Fruits
-				Gave Child Liver, Heart, Other Organs
-				Gave Child Fish Or Shellfish
-				Gave Child Food Made From Beans, Peas, Lentils, Nuts
-				Gave Child Cheese, Yogurt, Other Milk Products
-				Gave Child Other Solid-Semisolid Food
-				Gave Child Yogurt
-				Gave Child Tinned, Powdered Or Fresh Milk
-				Gave Child Baby Formula
Samoa	2019–20	MICS	6	Child Ate Yogurt Yesterday
-				Child Ate Fortified Baby Food (Cerelac, Gerber, Hero Or Nestum, Etc.) Yesterday
-				Child Ate Foods Made From Grains Yesterday
-				Child Ate Pumpkin, Carrots, Squash Etc. That Are Yellow Or Orange Inside Yesterday
-				Child Ate White Potatoes, White Yams, Manioc, Cassava Etc. Any Other Foods Made
-				Child Ate Green Leafy Vegetables Yesterday
-				Child Ate Ripe Mangoes, Papayas Etc. Any Other Vitamin A-Rich Fruits Yesterday
-				Child Ate Other Fruits Or Vegetables Yesterday
-				Child Ate Liver, Kidney, Heart Or Other Organ Meat Yesterday
-				Child Ate Meat, Such As Beef, Pork, Lamb, Goat, Chicken, Duck Yesterday
-				Child Ate Eggs Yesterday
-				Child Ate Fresh Or Dried Fish Or Shellfish Yesterday
-				Child Ate Beans, Peas, Lentils Or Nuts Or Any Food Made From These Yesterday
-				Child Ate Cheese Or Other Food Made From Milk Yesterday
-				Child Ate Other Solid, Semi-Solid Or Soft Food Yesterday
-				Child Drank Infant Formula Yesterday
-				Child Drank Milk From Animals Yesterday
Sao Tome and Principe	2019	MICS	6	CriançA Bebeu Ou Comeu Iogurte Ontem
-				A CriançA Comeu Comida De Bebê Fortificada (Gerber, HeróI, Cerelac, Nestum, E
-				CriançA Comeu Alimentos Feitos A Partir De GrãOs Ontem
-				CriançA Comeu AbóBora, Cenoura, AbóBora Etc. Ontem
-				CriançA Comeu Batata, Inhame, Matabala, Fruta-PãO, Mandioca Ou Outro Alimento
-				A CriançA Comeu Verduras De Folhas Verdes Ontem
-				CriançA Comeu Mangas Maduras, Mamao Ontem
-				CriançA Comeu Outras Frutas Ou Legumes Ontem
-				CriançA Comeu FíGado, Rim, CoraçãO Ou Outra Carne De óRgãO Ontem
-				A CriançA Comeu Carne, Como Carne De Vaca, Carne De Porco, Cordeiro, Cabra, Gal
-				CriançA Comeu Ovos Ontem
-				CriançA Comeu Peixe Fresco Ou Secado Ou Molusco Ontem
-				CriançA Comeu FeijõEs, Ervilhas, Lentilhas Ou Nozes Ou Qualquer Comida Feita A
-				Uma CriançA Comeu Queijo Ou Outra Comida Feita De Leite Ontem
-				CriançA Comeu Outra Comida SóLida, Semi-SóLida Ou Macia Ontem
-				CriançA Bebeu Leite De Pacoteou Leite Em PóInfantil, P.Ex. Nan, Aptamil, Nutri
-				CriançA Bebeu Leite Animal Ontem
Senegal	2019	DHS	8	Gave Child Fortified Baby Food (Cerelac, Etc)
-				Gave Child Sweet Snacks
-				Gave Child Salty Snacks
-				Gave Child Bread, Noodles, Other Made From Grains
-				Gave Child Potatoes, Cassava, Or Other Tubers
-				Gave Child Eggs
-				Gave Child Meat (Beef, Pork, Lamb, Chicken, Etc)
-				Gave Child Pumpkin, Carrots, Squash (Yellow Or Orange Inside)
-				Gave Child Any Dark Green Leafy Vegetables
-				Gave Child Mangoes, Papayas, Other Vitamin A Fruits
-				Gave Child Any Other Fruits
-				Gave Child Liver, Heart, Other Organs
-				Gave Child Fish Or Shellfish
-				Gave Child Food Made From Beans, Peas, Lentils, Nuts
-				Gave Child Cheese, Yogurt, Other Milk Products
-				Gave Child Other Solid-Semisolid Food
-				Gave Child Yogurt
-				Gave Child Tinned, Powdered Or Fresh Milk
-				Gave Child Baby Formula
Serbia	2019	MICS	6	Child Ate Yogurt Yesterday
-				Child Ate Fortified Baby Food (Gerber, Hero, Cerelac, Nestum, Etc.) Yesterday
-				Child Ate Foods Made From Grains Yesterday
-				Child Ate Pumpkin, Carrots, Squash Etc. That Are Yellow Or Orange Inside Yesterday
-				Child Ate White Potatoes, White Yams, Manioc, Cassava Etc. Any Other Foods Made

© 2024 Karlsson O et al. *JAMA Network Open*.

Country	Year	Data	Wave*	Food variable label
-				Child Ate Green Leafy Vegetables Yesterday
-				Child Ate Fresh Or Dried Apricots, Ripe Cantaloupe Etc. Any Other Vitamin A-Rich
-				Child Ate Other Fruits Or Vegetables Yesterday
-				Child Ate Liver, Kidney, Heart Or Other Organ Meat Yesterday
-				Child Ate Meat, Such As Veel/Young Beef, Pork, Lamb, Goat, Chicken, Duck Yesterday
-				Child Ate Eggs Yesterday
-				Child Ate Fresh Or Dried Fish Or Shellfish Yesterday
-				Child Ate Beans, Peas, Lentils Or Nuts Or Any Food Made From These Yesterday
-				Child Ate Cheese Or Other Food Made From Milk Yesterday
-				Child Ate Other Solid, Semi-Solid Or Soft Food Yesterday
-				Child Drank Infant Formula Yesterday
-				Child Drank Milk From Animals Yesterday
Sierra Leone	2019	DHS	7	Gave Child Fortified Baby Food (Cerelac, Etc)
-				Gave Child Bread, Noodles, Other Made From Grains
-				Gave Child Potatoes, Cassava, Or Other Tubers
-				Gave Child Eggs
-				Gave Child Meat (Beef, Pork, Lamb, Chicken, Etc)
-				Gave Child Pumpkin, Carrots, Squash (Yellow Or Orange Inside)
-				Gave Child Any Dark Green Leafy Vegetables
-				Gave Child Mangoes, Papayas, Other Vitamin A Fruits
-				Gave Child Any Other Fruits
-				Gave Child Liver, Heart, Other Organs
-				Gave Child Fish Or Shellfish
-				Gave Child Food Made From Beans, Peas, Lentils, Nuts
-				Gave Child Cheese, Yogurt, Other Milk Products
-				Gave Child Other Solid-Semisolid Food
-				Gave Child Yogurt
-				Gave Child Tinned, Powdered Or Fresh Milk
-				Gave Child Baby Formula
South Africa	2016	DHS	7	Gave Child Fortified Baby Food (Cerelac, Etc)
-				Gave Child Bread, Noodles, Other Made From Grains
-				Gave Child Potatoes, Cassava, Or Other Tubers
-				Gave Child Eggs
-				Gave Child Meat (Beef, Pork, Lamb, Chicken, Etc)
-				Gave Child Pumpkin, Carrots, Squash (Yellow Or Orange Inside)
-				Gave Child Any Dark Green Leafy Vegetables
-				Gave Child Mangoes, Papayas, Other Vitamin A Fruits
-				Gave Child Any Other Fruits
-				Gave Child Liver, Heart, Other Organs
-				Gave Child Fish Or Shellfish
-				Gave Child Food Made From Beans, Peas, Lentils, Nuts
-				Gave Child Cheese, Yogurt, Other Milk Products
-				Gave Child Oil, Fats, Butter, Products Made Of Them
-				Gave Child Chocolates, Sweets, Candies, Pastries, Etc
-				Gave Child Other Solid-Semisolid Food
-				Gave Child Any Salty Snacks
-				Gave Child Yogurt
-				Gave Child Tinned, Powdered Or Fresh Milk
-				Gave Child Baby Formula
State of Palestine	2019–20	MICS	6	Child Ate Yogurt Yesterday
-				Child Ate Fortified Baby Food (Gerber, Hero, Cerelac, Nestum, Etc.) Yesterday
-				Child Ate Foods Made From Grains Yesterday
-				Child Ate Pumpkin, Carrots, Squash Etc. That Are Yellow Or Orange Inside Yesterday
-				Child Ate White Potatoes, White Yams, Manioc, Cassava Etc. Any Other Foods Made
-				Child Ate Green Leafy Vegetables Yesterday
-				Child Ate Ripe Mangoes, Papayas Etc. Any Other Vitamin A-Rich Fruits Yesterday
-				Child Ate Other Fruits Or Vegetables Yesterday
-				Child Ate Liver, Kidney, Heart Or Other Organ Meat Yesterday
-				Child Ate Meat, Such As Beef, Pork, Lamb, Goat, Chicken, Duck Yesterday
-				Child Ate Eggs Yesterday
-				Child Ate Fresh Or Dried Fish Or Shellfish Yesterday
-				Child Ate Beans, Peas, Lentils Or Nuts Or Any Food Made From These Yesterday
-				Child Ate Cheese Or Other Food Made From Milk Yesterday
-				Child Ate Other Solid, Semi-Solid Or Soft Food Yesterday
-				Child Drank Infant Formula Yesterday
-				Child Drank Milk From Animals Yesterday
Sudan	2014	MICS	5	Child Drank Or Ate Yogurt Yesterday
-				Child Ate Fortified Baby Food (Cerelac Etc.)
-				Child Ate Foods Made From Grains
-				Child Ate Pumpkin, Carrots, Squash Etc.
-				Child Ate White Potatoes, White Yams, Manioc Etc.

© 2024 Karlsson O et al. *JAMA Network Open*.

Country	Year	Data	Wave*	Food variable label
-				Child Ate Green Leafy Vegetables
-				Child Ate Ripe Mangoes, Papayas
-				Child Ate Other Fruits Or Vegetables
-				Child Ateliver, Kidney, Heart Or Other Organ Meat
-				Child Ate Meat, Such As Beef, Pork, Lamb, Goat, Chicken, Duck
-				Child Ate Eggs
-				Child Ate Fresh Or Dried Fish Or Shellfish
-				Child Ate Beans, Lentils Or Nuts
-				Child Ate Cheese Or Other Food Made From Milk
-				Child Ate Other Solid, Semi-Solid Or Soft Food Yesterday
-				Child Drank Milk Yesterday
-				Child Drank Infant Formula Yesterday
Suriname	2018	MICS	6	Child Drank Or Ate Yogurt Yesterday
-				Child Ate Fortified Baby Food (Gerber, Hero, Cerelac, Nestum, Etc.) Yesterday
-				Child Ate Foods Made From Grains Yesterday
-				Child Ate Pumpkin, Carrots, Squash Etc. Yesterday
-				Child Ate White Potatoes, White Yams, Manioc, Cassava Etc. Yesterday
-				Child Ate Green Leafy Vegetables Yesterday
-				Child Ate Ripe Mangoes, Papayas Yesterday
-				Child Ate Other Fruits Or Vegetables Yesterday
-				Child Ate Liver, Kidney, Heart Or Other Organ Meat Yesterday
-				Child Ate Meat, Such As Beef, Pork, Lamb, Goat, Chicken, Duck Yesterday
-				Child Ate Eggs Yesterday
-				Child Ate Fresh Or Dried Fish Or Shellfish Yesterday
-				Child Ate Beans, Peas, Lentils Or Nuts Or Any Food Made From These Yesterday
-				Child Ate Cheese Or Other Food Made From Milk Yesterday
-				Child Ate Other Solid, Semi-Solid Or Soft Food Yesterday
-				Child Drank Infant Formula Yesterday
-				Child Drank Milk Yesterday
Tajikistan	2017	DHS	7	Gave Child Fortified Baby Food (Cerelac, Etc)
-				Gave Child Bread, Noodles, Other Made From Grains
-				Gave Child Potatoes, Cassava, Or Other Tubers
-				Gave Child Eggs
-				Gave Child Meat (Beef, Pork, Lamb, Chicken, Etc)
-				Gave Sweet Red Bell Pepper, Child Pumpkin, Carrots, Squash (Yellow Or Orange Ins
-				Gave Child Any Dark Green Leafy Vegetables
-				Gave Child Ripe Persimmons, Or Ripe Fresh Apricots, Dried Apricots Or Dried Peac
-				Gave Child Any Other Fruits
-				Gave Child Liver, Heart, Other Organs
-				Gave Child Fish Or Shellfish
-				Gave Child Food Made From Beans, Peas, Lentils, Nuts
-				Gave Child Cheese, Yogurt, Other Milk Products
-				Gave Child Other Solid-Semisolid Food
-				Gave Child Yogurt
-				Gave Child Tinned, Powdered Or Fresh Milk
-				Gave Child Baby Formula
Tanzania	2015–16	DHS	7	Gave Child Fortified Baby Food (Cerelac, Etc)
-				Gave Child Bread, Noodles, Other Made From Grains
-				Gave Child Potatoes, Cassava, Or Other Tubers
-				Gave Child Eggs
-				Gave Child Meat (Beef, Pork, Lamb, Chicken, Etc)
-				Gave Child Pumpkin, Carrots, Squash (Yellow Or Orange Inside)
-				Gave Child Any Dark Green Leafy Vegetables
-				Gave Child Mangoes, Papayas, Other Vitamin A Fruits
-				Gave Child Any Other Fruits
-				Gave Child Liver, Heart, Other Organs
-				Gave Child Fish Or Shellfish
-				Gave Child Food Made From Beans, Peas, Lentils, Nuts
-				Gave Child Cheese, Yogurt, Other Milk Products
-				Gave Child Other Solid-Semisolid Food
-				Gave Child Yogurt
-				Gave Child Tinned, Powdered Or Fresh Milk
-				Gave Child Baby Formula
Timor-Leste	2016	DHS	7	Gave Child Fortified Baby Food (Cerelac, Etc)
-				Gave Child Bread, Noodles, Other Made From Grains
-				Gave Child Potatoes, Cassava, Or Other Tubers
-				Gave Child Eggs
-				Gave Child Meat (Beef, Pork, Lamb, Chicken, Etc)
-				Gave Child Pumpkin, Carrots, Squash (Yellow Or Orange Inside)
-				Gave Child Any Dark Green Leafy Vegetables
-				Gave Child Mangoes, Papayas, Other Vitamin A Fruits

© 2024 Karlsson O et al. *JAMA Network Open*.

Country	Year	Data	Wave*	Food variable label
-				Gave Child Any Other Fruits
-				Gave Child Liver, Heart, Other Organs
-				Gave Child Fish Or Shellfish
-				Gave Child Food Made From Beans, Peas, Lentils, Nuts
-				Gave Child Cheese, Yogurt, Other Milk Products
-				Gave Child Other Solid-Semisolid Food
-				Gave Child Yogurt
-				Gave Child Tinned, Powdered Or Fresh Milk
-				Gave Child Baby Formula
Togo	2017	MICS	6	Lenfant A Bu Ou Mangé Des Yaourts Hier
-				Lenfant A Mangé De La Nourriture FortifiéE Pour BéBé (Cerelac, Gerber, Nest
-				Lenfant A Mangé Du Pain, Riz, PâTe, Bouillie Ou Autres Aliments Faits à Base
-				Lenfant A Mangé De La Courge, Carottes, Citrouille, Patate Douce, Etc. Qui Son
-				Lenfant A Mangé Des Pommes De Terre, De Ligname, Du Manioc Ou Autre Nourritur
-				Lenfant A Mangé Des LéGumes à Feuilles Vert Foncé
-				Lenfant A Mangé Des Mangues/Papayes Mures
-				Lenfant A Mangé Dautres Fruits Et LéGumes
-				Lenfant A Mangé Du Foie, Rognons, Coeur Ou Autres Abats
-				Lenfant A Mangé De La Viande De Boeuf, Porc, Agneau, ChèVre, Etc.
-				Lenfant A Mangé Des Oeufs
-				Lenfant A Mangé Du Poisson Frais Ou SéChé
-				Lenfant A Mangé Des Aliments à Base De Haricots, Pois, Etc.
-				Lenfant A Mangé Du Fromage Ou Autre Produit Laitier
-				Lenfant A Mangé Des Autres Produits Solides, Semi-Solides Ou Mous
-				Lenfant A Bu Une PréParation Pour BéBé Hier
-				Lenfant A Bu Du Lait Hier
Tonga	2019	MICS	6	Child Ate Yogurt Yesterday
-				Child Ate Fortified Baby Food (Gerber, Hero, Cerelac, Nestum, Etc.) Yesterday
-				Child Ate Foods Made From Grains Yesterday
-				Child Ate Pumpkin, Carrots, Squash Etc. That Are Yellow Or Orange Inside Yesterday
-				Child Ate White Potatoes, White Yams, Cassava, Or Any Other Foods Made From Root
-				Child Ate Any Dark Green, Leafy Vegetables, Taro Leaves
-				Child Ate Ripe Mangoes, Ripe Papayas, Ripe Banana
-				Child Ate Any Other Fruits Or Vegetables, Such As Water Melon, Apple, Pear, Most
-				Child Ate Liver, Kidney, Heart Or Other Organ Meat Yesterday
-				Child Ate Meat, Such As Beef, Pork, Lamb, Goat, Chicken, Duck Yesterday
-				Child Ate Eggs Yesterday
-				Child Ate Fresh Or Dried Fish Or Shellfish Yesterday
-				Child Ate Beans, Peas, Lentils Or Nuts Or Any Food Made From These Yesterday
-				Child Ate Cheese Or Other Food Made From Milk Yesterday
-				Child Ate Other Solid, Semi-Solid Or Soft Food Yesterday
-				Infant Formula, Such As Sma, Karicare, S-26
-				Milk From Animals, Such As Fresh, Tinned, Or Powdered Milk
Tunisia	2018	MICS	6	Child Drank Or Ate Yogurt Yesterday
-				Child Ate Fortified Baby Food (Gerber, Hero, Cerelac, Nestum, Etc.) Yesterday
-				Child Ate Foods Made From Grains Yesterday
-				Child Ate Pumpkin, Carrots, Squash Etc. Yesterday
-				Child Ate White Potatoes, White Yams, Manioc, Cassava Etc. Yesterday
-				Child Ate Green Leafy Vegetables Yesterday
-				Child Ate Ripe Mangoes, Papayas Yesterday
-				Child Ate Other Fruits Or Vegetables Yesterday
-				Child Ate Liver, Kidney, Heart Or Other Organ Meat Yesterday
-				Child Ate Meat, Such As Beef, Pork, Lamb, Goat, Chicken, Duck Yesterday
-				Child Ate Eggs Yesterday
-				Child Ate Fresh Or Dried Fish Or Shellfish Yesterday
-				Child Ate Beans, Peas, Lentils Or Nuts Or Any Food Made From These Yesterday
-				Child Ate Cheese Or Other Food Made From Milk Yesterday
-				Child Ate Other Solid, Semi-Solid Or Soft Food Yesterday
-				Child Drank Infant Formula Yesterday
-				Child Drank Milk Yesterday
Turkmenistan	2019	MICS	6	Child Ate Yogurt Or Yogurt/Kefir Yesterday
-				Child Ate Fortified Baby Food (Nutrilak, Mamako, Similak, Nestle, Etc.) Yesterday
-				Child Ate Foods Made From Grains Yesterday
-				Child Ate Pumpkin, Carrots Etc. That Are Yellow Or Orange Inside Yesterday
-				Child Ate Potatoes Etc. Any Other Foods Made From Roots Yesterday
-				Child Ate Green Leafy Vegetables Yesterday
-				Child Ate Ripe Persimmon, Dried Or Fresh Apricot Etc. Any Other Vitamin A-Rich F
-				Child Ate Other Fruits Or Vegetables Yesterday
-				Child Ate Liver, Kidney, Heart Or Other Organ Meat Yesterday
-				Child Ate Meat, Such As Beef, Pork, Lamb, Goat, Chicken, Duck Yesterday
-				Child Ate Eggs Yesterday

© 2024 Karlsson O et al. *JAMA Network Open*.

Country	Year	Data	Wave*	Food variable label
-				Child Ate Fish, Either Fresh Or Dried Yesterday
-				Child Ate Beans, Peas, Lentils, Mung Beans Or Nuts Or Any Food Made From These Y
-				Child Ate Cheese, White Cheese, Cottage Cheese Or Other Food Made From Milk Yest
-				Child Ate Other Solid, Semi-Solid Or Soft Food Yesterday
-				Child Drank Infant Formula Yesterday
-				Child Drank Milk From Animals Yesterday
Tuvalu	2019–20	MICS	6	Child Ate Yogurt Yesterday
-				Child Ate Fortified Baby Food (Gerber, Hero, Cerelac, Nestum, Etc.) Yesterday
-				Child Ate Foods Made From Grains Yesterday
-				Child Ate Pumpkin, Carrots, Squash Etc. That Are Yellow Or Orange Inside Yesterday
-				Child Ate White Potatoes, White Yams, Manioc, Cassava Etc. Any Other Foods Made
-				Child Ate Green Leafy Vegetables Yesterday
-				Child Ate Ripe Mangoes, Papayas Etc. Any Other Vitamin A-Rich Fruits Yesterday
-				Child Ate Other Fruits Or Vegetables Yesterday
-				Child Ate Liver, Kidney, Heart Or Other Organ Meat Yesterday
-				Child Ate Meat, Such As Beef, Pork, Lamb, Goat, Chicken, Duck Yesterday
-				Child Ate Eggs Yesterday
-				Child Ate Fresh Or Dried Fish Or Shellfish Yesterday
-				Child Ate Beans, Peas, Lentils Or Nuts Or Any Food Made From These Yesterday
-				Child Ate Cheese Or Other Food Made From Milk Yesterday
-				Child Ate Other Solid, Semi-Solid Or Soft Food Yesterday
-				Child Drank Infant Formula Yesterday
-				Child Drank Milk From Animals Yesterday
Uganda	2016	DHS	7	Gave Child Fortified Baby Food (Cerelac, Etc)
-				Gave Child Banana (Matooke, Ndiizi, Gonja)
-				Gave Child Chicken, Duck, Turkey, Pigeon, Other Poultry
-				Gave Child Bread, Noodles, Other Made From Grains
-				Gave Child Potatoes, Cassava, Or Other Tubers
-				Gave Child Eggs
-				Gave Child Meat (Beef, Pork, Lamb, Chicken, Etc)
-				Gave Child Pumpkin, Carrots, Squash (Yellow Or Orange Inside)
-				Gave Child Any Dark Green Leafy Vegetables
-				Gave Child Mangoes, Papayas, Other Vitamin A Fruits
-				Gave Child Any Other Fruits
-				Gave Child Liver, Heart, Other Organs
-				Gave Child Fish Or Shellfish
-				Gave Child Food Made From Beans, Peas, Lentils, Nuts
-				Gave Child Cheese, Yogurt, Other Milk Products
-				Gave Child Oil, Fats, Butter, Products Made Of Them
-				Gave Child Chocolates, Sweets, Candies, Pastries, Etc
-				Gave Child Other Solid-Semisolid Food
-				Gave Child Yogurt
-				Gave Child Tinned, Powdered Or Fresh Milk
-				Gave Child Baby Formula
Uzbekistan	2021–22	MICS	6	Child Ate Yogurt Yesterday
-				Child Ate Any Baby Food, Such As Commercially Fortified Baby Food, Like Heinz Or
-				Child Ate Bread, Rice, Noodles, Porridge, Or Other Foods Made From Grains, Like
-				Child Ate Pumpkin, Carrots, Sweet Red Pepper Or Squash That Are Yellow Or Orange
-				Child Ate White Potatoes Or Turnip
-				Child Ate Any Dark Green, Leafy Vegetables, Such As Spinach Leaves, Romaine Lett
-				Child Ate Persimmon, Peaches That Are Red Or Orange Inside, Apricots Fresh Or Dr
-				Child Ate Any Other Fruits Or Vegetables
-				Child Ate Liver, Kidney, Heart, Tongue Or Other Organ Meats
-				Child Ate Meat, Such As Beef, Pork, Lamb, Goat, Chicken, Duck Yesterday
-				Child Ate Eggs Yesterday
-				Child Ate Fresh Or Dried Fish Or Shellfish Yesterday
-				Child Ate Beans, Peas, Lentils Or Nuts Or Any Food Made From These Yesterday
-				Cheese, "Kurt", Brynza, Cottage Cheese Or Other Food Made From Animal Milk
-				Child Ate Other Solid, Semi-Solid Or Soft Food Yesterday
-				Child Drank Infant Formula Yesterday
-				Child Drank Milk From Animals Yesterday
Viet Nam	2020–21	MICS	6	Child Ate Yogurt Made From Animal Milk Yesterday
-				Child Ate Fortified Baby Food (Gerber, Hero, Cerelac, Nestum, Etc.) Yesterday
-				Child Ate Foods Made From Grains Yesterday
-				Child Ate Pumpkin, Carrots, Squash Etc. That Are Yellow Or Orange Inside Yesterday
-				Child Ate White Potatoes, White Yams, Manioc, Cassava Etc. Any Other Foods Made
-				Child Ate Green Leafy Vegetables Yesterday
-				Child Ate Ripe Mangoes, Papayas Etc. Any Other Vitamin A-Rich Fruits Yesterday
-				Child Ate Other Fruits Or Vegetables Yesterday
-				Child Ate Liver, Kidney, Heart Or Other Organ Meat Yesterday
-				Child Ate Meat, Such As Beef, Pork, Lamb, Goat, Chicken, Duck Yesterday

© 2024 Karlsson O et al. *JAMA Network Open*.

Country	Year	Data	Wave*	Food variable label
-				Child Ate Eggs Yesterday
-				Child Ate Fresh Or Dried Fish Or Shellfish Yesterday
-				Child Ate Beans, Peas, Lentils Or Nuts Or Any Food Made From These Yesterday
-				Child Ate Cheese Or Other Food Made From Milk Yesterday
-				Child Ate Other Solid, Semi-Solid Or Soft Food Yesterday
-				Infant Formula Such As Hipp, Pediasure, Optimum Gold, Meiji, Similac, Nan, Friso
-				Milk From Animals, Such As Fresh, Tinned, Or Powdered Milk
Yemen	2013	DHS	6	Gave Child Fortified Baby Food (Cerelac, Etc)
-				Gave Child Bread, Noodles, Other Made From Grains
-				Gave Child Potatoes, Cassava, Or Other Tubers
-				Gave Child Eggs
-				Gave Child Meat (Beef, Pork, Lamb, Chicken, Etc)
-				Gave Child Pumpkin, Carrots, Squash (Yellow Or Orange Inside)
-				Gave Child Any Dark Green Leafy Vegetables
-				Gave Child Mangoes, Papayas, Other Vitamin A Fruits
-				Gave Child Any Other Fruits And Vegetables
-				Gave Child Liver, Heart, Other Organs
-				Gave Child Fish Or Shellfish
-				Gave Child Food Made From Beans, Peas, Lentils, Nuts
-				Gave Child Cheese, Other Milk Products
-				Gave Child Oil, Fats, Butter, Products Made Of Them
-				Gave Child Chocolates, Sweets, Candies, Pastries, Etc
-				Gave Child Other Solid-Semisolid Food
-				Gave Child Yogurt
-				Gave Child Tinned, Powdered Or Fresh Milk
-				Gave Child Baby Formula
Zambia	2018–19	DHS	7	Gave Child Fortified Baby Food (Cerelac, Etc)
-				Gave Child Bread, Noodles, Other Made From Grains
-				Gave Child Potatoes, Cassava, Or Other Tubers
-				Gave Child Eggs
-				Gave Child Meat (Beef, Pork, Lamb, Chicken, Etc)
-				Gave Child Pumpkin, Carrots, Squash (Yellow Or Orange Inside)
-				Gave Child Any Dark Green Leafy Vegetables
-				Gave Child Mangoes, Papayas, Other Vitamin A Fruits
-				Gave Child Any Other Fruits
-				Gave Child Liver, Heart, Other Organs
-				Gave Child Fish Or Shellfish
-				Gave Child Food Made From Beans, Peas, Lentils, Nuts
-				Gave Child Cheese, Yogurt, Other Milk Products
-				Gave Child Other Solid-Semisolid Food
-				Gave Child Cs Foods: Caterpillar
-				Gave Child Yogurt
-				Gave Child Tinned, Powdered Or Fresh Milk
-				Gave Child Baby Formula
Zimbabwe	2019	MICS	6	Child Ate Yogurt Yesterday
-				Child Ate Fortified Baby Food (Gerber, Hero, Cerelac, Nestum, Etc.) Yesterday
-				Child Ate Foods Made From Grains Yesterday
-				Child Ate Pumpkin, Carrots, Squash Etc. That Are Yellow Or Orange Inside Yesterday
-				Child Ate White Potatoes, White Yams, Manioc, Cassava Etc. Any Other Foods Made
-				Child Ate Green Leafy Vegetables Yesterday
-				Child Ate Ripe Mangoes, Papayas Etc. Any Other Vitamin A-Rich Fruits Yesterday
-				Child Ate Other Fruits Or Vegetables Yesterday
-				Child Ate Liver, Kidney, Heart Or Other Organ Meat Yesterday
-				Child Ate Meat, Such As Beef, Pork, Lamb, Goat, Chicken, Duck Yesterday
-				Child Ate Eggs Yesterday
-				Child Ate Fresh Or Dried Fish Or Shellfish Yesterday
-				Child Ate Beans, Peas, Lentils Or Nuts Or Any Food Made From These Yesterday
-				Child Ate Cheese Or Other Food Made From Milk Yesterday
-				Child Ate Other Solid, Semi-Solid Or Soft Food Yesterday
-				Child Drank Infant Formula Yesterday
-				Child Drank Milk From Animals Yesterday

Notes: *Wave defines the questionnaire used for the survey. Data sources are Demographic and Health Surveys (DHS) and Multiple Indicator Cluster Surveys (MICS).

© 2024 Karlsson O et al. *JAMA Network Open*.

eTable 3. Zero-Food-Juice-Broth Prevalence and Estimated Number of Zero-Food-Juice-Broth Children

	Year	% [95% CI]	Number '000
Pooled		9.3 [9.1, 9.6]	12,456
South Asia		14.3 [13.8, 14.8]	7,304
India	2019–21	17.5 [17.0, 17.9]	6,082
Pakistan	2017–18	9.0 [6.9, 11.5]	823
Afghanistan	2015	7.3 [6.1, 8.8]	135
Bangladesh	2019	5.2 [4.6, 5.9]	234
Nepal	2019	3.4 [2.5, 4.4]	29
Maldives	2016–17	2.3 [1.2, 4.7]	0.3
West & Central Africa		9.5 [9.0, 9.9]	2,645
Guinea	2018	20.1 [17.7, 22.8]	134
Mali	2018	19.1 [17.1, 21.3]	227
Liberia	2019–20	18.1 [15.6, 20.9]	40
Burkina Faso	2010	16.2 [14.7, 17.8]	154
Benin	2017–18	15.6 [14.1, 17.3]	94
Mauritania	2019–21	13.6 [12.0, 15.4]	28
Senegal	2019	13.5 [10.8, 16.6]	102
Guinea-Bissau	2018–19	12.8 [11.0, 14.9]	11
Niger	2012	10.5 [9.1, 11.9]	124
Chad	2019	10.1 [8.9, 11.4]	100
Sierra Leone	2019	10.0 [8.7, 11.5]	36
Cote d'Ivoire	2016	9.3 [7.9, 10.9]	113
Central African Republic	2018–19	8.7 [7.4, 10.3]	25
Togo	2017	8.7 [6.9, 11.0]	32
Ghana	2017–18	8.5 [7.0, 10.3]	110
Nigeria	2021	7.9 [7.1, 8.8]	863
Cameroon	2018–19	7.4 [6.2, 8.9]	96
Gambia	2019–20	7.0 [5.8, 8.4]	9
Congo DR	2017–18	6.8 [5.7, 8.0]	330
Sao Tome and Principe	2019	4.8 [3.2, 7.3]	0.5
Congo	2014–15	4.5 [3.6, 5.7]	11
Gabon	2012	1.9 [1.3, 2.6]	2
Eastern & Southern Africa		6.6 [5.8, 7.6]	1,587
Ethiopia	2019	12.6 [9.5, 16.6]	660
Sudan	2014	10.1 [8.9, 11.5]	190
Angola	2015–16	8.0 [6.9, 9.2]	131
Mozambique	2011	6.2 [5.1, 7.5]	82
Namibia	2013	5.5 [4.1, 7.3]	5
Rwanda	2019–20	4.8 [3.9, 6.0]	27
Uganda	2016	4.6 [3.9, 5.4]	96
Burundi	2016–17	4.6 [3.9, 5.3]	28
Zambia	2018–19	4.1 [3.4, 5.1]	38
South Africa	2016	4.1 [2.6, 6.4]	71
Kenya	2014	3.7 [3.0, 4.7]	75
Comoros	2012	3.5 [2.3, 5.4]	1
Malawi	2019–20	3.5 [2.8, 4.4]	32
Zimbabwe	2019	3.3 [2.5, 4.4]	23
Madagascar	2021	3.3 [2.7, 4.2]	42
Lesotho	2018	2.1 [1.2, 3.5]	2
Tanzania	2015–16	1.8 [1.2, 2.5]	50
Eswatini	2014	0.9 [0.4, 1.8]	0.4
Middle East & North Africa		3.7 [3.4, 4.1]	340
Egypt	2014	5.6 [4.9, 6.5]	211
Yemen	2013	3.7 [3.0, 4.6]	49
Jordan	2017–18	3.0 [2.1, 4.2]	11
Algeria	2018–19	2.3 [1.8, 3.0]	35
Iraq	2018	2.0 [1.5, 2.6]	33
State of Palestine	2019–20	1.3 [0.8, 2.0]	3
Tunisia	2018	0.1 [0.0, 0.6]	0.3
Europe & Central Asia		3.1 [2.6, 3.6]	95
Tajikistan	2017	7.2 [5.8, 8.9]	27
Albania	2017–18	3.5 [2.1, 5.6]	2

© 2024 Karlsson O et al. *JAMA Network Open*.

	Year	% [95% CI]	Number '000
Kazakhstan	2015	3.3 [2.2, 4.9]	19
Armenia	2015–16	2.8 [1.6, 4.9]	2
Uzbekistan	2021–22	2.5 [1.4, 4.5]	29
Kyrgyzstan	2018	2.3 [1.4, 3.6]	6
Turkmenistan	2019	1.4 [0.8, 2.4]	3
Montenegro	2018	1.2 [0.5, 2.9]	0.1
Georgia	2018	1.1 [0.4, 2.7]	1
North Macedonia	2018–19	0.7 [0.1, 4.6]	0.2
Serbia	2019	0.7 [0.2, 2.7]	1
Belarus	2019	0.4 [0.2, 1.1]	1
Kosovo	2019–20	0.2 [0.0, 1.7]	0.1
East Asia & Pacific		2.7 [2.3, 3.1]	313
Timor-Leste	2016	13.3 [11.4, 15.4]	6
Papua New Guinea	2016–18	7.0 [5.7, 8.6]	24
Myanmar	2015–16	5.1 [3.7, 7.2]	70
Cambodia	2014	3.6 [2.6, 5.0]	18
Samoa	2019–20	3.2 [2.1, 4.7]	0.3
Lao	2017	2.5 [2.0, 3.1]	6
Kiribati	2018–19	2.2 [1.3, 3.6]	0.1
Indonesia	2017	2.1 [1.7, 2.7]	148
Viet Nam	2020–21	1.8 [1.0, 3.1]	40
Mongolia	2018	1.5 [0.8, 2.5]	2
Fiji	2021	1.1 [0.6, 2.2]	0.3
Tonga	2019	1.1 [0.4, 3.3]	0.04
Tuvalu	2019–20	0.7 [0.1, 4.6]	0.00
Latin America & Caribbean		1.7 [1.1, 2.6]	106
Guatemala	2014–15	3.6 [2.9, 4.5]	21
Guyana	2014	3.1 [1.9, 5.1]	1
Suriname	2018	2.5 [1.5, 4.2]	0.4
Peru	2012	2.1 [1.6, 2.8]	18
Belize	2015–16	2.1 [1.0, 4.3]	0.2
Haiti	2016–17	2.1 [1.5, 2.9]	8
Paraguay	2016	2.0 [1.1, 3.5]	4
Mexico	2015	1.4 [0.5, 3.9]	44
Honduras	2019	1.3 [0.9, 2.0]	4
El Salvador	2014	1.0 [0.6, 1.8]	2
Cuba	2019	1.0 [0.6, 1.7]	2
Dominican Republic	2019	0.5 [0.3, 0.9]	1
Costa Rica	2018	0.1 [0.0, 0.6]	0.1

Notes: The number of zero-food-juice-broth children was estimated using the estimated zero-food-juice-broth prevalence and the population of children 6–23 months old obtained from the United Nation World Population Prospects linked to the country and year of survey. Estimates refer to the latest survey in each country. 95% confidence intervals (CI) were adjusted for clustering at the level of primary sampling units. Estimates were weighted using sampling weights rescaled to sum up the population 6–23 months old in the country and year of survey.

© 2024 Karlsson O et al. *JAMA Network Open*.

eTable 4. Zero-Food-Juice-Broth and Nonbreastfed Prevalence and Estimated Number of Zero-Food-Juice-Broth and Nonbreastfed Children

	Year	% [95% CI]	Number '000
Pooled		0.6 [0.6, 0.7]	846
South Asia		1.0 [0.9, 1.1]	502
India	2019–21	1.3 [1.2, 1.5]	462
Afghanistan	2015	0.4 [0.2, 0.6]	7
Pakistan	2017–18	0.3 [0.1, 0.9]	28
Maldives	2016–17	0.1 [0.0, 0.6]	0.02
Bangladesh	2019	0.1 [0.0, 0.2]	3
Nepal	2019	0.1 [0.0, 0.3]	1
Eastern & Southern Africa		0.7 [0.5, 1.0]	178
Angola	2015–16	2.2 [1.6, 3.0]	36
Ethiopia	2019	1.5 [0.8, 2.8]	79
South Africa	2016	1.3 [0.6, 2.6]	22
Mozambique	2011	0.6 [0.4, 1.0]	8
Lesotho	2018	0.4 [0.2, 1.2]	0.3
Namibia	2013	0.4 [0.2, 1.0]	0.4
Madagascar	2021	0.3 [0.2, 0.6]	4
Comoros	2012	0.3 [0.1, 1.4]	0.1
Sudan	2014	0.3 [0.1, 0.6]	6
Kenya	2014	0.3 [0.1, 0.6]	5
Tanzania	2015–16	0.3 [0.1, 0.7]	7
Uganda	2016	0.2 [0.1, 0.4]	3
Zambia	2018–19	0.2 [0.1, 0.4]	1
Rwanda	2019–20	0.1 [0.0, 0.3]	0.4
Malawi	2019–20	0.1 [0.0, 0.2]	1
Burundi	2016–17	0.1 [0.0, 0.3]	0.4
Zimbabwe	2019	0.0 [0.0, 0.2]	0.2
Eswatini	2014	NA	0.00
West & Central Africa		0.4 [0.4, 0.5]	120
Guinea	2018	3.4 [2.5, 4.6]	22
Mauritania	2019–21	1.4 [0.9, 2.0]	3
Benin	2017–18	1.1 [0.8, 1.6]	7
Mali	2018	1.0 [0.7, 1.5]	12
Cameroon	2018–19	0.8 [0.5, 1.3]	11
Central African Republic	2018–19	0.8 [0.5, 1.4]	2
Chad	2019	0.8 [0.5, 1.2]	8
Liberia	2019–20	0.8 [0.3, 2.2]	2
Sierra Leone	2019	0.7 [0.4, 1.2]	3
Congo	2014–15	0.5 [0.3, 0.9]	1
Burkina Faso	2010	0.5 [0.3, 0.7]	4
Congo DR	2017–18	0.4 [0.2, 0.7]	18
Niger	2012	0.4 [0.2, 0.8]	4
Gabon	2012	0.3 [0.1, 0.6]	0.2
Cote d'Ivoire	2016	0.2 [0.0, 0.7]	2
Nigeria	2021	0.1 [0.1, 0.3]	16
Senegal	2019	0.1 [0.0, 0.7]	1
Guinea-Bissau	2018–19	0.1 [0.0, 0.3]	0.1
Gambia	2019–20	0.1 [0.0, 0.6]	0.1
Ghana	2017–18	0.1 [0.0, 0.3]	1
Togo	2017	NA	0.00
Sao Tome and Principe	2019	NA	0.00
Middle East & North Africa		0.2 [0.1, 0.3]	19
Jordan	2017–18	0.6 [0.3, 1.3]	2
Algeria	2018–19	0.3 [0.1, 0.6]	4
Egypt	2014	0.2 [0.1, 0.4]	8
Yemen	2013	0.2 [0.1, 0.4]	3
Iraq	2018	0.1 [0.1, 0.3]	2
State of Palestine	2019–20	0.0 [0.0, 0.1]	0.04
Tunisia	2018	NA	0.00
East Asia & Pacific		0.1 [0.1, 0.2]	14
Timor-Leste	2016	2.3 [1.6, 3.3]	1

	Year	% [95% CI]	Number '000
Papua New Guinea	2016–18	0.4 [0.2, 0.9]	2
Myanmar	2015–16	0.3 [0.1, 1.2]	5
Samoa	2019–20	0.2 [0.1, 0.9]	0.02
Cambodia	2014	0.2 [0.1, 0.4]	1
Mongolia	2018	0.1 [0.0, 0.7]	0.1
Indonesia	2017	0.1 [0.0, 0.2]	5
Viet Nam	2020–21	0.1 [0.0, 0.3]	1
Lao	2017	0.0 [0.0, 0.1]	0.05
Fiji	2021	NA	0.00
Kiribati	2018–19	NA	0.00
Tonga	2019	NA	0.00
Tuvalu	2019–20	NA	0.00
Europe & Central Asia		0.1 [0.0, 0.1]	2
Albania	2017–18	0.4 [0.1, 1.3]	0.2
Tajikistan	2017	0.3 [0.1, 0.7]	1
Armenia	2015–16	0.2 [0.0, 1.2]	0.1
Kyrgyzstan	2018	0.1 [0.0, 0.4]	0.2
Uzbekistan	2021–22	NA	0.00
Kazakhstan	2015	NA	0.00
Turkmenistan	2019	NA	0.00
Belarus	2019	NA	0.00
Serbia	2019	NA	0.00
Georgia	2018	NA	0.00
North Macedonia	2018–19	NA	0.00
Kosovo	2019–20	NA	0.00
Montenegro	2018	NA	0.00
Latin America & Caribbean		0.0 [0.0, 0.0]	1
Honduras	2019	0.2 [0.0, 0.7]	1
Dominican Republic	2019	0.1 [0.0, 0.5]	0.4
Suriname	2018	0.1 [0.0, 0.9]	0.02
Paraguay	2016	0.1 [0.0, 0.6]	0.2
Cuba	2019	0.1 [0.0, 0.2]	0.1
Guatemala	2014–15	0.0 [0.0, 0.1]	0.1
Peru	2012	0.0 [0.0, 0.1]	0.1
Mexico	2015	NA	0.00
Haiti	2016–17	NA	0.00
El Salvador	2014	NA	0.00
Costa Rica	2018	NA	0.00
Guyana	2014	NA	0.00
Belize	2015–16	NA	0.00

Notes: The number of zero-food-juice-broth and non-breastfed children was estimated using the estimated zero-food-juice-broth and non-breastfed prevalence and the population of children 6–23 months old obtained from the United Nation World Population Prospects linked to the country and year of survey. Estimates refer to the latest survey in each country. 95% confidence intervals (CI) were adjusted for clustering at the level of primary sampling units. Estimates were weighted using sampling weights rescaled to sum up the population 6–23 months old in the country and year of survey. NA is shown where countries did not have any zero-food-juice-broth and non-breastfed children.

© 2024 Karlsson O et al. *JAMA Network Open*.

eTable 5. Zero-Food Prevalence: by Child's Age

	Year	6–11 months<br>% [95% CI]	12–17 months<br>% [95% CI]	18–23 months<br>% [95% CI]
Pooled		20.0 [19.4, 20.5]	6.6 [6.2, 6.9]	4.1 [3.8, 4.6]
East Asia & Pacific		7.3 [6.2, 8.5]	1.1 [0.7, 1.5]	0.4 [0.3, 0.7]
Myanmar	2015–16	11.3 [8.1, 15.6]	4.0 [2.1, 7.6]	1.0 [0.3, 3.3]
Papua New Guinea	2016–18	16.2 [12.8, 20.4]	3.6 [2.4, 5.3]	3.4 [2.1, 5.5]
Cambodia	2014	9.7 [7.0, 13.3]	1.1 [0.4, 2.6]	0.1 [0.0, 0.2]
Viet Nam	2020–21	6.4 [3.6, 11.1]	1.0 [0.5, 2.0]	0.2 [0.0, 0.9]
Indonesia	2017	6.1 [4.8, 7.7]	0.3 [0.1, 0.5]	0.2 [0.1, 0.6]
Timor-Leste	2016	25.7 [21.7, 30.1]	11.7 [9.1, 15.0]	7.9 [5.5, 11.2]
Fiji	2021	2.2 [1.0, 4.6]	1.3 [0.4, 4.1]	0.4 [0.1, 2.6]
Samoa	2019–20	7.6 [4.9, 11.7]	2.3 [1.1, 4.6]	2.5 [1.2, 5.4]
Mongolia	2018	3.5 [2.0, 6.0]	0.9 [0.4, 2.4]	0.3 [0.0, 2.4]
Lao	2017	6.6 [5.2, 8.2]	1.5 [0.9, 2.7]	0.5 [0.2, 1.1]
Tonga	2019	1.8 [0.6, 5.8]	1.6 [0.4, 6.3]	NA
Tuvalu	2019–20	2.0 [0.3, 13.2]	NA	NA
Kiribati	2018–19	5.8 [3.3, 10.0]	1.3 [0.4, 4.0]	NA
Eastern & Southern Africa		14.4 [12.9, 16.0]	5.1 [3.9, 6.7]	3.2 [2.2, 4.7]
Comoros	2012	12.6 [8.8, 17.6]	4.8 [2.6, 8.9]	1.5 [0.6, 4.0]
Rwanda	2019–20	13.1 [10.6, 16.0]	1.3 [0.6, 2.5]	0.3 [0.1, 1.1]
Angola	2015–16	18.1 [15.6, 20.9]	8.3 [6.7, 10.3]	4.3 [3.1, 5.8]
Kenya	2014	8.8 [6.9, 11.1]	3.0 [1.7, 5.4]	1.7 [0.9, 3.4]
Zambia	2018–19	9.0 [7.2, 11.3]	2.9 [1.9, 4.2]	1.3 [0.7, 2.3]
Mozambique	2011	11.8 [9.5, 14.6]	4.6 [3.2, 6.5]	2.4 [1.3, 4.2]
Malawi	2019–20	8.8 [6.8, 11.2]	2.2 [1.4, 3.2]	2.7 [1.9, 3.9]
Burundi	2016–17	11.7 [9.8, 13.9]	1.8 [1.1, 2.9]	1.5 [0.8, 2.5]
Eswatini	2014	2.8 [1.3, 5.6]	NA	NA
Namibia	2013	10.7 [8.1, 14.1]	4.2 [2.6, 6.9]	2.9 [1.4, 6.3]
Zimbabwe	2019	7.7 [5.7, 10.3]	2.2 [1.1, 4.2]	0.3 [0.1, 1.1]
Uganda	2016	12.2 [10.5, 14.1]	2.1 [1.4, 3.0]	1.3 [0.8, 2.2]
South Africa	2016	9.6 [5.7, 15.8]	1.3 [0.5, 3.1]	2.3 [1.0, 5.4]
Tanzania	2015–16	5.0 [3.5, 7.0]	0.4 [0.1, 1.3]	0.2 [0.0, 1.5]
Sudan	2014	23.1 [20.3, 26.1]	6.1 [4.6, 7.9]	4.7 [3.2, 6.7]
Ethiopia	2019	23.8 [18.3, 30.4]	11.9 [7.5, 18.3]	7.7 [3.7, 15.1]
Madagascar	2021	9.1 [7.4, 11.3]	2.5 [1.6, 3.9]	1.1 [0.6, 2.1]
Lesotho	2018	4.5 [2.4, 8.4]	1.4 [0.3, 5.4]	1.2 [0.4, 3.3]
Europe & Central Asia		9.9 [8.3, 11.9]	1.5 [1.0, 2.1]	0.6 [0.3, 1.0]
Belarus	2019	1.3 [0.5, 3.6]	NA	NA
Uzbekistan	2021–22	6.5 [3.3, 12.1]	3.0 [1.3, 6.8]	1.2 [0.3, 4.9]
Kazakhstan	2015	13.3 [9.0, 19.2]	0.4 [0.1, 1.3]	0.1 [0.0, 0.6]
Tajikistan	2017	21.6 [17.7, 26.1]	4.3 [2.9, 6.3]	1.9 [1.0, 3.6]
Georgia	2018	2.9 [1.2, 7.2]	0.3 [0.0, 2.5]	NA
Serbia	2019	1.9 [0.5, 7.4]	NA	NA
Turkmenistan	2019	3.5 [1.9, 6.4]	0.5 [0.1, 3.5]	NA
Kyrgyzstan	2018	7.1 [4.6, 10.9]	0.4 [0.1, 1.6]	0.2 [0.0, 1.1]
North Macedonia	2018–19	2.1 [0.3, 13.9]	NA	NA
Montenegro	2018	3.6 [1.4, 8.8]	1.2 [0.3, 5.2]	0.5 [0.1, 3.4]
Kosovo	2019–20	0.6 [0.1, 4.5]	NA	NA
Albania	2017–18	14.9 [8.9, 23.9]	2.2 [1.1, 4.1]	1.6 [0.5, 4.8]
Armenia	2015–16	7.4 [4.3, 12.6]	0.4 [0.1, 2.7]	0.6 [0.1, 4.0]
Latin America & Caribbean		4.9 [3.1, 7.4]	0.5 [0.3, 0.8]	0.2 [0.1, 0.3]
Guatemala	2014–15	10.1 [8.2, 12.4]	0.5 [0.2, 1.7]	0.1 [0.0, 0.5]
Suriname	2018	6.1 [3.5, 10.4]	0.8 [0.3, 2.1]	0.7 [0.2, 2.7]
Paraguay	2016	6.4 [3.6, 11.0]	0.5 [0.2, 1.5]	0.1 [0.0, 0.6]
Mexico	2015	3.9 [1.3, 10.7]	0.4 [0.1, 1.0]	0.0 [0.0, 0.1]
El Salvador	2014	3.4 [2.0, 5.6]	NA	0.1 [0.0, 0.7]
Costa Rica	2018	0.5 [0.1, 2.0]	NA	NA
Cuba	2019	2.8 [1.6, 4.9]	1.3 [0.6, 2.7]	0.4 [0.2, 0.9]
Dominican Republic	2019	1.9 [0.9, 4.1]	0.4 [0.1, 1.2]	0.9 [0.3, 3.0]
Haiti	2016–17	6.9 [4.8, 9.7]	1.8 [1.0, 3.1]	0.7 [0.3, 2.0]
Honduras	2019	3.3 [2.1, 5.1]	1.5 [0.8, 2.8]	0.9 [0.4, 2.1]
Peru	2012	6.3 [4.7, 8.3]	NA	0.1 [0.0, 0.2]

© 2024 Karlsson O et al. *JAMA Network Open*.

	Year	6–11 months<br>% [95% CI]	12–17 months<br>% [95% CI]	18–23 months<br>% [95% CI]
Guyana	2014	9.1 [5.7, 14.3]	1.6 [0.7, 3.8]	2.2 [0.8, 6.0]
Belize	2015–16	7.5 [4.0, 13.6]	0.6 [0.1, 3.8]	0.4 [0.1, 2.7]
Middle East & North Africa		9.8 [8.8, 10.9]	2.5 [2.1, 3.1]	1.0 [0.7, 1.4]
Yemen	2013	8.6 [7.0, 10.6]	2.4 [1.7, 3.6]	0.7 [0.4, 1.6]
Tunisia	2018	0.8 [0.2, 2.7]	0.4 [0.1, 3.1]	0.2 [0.0, 1.5]
State of Palestine	2019–20	4.2 [2.7, 6.5]	0.4 [0.1, 1.1]	NA
Egypt	2014	14.0 [12.1, 16.1]	4.0 [3.0, 5.3]	0.8 [0.4, 1.7]
Iraq	2018	5.8 [4.4, 7.6]	1.1 [0.7, 1.8]	1.2 [0.7, 1.9]
Algeria	2018–19	6.6 [5.1, 8.5]	2.0 [1.2, 3.1]	1.5 [0.8, 2.9]
Jordan	2017–18	8.2 [5.8, 11.3]	0.9 [0.4, 2.0]	0.9 [0.5, 1.6]
South Asia		28.9 [28.0, 29.9]	10.2 [9.6, 10.8]	7.2 [6.5, 8.1]
India	2019–21	34.5 [33.6, 35.4]	13.6 [12.9, 14.2]	8.7 [8.2, 9.3]
Afghanistan	2015	17.5 [14.7, 20.6]	4.4 [3.4, 5.6]	4.5 [2.8, 7.2]
Maldives	2016–17	6.1 [2.9, 12.3]	0.5 [0.1, 2.1]	0.2 [0.0, 1.6]
Bangladesh	2019	12.2 [10.7, 13.9]	2.7 [2.0, 3.5]	2.0 [1.4, 2.7]
Nepal	2019	7.4 [5.4, 10.0]	2.6 [1.5, 4.5]	2.2 [1.2, 3.9]
Pakistan	2017–18	19.2 [15.8, 23.2]	4.4 [2.9, 6.6]	4.8 [1.9, 11.6]
West & Central Africa		21.0 [20.0, 22.1]	6.3 [5.7, 6.9]	3.3 [2.9, 3.7]
Chad	2019	21.0 [18.5, 23.8]	9.6 [8.0, 11.5]	8.6 [6.7, 11.0]
Sao Tome and Principe	2019	14.6 [9.4, 21.9]	5.7 [2.8, 11.1]	0.6 [0.1, 4.0]
Mauritania	2019–21	27.3 [23.9, 30.9]	7.1 [5.5, 9.1]	5.5 [3.9, 7.7]
Liberia	2019–20	39.3 [33.7, 45.2]	9.4 [6.8, 12.9]	2.4 [1.2, 4.7]
Benin	2017–18	35.4 [32.5, 38.3]	11.1 [9.1, 13.4]	6.2 [4.8, 8.0]
Niger	2012	26.5 [23.7, 29.6]	6.8 [5.2, 8.9]	4.4 [2.7, 6.9]
Guinea	2018	38.6 [33.9, 43.6]	17.6 [14.5, 21.3]	7.2 [4.8, 10.7]
Cote d'Ivoire	2016	22.0 [18.8, 25.7]	4.9 [3.3, 7.0]	0.6 [0.3, 1.4]
Senegal	2019	28.8 [23.6, 34.5]	8.2 [5.1, 13.0]	1.3 [0.6, 2.9]
Congo DR	2017–18	13.2 [11.1, 15.6]	5.7 [4.0, 8.2]	2.9 [2.0, 4.3]
Congo	2014–15	10.4 [8.1, 13.1]	3.4 [2.2, 5.2]	1.8 [0.9, 3.3]
Guinea-Bissau	2018–19	29.1 [25.2, 33.5]	7.9 [5.9, 10.5]	4.6 [3.1, 6.8]
Cameroon	2018–19	18.6 [15.5, 22.2]	2.9 [1.9, 4.4]	2.1 [1.2, 3.7]
Gabon	2012	3.4 [2.3, 5.1]	2.1 [1.0, 4.3]	0.5 [0.2, 1.3]
Ghana	2017–18	16.8 [13.1, 21.2]	6.7 [4.7, 9.3]	3.1 [1.8, 5.4]
Gambia	2019–20	17.7 [14.7, 21.1]	3.1 [2.0, 4.8]	0.6 [0.3, 1.3]
Central African Republic	2018–19	19.2 [16.3, 22.5]	12.6 [10.0, 15.6]	9.5 [7.2, 12.4]
Burkina Faso	2010	35.8 [33.0, 38.7]	7.3 [5.6, 9.6]	4.7 [3.4, 6.4]
Togo	2017	15.4 [11.9, 19.7]	6.9 [4.0, 11.9]	5.6 [3.4, 9.0]
Mali	2018	45.8 [41.6, 50.1]	9.8 [7.9, 12.2]	5.8 [4.0, 8.4]
Nigeria	2021	18.4 [16.4, 20.6]	4.7 [3.8, 5.9]	2.5 [1.8, 3.4]
Sierra Leone	2019	23.1 [19.7, 26.8]	5.4 [3.9, 7.3]	1.9 [1.0, 3.5]

Notes: 95% confidence intervals (CI) were adjusted for clustering at the level of primary sampling units. Estimates were weighted using sampling weights rescaled to sum up the population 6–23 months old in the country and year of survey. NA is shown where countries did not have any zero-food children.

© 2024 Karlsson O et al. *JAMA Network Open*.

eTable 6. Zero-Food Prevalence: by Child's Sex

	Year	Boys<br>% [95% CI]	Girls<br>% [95% CI]
Pooled		10.5 [10.1, 10.9]	10.4 [10.0, 10.7]
East Asia & Pacific		3.2 [2.6, 3.9]	2.5 [2.1, 3.1]
Viet Nam	2020–21	2.7 [1.5, 4.7]	1.8 [0.8, 3.9]
Papua New Guinea	2016–18	7.1 [5.6, 9.0]	9.2 [7.1, 11.9]
Myanmar	2015–16	6.5 [4.2, 9.8]	4.4 [2.8, 6.8]
Fiji	2021	1.5 [0.6, 3.7]	1.2 [0.5, 3.0]
Kiribati	2018–19	3.3 [1.8, 5.9]	1.4 [0.5, 3.5]
Tonga	2019	0.8 [0.2, 3.3]	1.4 [0.4, 4.3]
Lao	2017	2.5 [1.9, 3.4]	3.4 [2.5, 4.5]
Samoa	2019–20	4.4 [2.5, 7.6]	3.8 [2.3, 6.1]
Tuvalu	2019–20	1.3 [0.2, 9.1]	NA
Timor-Leste	2016	15.7 [13.1, 18.8]	15.4 [12.7, 18.6]
Mongolia	2018	0.8 [0.4, 1.4]	2.6 [1.4, 4.7]
Indonesia	2017	2.5 [1.8, 3.3]	1.9 [1.4, 2.6]
Cambodia	2014	3.9 [2.6, 5.8]	3.6 [2.3, 5.4]
Eastern & Southern Africa		8.6 [7.3, 10.0]	7.0 [6.0, 8.1]
Zambia	2018–19	3.7 [2.8, 4.8]	5.2 [4.1, 6.7]
Rwanda	2019–20	5.5 [4.2, 7.1]	4.4 [3.3, 6.0]
Lesotho	2018	2.7 [1.4, 5.0]	2.1 [0.8, 5.0]
Mozambique	2011	7.0 [5.4, 8.9]	6.1 [4.9, 7.7]
South Africa	2016	5.4 [3.2, 9.2]	3.4 [1.7, 6.5]
Eswatini	2014	0.7 [0.2, 2.1]	1.1 [0.5, 2.8]
Tanzania	2015–16	1.7 [1.0, 2.7]	2.0 [1.2, 3.2]
Namibia	2013	5.9 [4.1, 8.3]	6.9 [4.9, 9.6]
Sudan	2014	11.8 [10.0, 14.0]	11.5 [10.0, 13.2]
Burundi	2016–17	5.2 [4.2, 6.4]	5.0 [4.0, 6.3]
Uganda	2016	6.0 [5.0, 7.1]	5.4 [4.4, 6.6]
Angola	2015–16	10.8 [9.1, 12.8]	10.3 [8.6, 12.3]
Madagascar	2021	4.6 [3.6, 5.9]	4.4 [3.3, 5.8]
Comoros	2012	8.3 [5.2, 12.9]	4.8 [3.0, 7.9]
Ethiopia	2019	17.2 [12.5, 23.1]	12.1 [8.5, 16.9]
Zimbabwe	2019	3.3 [2.3, 4.8]	3.6 [2.3, 5.4]
Kenya	2014	4.8 [3.5, 6.6]	4.4 [3.3, 5.8]
Malawi	2019–20	5.1 [4.0, 6.6]	3.9 [3.0, 5.1]
Europe & Central Asia		4.1 [3.1, 5.3]	4.0 [3.3, 5.0]
Georgia	2018	0.9 [0.3, 3.0]	1.4 [0.4, 4.7]
Montenegro	2018	1.6 [0.5, 5.4]	2.1 [0.8, 5.1]
Serbia	2019	0.2 [0.0, 1.2]	1.3 [0.3, 6.3]
Belarus	2019	0.4 [0.1, 1.7]	0.4 [0.1, 1.7]
Kosovo	2019–20	0.5 [0.1, 3.2]	NA
Tajikistan	2017	10.3 [8.0, 13.1]	8.2 [6.3, 10.5]
Armenia	2015–16	3.1 [1.6, 6.0]	3.2 [1.5, 6.3]
Kazakhstan	2015	4.4 [2.1, 9.2]	4.9 [3.0, 8.0]
Turkmenistan	2019	1.5 [0.8, 3.0]	1.2 [0.5, 3.0]
Kyrgyzstan	2018	2.1 [1.1, 4.0]	3.2 [1.7, 5.6]
Uzbekistan	2021–22	3.5 [1.8, 6.5]	3.9 [2.0, 7.4]
North Macedonia	2018–19	1.3 [0.2, 8.6]	NA
Albania	2017–18	7.6 [4.0, 14.2]	5.7 [3.3, 9.7]
Latin America & Caribbean		2.2 [1.1, 4.2]	1.6 [1.3, 1.9]
Haiti	2016–17	3.1 [2.1, 4.6]	3.1 [2.1, 4.7]
Belize	2015–16	3.1 [1.6, 6.2]	2.6 [1.1, 6.2]
Cuba	2019	1.9 [1.1, 3.2]	1.3 [0.6, 2.4]
Paraguay	2016	2.3 [1.4, 3.9]	2.0 [0.9, 4.6]
Peru	2012	1.7 [1.1, 2.7]	2.7 [1.9, 3.9]
Suriname	2018	3.2 [1.6, 6.0]	2.4 [1.2, 4.7]
Honduras	2019	1.6 [1.0, 2.6]	2.1 [1.3, 3.4]
El Salvador	2014	0.7 [0.3, 1.3]	1.7 [0.9, 3.1]
Guatemala	2014–15	3.6 [2.7, 4.8]	4.1 [3.0, 5.4]
Guyana	2014	3.3 [1.6, 6.8]	5.4 [3.6, 8.1]
Costa Rica	2018	0.1 [0.0, 0.5]	0.2 [0.0, 0.9]

© 2024 Karlsson O et al. *JAMA Network Open*.

	Year	Boys<br>% [95% CI]	Girls<br>% [95% CI]
Mexico	2015	2.3 [0.6, 8.0]	0.7 [0.4, 1.2]
Dominican Republic	2019	0.7 [0.4, 1.4]	1.5 [0.6, 3.5]
Middle East & North Africa		4.7 [4.2, 5.4]	4.5 [4.0, 5.2]
Egypt	2014	6.7 [5.6, 8.0]	6.9 [5.8, 8.3]
Jordan	2017–18	3.7 [2.5, 5.5]	3.8 [2.5, 5.5]
State of Palestine	2019–20	1.7 [0.9, 3.0]	1.1 [0.6, 2.0]
Algeria	2018–19	3.9 [2.9, 5.2]	2.7 [2.0, 3.6]
Yemen	2013	4.6 [3.7, 5.7]	4.0 [3.0, 5.3]
Iraq	2018	2.7 [2.1, 3.6]	2.9 [2.0, 4.0]
Tunisia	2018	0.3 [0.1, 1.4]	0.7 [0.2, 2.1]
South Asia		15.3 [14.6, 15.9]	16.2 [15.5, 16.9]
Bangladesh	2019	5.7 [4.9, 6.7]	5.5 [4.7, 6.4]
Pakistan	2017–18	8.0 [5.8, 11.1]	10.5 [8.0, 13.6]
India	2019–21	19.0 [18.4, 19.6]	19.7 [19.1, 20.4]
Afghanistan	2015	8.8 [7.3, 10.7]	8.8 [7.4, 10.5]
Nepal	2019	3.7 [2.6, 5.3]	4.8 [3.4, 6.6]
Maldives	2016–17	1.1 [0.5, 2.4]	3.8 [1.6, 8.6]
West & Central Africa		10.6 [10.0, 11.3]	10.4 [9.8, 11.0]
Cote d'Ivoire	2016	9.6 [7.7, 11.9]	9.5 [7.7, 11.7]
Congo DR	2017–18	6.3 [5.0, 7.9]	8.6 [7.1, 10.3]
Congo	2014–15	5.0 [3.6, 6.8]	5.9 [4.4, 7.8]
Ghana	2017–18	9.9 [7.9, 12.3]	7.9 [6.1, 10.3]
Gambia	2019–20	7.3 [5.7, 9.2]	7.4 [5.7, 9.6]
Nigeria	2021	9.3 [8.0, 10.7]	8.3 [7.2, 9.5]
Sao Tome and Principe	2019	4.0 [2.2, 7.0]	10.2 [6.6, 15.5]
Niger	2012	14.5 [12.3, 16.9]	12.7 [10.8, 14.7]
Sierra Leone	2019	10.7 [8.8, 12.9]	10.4 [8.6, 12.5]
Mali	2018	21.9 [19.0, 25.1]	18.9 [16.5, 21.7]
Mauritania	2019–21	14.4 [12.5, 16.6]	13.5 [11.3, 16.1]
Cameroon	2018–19	7.9 [6.3, 9.8]	8.6 [6.8, 10.7]
Chad	2019	12.4 [10.8, 14.3]	14.1 [12.4, 16.1]
Senegal	2019	13.4 [10.9, 16.4]	13.5 [9.2, 19.5]
Burkina Faso	2010	16.7 [14.7, 18.9]	16.3 [14.5, 18.2]
Benin	2017–18	19.2 [17.3, 21.3]	18.5 [16.3, 20.8]
Guinea-Bissau	2018–19	14.7 [12.2, 17.5]	13.5 [11.3, 16.2]
Central African Republic	2018–19	13.8 [11.3, 16.7]	14.2 [12.0, 16.7]
Gabon	2012	2.0 [1.3, 3.1]	2.3 [1.4, 3.8]
Togo	2017	8.6 [6.5, 11.5]	9.9 [7.1, 13.5]
Guinea	2018	22.3 [19.3, 25.7]	21.1 [18.2, 24.5]
Liberia	2019–20	20.8 [16.4, 26.0]	16.5 [13.5, 20.1]

Notes: 95% confidence intervals (CI) were adjusted for clustering at the level of primary sampling units. Estimates were weighted using sampling weights rescaled to sum up the population 6–23 months old in the country and year of survey. NA is shown where countries did not have any zero-food children.

© 2024 Karlsson O et al. *JAMA Network Open*.

eTable 7. Zero-Food Prevalence: by Household Wealth Quintile

	Year	1st<br>% [95% CI]	2nd<br>% [95% CI]	3rd<br>% [95% CI]	4th<br>% [95% CI]	5th<br>% [95% CI]
Pooled		13.4 [12.8, 14.0]	11.4 [10.9, 11.9]	10.2 [9.7, 10.7]	9.3 [8.7, 10.0]	6.9 [6.3, 7.6]
East Asia & Pacific		4.5 [3.6, 5.5]	3.1 [2.3, 4.0]	2.6 [1.8, 3.8]	2.3 [1.6, 3.4]	1.9 [1.2, 2.9]
Samoa	2019–20	4.8 [2.6, 8.8]	3.2 [1.4, 7.2]	3.2 [1.3, 7.5]	5.1 [2.5, 10.1]	4.2 [1.8, 9.4]
Mongolia	2018	4.0 [1.9, 8.0]	0.9 [0.4, 2.0]	0.7 [0.2, 2.7]	0.2 [0.1, 1.0]	2.4 [0.8, 7.2]
Kiribati	2018–19	5.5 [2.9, 10.1]	2.2 [0.8, 5.5]	3.4 [1.2, 8.8]	1.0 [0.1, 7.0]	NA
Lao	2017	5.8 [4.2, 7.9]	4.5 [3.2, 6.4]	1.7 [0.9, 3.1]	1.3 [0.6, 2.8]	0.1 [0.0, 0.7]
Timor-Leste	2016	19.0 [15.1, 23.5]	19.2 [15.1, 24.0]	18.9 [14.8, 24.0]	11.7 [8.7, 15.5]	8.1 [5.0, 12.7]
Cambodia	2014	3.9 [2.3, 6.5]	3.9 [2.3, 6.5]	3.2 [1.4, 7.2]	5.0 [2.5, 9.6]	2.8 [1.2, 6.5]
Papua New Guinea	2016–18	11.2 [8.1, 15.3]	9.3 [6.3, 13.5]	7.9 [5.3, 11.7]	7.7 [5.4, 10.8]	4.0 [2.5, 6.2]
Indonesia	2017	2.8 [1.9, 4.0]	2.3 [1.5, 3.8]	2.5 [1.4, 4.3]	1.7 [0.9, 3.0]	1.7 [1.0, 3.1]
Tuvalu	2019–20	NA	NA	NA	NA	3.8 [0.5, 23.5]
Tonga	2019	0.9 [0.1, 6.3]	NA	0.9 [0.1, 6.2]	1.4 [0.2, 9.5]	2.6 [0.4, 16.8]
Viet Nam	2020–21	5.6 [3.1, 9.8]	1.3 [0.4, 3.9]	0.8 [0.1, 5.5]	2.4 [0.6, 9.1]	1.4 [0.3, 6.1]
Myanmar	2015–16	7.7 [5.2, 11.2]	6.7 [4.0, 11.2]	5.0 [2.4, 9.8]	3.6 [1.6, 8.2]	2.9 [0.9, 8.6]
Fiji	2021	2.7 [1.0, 6.9]	1.6 [0.5, 5.1]	1.1 [0.3, 4.2]	NA	0.5 [0.1, 3.5]
Eastern & Southern Africa		9.8 [8.4, 11.3]	7.9 [6.6, 9.3]	7.3 [6.0, 8.9]	8.5 [6.2, 11.5]	5.0 [2.8, 8.8]
Namibia	2013	10.1 [6.3, 16.0]	3.5 [2.0, 6.0]	8.7 [5.8, 13.0]	4.6 [2.5, 8.3]	4.4 [1.8, 10.0]
Ethiopia	2019	19.0 [13.7, 25.8]	12.5 [7.8, 19.3]	13.6 [8.3, 21.5]	19.9 [11.7, 31.9]	10.1 [3.8, 24.0]
Sudan	2014	16.7 [13.5, 20.5]	13.9 [11.5, 16.6]	11.1 [8.7, 14.0]	9.9 [6.8, 14.0]	5.3 [3.4, 8.2]
Burundi	2016–17	6.2 [4.7, 8.3]	6.0 [4.4, 8.3]	4.6 [3.3, 6.5]	4.5 [3.1, 6.5]	3.8 [2.5, 5.7]
Madagascar	2021	6.6 [4.6, 9.4]	5.1 [3.6, 7.2]	4.4 [2.8, 6.6]	3.9 [2.1, 6.9]	1.1 [0.5, 2.3]
Lesotho	2018	2.1 [0.9, 4.7]	1.7 [0.7, 4.5]	4.3 [1.6, 11.2]	1.6 [0.5, 4.8]	2.1 [0.4, 10.6]
Zambia	2018–19	7.2 [5.4, 9.5]	4.0 [2.7, 6.0]	4.8 [3.2, 7.2]	2.4 [1.2, 4.6]	2.5 [1.2, 5.1]
Mozambique	2011	4.7 [3.2, 6.9]	8.2 [5.9, 11.2]	6.1 [4.1, 9.0]	8.8 [6.2, 12.3]	4.6 [3.1, 6.8]
Rwanda	2019–20	6.9 [4.8, 9.7]	5.5 [3.7, 8.0]	5.1 [3.3, 7.8]	3.1 [1.8, 5.4]	3.9 [2.2, 6.6]
Comoros	2012	7.0 [3.7, 12.6]	6.2 [3.3, 11.4]	5.0 [2.4, 10.0]	5.6 [2.8, 11.1]	10.0 [5.0, 19.2]
Malawi	2019–20	7.5 [5.4, 10.2]	3.4 [2.3, 5.1]	4.1 [2.6, 6.5]	3.7 [2.2, 6.2]	2.3 [1.2, 4.2]
Kenya	2014	7.6 [5.7, 10.0]	5.8 [3.3, 9.9]	3.8 [2.3, 6.2]	2.3 [1.2, 4.3]	2.7 [1.4, 5.3]
Zimbabwe	2019	5.0 [3.1, 7.8]	3.6 [2.1, 6.1]	3.5 [1.7, 7.0]	2.7 [1.3, 5.4]	1.8 [0.6, 5.3]
Angola	2015–16	11.0 [8.9, 13.5]	13.6 [11.0, 16.6]	11.8 [9.4, 14.6]	9.9 [6.8, 14.2]	4.1 [2.1, 7.8]
Tanzania	2015–16	2.5 [1.4, 4.6]	3.2 [1.9, 5.3]	1.3 [0.6, 2.8]	0.8 [0.2, 2.3]	0.9 [0.2, 3.7]
Uganda	2016	8.2 [6.6, 10.2]	5.1 [3.6, 7.1]	5.8 [4.2, 7.8]	5.6 [3.8, 8.2]	3.4 [2.2, 5.2]
South Africa	2016	6.1 [3.2, 11.3]	3.4 [1.1, 10.7]	2.2 [0.9, 5.7]	7.6 [3.6, 15.4]	2.2 [0.3, 13.9]
Eswatini	2014	1.0 [0.2, 4.0]	1.5 [0.5, 4.8]	0.4 [0.1, 2.9]	0.7 [0.1, 4.9]	0.7 [0.1, 4.7]
Europe & Central Asia		4.4 [3.4, 5.7]	4.2 [2.4, 7.2]	4.1 [3.1, 5.4]	3.7 [2.7, 5.1]	3.9 [2.5, 5.9]
Albania	2017–18	10.1 [3.9, 23.8]	5.2 [2.9, 9.1]	5.1 [2.1, 12.0]	4.9 [1.5, 14.3]	7.9 [3.0, 18.9]
Montenegro	2018	4.9 [1.6, 13.7]	NA	0.8 [0.1, 5.7]	3.1 [0.7, 12.3]	NA

© 2024 Karlsson O et al. *JAMA Network Open*.

	Year	1st<br>% [95% CI]	2nd<br>% [95% CI]	3rd<br>% [95% CI]	4th<br>% [95% CI]	5th<br>% [95% CI]
Serbia	2019	NA	3.2 [0.4, 19.3]	1.1 [0.3, 4.4]	NA	NA
Armenia	2015–16	6.1 [2.6, 13.3]	3.6 [1.3, 9.3]	1.4 [0.4, 5.7]	4.7 [1.9, 11.3]	0.5 [0.1, 3.7]
Turkmenistan	2019	2.1 [0.8, 5.5]	NA	2.1 [0.8, 5.6]	0.9 [0.2, 3.5]	1.7 [0.4, 6.7]
Tajikistan	2017	12.0 [8.2, 17.2]	7.5 [5.0, 11.1]	10.6 [7.8, 14.4]	8.9 [5.9, 13.1]	7.1 [4.5, 11.0]
North Macedonia	2018–19	NA	NA	NA	NA	2.8 [0.4, 17.4]
Georgia	2018	2.5 [0.8, 8.0]	0.5 [0.1, 2.1]	0.9 [0.1, 6.3]	2.1 [0.3, 13.6]	NA
Belarus	2019	NA	0.6 [0.1, 3.8]	NA	0.9 [0.2, 3.6]	0.4 [0.1, 3.1]
Kyrgyzstan	2018	5.4 [2.9, 9.8]	2.0 [0.7, 5.4]	2.4 [0.7, 7.3]	1.5 [0.6, 4.0]	0.7 [0.2, 3.0]
Uzbekistan	2021–22	1.3 [0.3, 5.8]	3.0 [1.0, 8.7]	6.0 [2.3, 14.7]	3.4 [1.4, 7.9]	4.8 [1.7, 12.8]
Kazakhstan	2015	3.6 [1.9, 6.8]	6.6 [2.1, 18.8]	2.4 [1.2, 4.4]	4.1 [1.8, 8.9]	7.0 [3.1, 14.9]
Kosovo	2019–20	1.0 [0.1, 6.5]	NA	NA	NA	NA
Latin America & Caribbean		2.9 [2.2, 3.7]	1.5 [1.1, 1.9]	1.4 [1.0, 1.9]	2.8 [0.6, 11.1]	0.5 [0.3, 0.9]
Paraguay	2016	4.5 [2.1, 9.2]	1.5 [0.7, 3.6]	2.4 [0.7, 7.9]	0.2 [0.0, 1.6]	1.4 [0.4, 4.3]
Belize	2015–16	4.7 [1.8, 11.7]	5.4 [2.2, 12.5]	0.3 [0.0, 2.0]	2.1 [0.4, 9.6]	NA
Costa Rica	2018	0.4 [0.1, 1.6]	0.2 [0.0, 1.1]	NA	NA	NA
El Salvador	2014	3.3 [1.7, 6.0]	0.5 [0.1, 1.9]	0.6 [0.2, 2.0]	0.6 [0.2, 1.7]	0.2 [0.0, 1.1]
Mexico	2015	1.9 [0.9, 3.9]	0.5 [0.2, 1.3]	0.4 [0.1, 1.3]	4.4 [0.7, 23.8]	0.2 [0.0, 1.3]
Cuba	2019	1.3 [0.5, 3.2]	1.2 [0.5, 3.0]	2.2 [0.9, 5.0]	1.7 [0.8, 3.7]	1.3 [0.5, 3.3]
Guatemala	2014–15	5.5 [4.0, 7.6]	6.1 [4.3, 8.6]	3.0 [1.8, 4.9]	1.1 [0.5, 2.6]	1.5 [0.6, 3.6]
Dominican Republic	2019	1.3 [0.6, 2.5]	2.0 [0.6, 6.4]	1.2 [0.3, 4.6]	NA	0.6 [0.1, 2.4]
Peru	2012	4.6 [3.3, 6.5]	1.7 [1.0, 3.1]	2.3 [1.2, 4.6]	1.3 [0.5, 3.5]	0.1 [0.0, 0.5]
Guyana	2014	7.4 [4.6, 11.7]	4.6 [2.1, 9.7]	5.3 [1.9, 13.9]	NA	1.9 [0.6, 6.4]
Suriname	2018	6.9 [4.2, 10.9]	3.6 [1.2, 10.3]	NA	NA	NA
Haiti	2016–17	3.1 [1.9, 5.2]	2.8 [1.6, 5.0]	3.9 [2.0, 7.5]	3.7 [1.8, 7.7]	1.6 [0.4, 6.3]
Honduras	2019	3.7 [2.4, 5.6]	2.0 [1.0, 4.3]	1.3 [0.5, 3.4]	1.0 [0.3, 3.2]	0.6 [0.1, 3.9]
Middle East & North Africa		5.6 [4.7, 6.7]	5.2 [4.4, 6.2]	4.7 [3.8, 5.7]	4.6 [3.7, 5.8]	2.8 [2.1, 3.7]
Iraq	2018	4.4 [3.2, 6.0]	2.5 [1.7, 3.7]	2.0 [1.2, 3.4]	2.5 [1.5, 4.1]	2.3 [1.0, 5.3]
Jordan	2017–18	4.3 [2.7, 6.8]	5.4 [3.2, 8.9]	2.3 [1.1, 4.9]	2.6 [1.2, 5.8]	3.6 [1.5, 8.8]
Yemen	2013	5.1 [3.7, 7.0]	5.5 [3.7, 8.2]	5.7 [4.0, 7.9]	3.4 [2.2, 5.3]	0.9 [0.4, 2.1]
Egypt	2014	8.3 [6.1, 11.2]	8.5 [6.7, 10.7]	6.7 [5.1, 8.8]	6.9 [5.1, 9.2]	3.6 [2.5, 5.3]
State of Palestine	2019–20	1.7 [0.7, 3.9]	1.9 [0.7, 5.4]	1.5 [0.8, 3.0]	1.0 [0.5, 2.2]	1.0 [0.3, 3.2]
Algeria	2018–19	4.4 [2.9, 6.8]	2.4 [1.5, 4.0]	2.7 [1.6, 4.4]	3.2 [1.9, 5.2]	3.8 [2.0, 7.0]
Tunisia	2018	0.3 [0.0, 1.8]	0.5 [0.1, 3.8]	0.8 [0.2, 3.2]	0.7 [0.1, 4.6]	NA
South Asia		19.8 [18.7, 20.9]	17.6 [16.8, 18.6]	15.3 [14.3, 16.4]	13.5 [12.6, 14.5]	10.9 [10.0, 11.8]
Pakistan	2017–18	17.8 [13.0, 23.9]	8.5 [5.9, 12.2]	9.1 [5.4, 14.9]	7.3 [4.5, 11.6]	2.7 [1.4, 4.9]
Maldives	2016–17	1.2 [0.4, 3.8]	1.1 [0.4, 3.4]	4.3 [1.5, 11.6]	0.4 [0.1, 2.8]	4.8 [1.2, 17.5]
Nepal	2019	5.9 [4.0, 8.5]	4.9 [2.8, 8.5]	5.1 [3.2, 8.2]	3.7 [2.0, 6.6]	0.8 [0.3, 2.7]
Afghanistan	2015	8.5 [6.7, 10.8]	7.3 [5.7, 9.3]	10.1 [8.1, 12.6]	9.2 [6.9, 12.3]	8.7 [5.8, 12.9]
Bangladesh	2019	7.4 [6.0, 9.2]	6.4 [5.1, 7.9]	5.5 [4.2, 7.0]	4.7 [3.6, 6.2]	4.1 [2.9, 5.6]
India	2019–21	22.5 [21.6, 23.4]	21.9 [21.0, 22.9]	18.8 [17.9, 19.8]	16.9 [15.9, 17.9]	14.9 [13.9, 16.1]

© 2024 Karlsson O et al. *JAMA Network Open*.

	Year	1st<br>% [95% CI]	2nd<br>% [95% CI]	3rd<br>% [95% CI]	4th<br>% [95% CI]	5th<br>% [95% CI]
West & Central Africa		13.4 [12.5, 14.4]	11.6 [10.7, 12.6]	10.7 [9.9, 11.6]	9.1 [8.1, 10.1]	6.3 [5.2, 7.6]
Congo	2014–15	7.9 [6.0, 10.3]	5.1 [3.4, 7.5]	6.1 [3.6, 10.1]	4.7 [2.3, 9.1]	2.5 [1.0, 5.9]
Chad	2019	16.8 [13.9, 20.1]	12.8 [10.4, 15.7]	12.9 [10.6, 15.6]	13.0 [10.3, 16.2]	10.6 [8.0, 14.0]
Benin	2017–18	23.1 [20.0, 26.6]	20.3 [17.0, 24.0]	16.5 [13.5, 20.0]	20.3 [17.2, 23.9]	13.5 [10.7, 16.8]
Senegal	2019	13.6 [10.7, 17.3]	15.1 [11.7, 19.3]	14.4 [8.0, 24.5]	13.6 [8.7, 20.8]	9.7 [6.0, 15.3]
Ghana	2017–18	11.7 [8.8, 15.2]	13.0 [9.3, 17.9]	11.9 [8.2, 16.9]	6.5 [3.9, 10.7]	1.7 [0.8, 4.0]
Central African Republic	2018–19	13.5 [10.1, 17.7]	16.5 [13.2, 20.4]	16.6 [12.9, 21.2]	13.1 [10.0, 16.9]	8.6 [6.2, 11.8]
Mauritania	2019–21	21.6 [17.8, 25.8]	18.3 [15.1, 21.9]	12.1 [9.7, 15.0]	8.6 [6.1, 11.9]	5.0 [3.1, 7.9]
Burkina Faso	2010	16.6 [14.0, 19.6]	18.9 [16.2, 21.9]	18.0 [15.2, 21.1]	17.6 [14.7, 20.9]	9.4 [7.2, 12.3]
Mali	2018	24.5 [20.2, 29.4]	21.6 [17.7, 26.0]	22.8 [19.5, 26.6]	22.1 [17.9, 27.0]	10.2 [7.5, 13.7]
Nigeria	2021	12.7 [11.0, 14.7]	9.7 [8.0, 11.6]	7.6 [6.2, 9.3]	6.1 [4.3, 8.7]	5.7 [3.4, 9.6]
Cote d'Ivoire	2016	13.2 [10.4, 16.6]	10.8 [7.9, 14.6]	5.6 [3.8, 8.1]	10.2 [6.7, 15.1]	6.1 [3.6, 10.3]
Niger	2012	18.1 [14.5, 22.4]	14.0 [10.5, 18.4]	16.0 [12.7, 19.8]	9.8 [7.4, 13.0]	10.4 [8.3, 13.0]
Gabon	2012	4.8 [3.4, 6.7]	3.3 [1.6, 6.6]	1.2 [0.5, 3.2]	0.6 [0.2, 2.2]	0.3 [0.1, 1.4]
Liberia	2019–20	20.6 [16.8, 25.0]	22.2 [17.5, 27.8]	20.2 [14.6, 27.1]	15.4 [11.2, 20.8]	12.2 [5.8, 24.0]
Sao Tome and Principe	2019	12.8 [8.0, 19.9]	8.6 [4.0, 17.7]	4.7 [2.0, 10.9]	6.2 [2.5, 14.9]	NA
Sierra Leone	2019	11.8 [9.2, 15.0]	11.7 [9.1, 14.9]	12.2 [9.2, 15.9]	8.1 [5.5, 11.6]	7.8 [4.5, 13.3]
Guinea-Bissau	2018–19	17.0 [13.9, 20.6]	13.8 [10.5, 17.9]	16.6 [13.2, 20.7]	12.2 [8.7, 16.7]	8.3 [4.7, 14.3]
Cameroon	2018–19	12.7 [9.5, 16.9]	8.8 [6.2, 12.2]	8.3 [6.2, 11.1]	5.5 [3.5, 8.5]	3.4 [2.0, 5.7]
Guinea	2018	28.3 [23.1, 34.2]	23.5 [19.3, 28.4]	28.2 [23.2, 33.8]	19.1 [14.9, 24.3]	6.4 [4.1, 10.1]
Gambia	2019–20	11.8 [9.3, 14.8]	9.9 [7.2, 13.3]	6.4 [4.1, 9.7]	4.7 [2.6, 8.7]	2.0 [0.8, 5.3]
Congo DR	2017–18	8.9 [6.9, 11.3]	8.9 [6.7, 11.6]	8.0 [5.8, 10.8]	6.0 [4.1, 8.8]	4.3 [2.4, 7.6]
Togo	2017	11.0 [7.5, 15.8]	9.3 [6.5, 13.3]	9.0 [5.5, 14.4]	9.2 [5.1, 16.0]	7.7 [4.5, 13.0]

Notes: 95% confidence intervals (CI) were adjusted for clustering at the level of primary sampling units. Estimates were weighted using sampling weights rescaled to sum up the population 6–23 months old in the country and year of survey. NA is shown where countries did not have any zero-food children.

© 2024 Karlsson O et al. *JAMA Network Open*.

© 2024 Karlsson O et al. *JAMA Network Open*.
