# Supplementary material for: Prevalence of Children Aged 6 to 23 Months Who Did Not Consume Animal Milk, Formula, or Solid or Semisolid Food During the Last 24 Hours Across Low- and Middle-Income Countries
Source: JAMA Netw Open. 2024 Feb 12;7(2):e2355465. doi: 10.1001/jamanetworkopen.2023.55465 (PMC10862155; doi:10.1001/jamanetworkopen.2023.55465)
Supplement: Supplement 2. — Data Sharing Statement [file jamanetwopen-e2355465-s002.pdf]

Data Sharing Statement

Data
Data available: No

Additional Information
Explanation for why data not available: DHS data are available at https://dhsprogram.com and MICS data at https://mics.unicef.org (requiring a simple application).
